# Weekly high-dose liposomal amphotericin B (L-AmB) in critically ill septic patients with multiple *Candida* colonization: The AmBiDex study

**DOI:** 10.1371/journal.pone.0177093

**Published:** 2017-05-22

**Authors:** Elie Azoulay, Jean-François Timsit, Alexandre Lautrette, Stephane Legriel, Adeline Max, Stephane Ruckly, Benoit Misset, Yves Cohen, Michel Wolff

**Affiliations:** 1 Medical ICU, Saint Louis University Hospital, Paris, France; 2 Medical and Infectious Diseases ICU, Paris Diderot University/Bichat Hospital, Paris, France; 3 Medical ICU, Grenoble university hospital Albert Michallon, Grenoble, France; 4 Medical ICU, Gabriel-Montpied University Hospital, Clermont-Ferrand, France; 5 Medico-surgical ICU, Versailles Hospital, Le Chesnay, France; 6 Department of Biostatistics, OUTCOMEREA^TM^, Bobigny, France; 7 Medical ICU, Saint Joseph University Hospital, Paris, France; 8 Medical ICU, Avicenne University Hospital, Bobigny, France; Universidade do Extremo Sul Catarinense, BRAZIL

## Abstract

**Background:**

To demonstrate the feasibility and safety of weekly high-dose liposomal amphotericin B (L-AmB) (as a pre-emptive antifungal treatment) for 2 weeks in patients with septic shock and Candida colonization.

**Methods:**

Pilot, multicentre, open-label, prospective study conducted in seven French ICUs. Non-immunocompromised patients, receiving mechanical ventilation were eligible if they presented ICU-acquired severe sepsis requiring newly administered antibacterial agents and Candida colonization in at least two sites. Exclusion criteria included the need for antifungal therapy and creatinine > 220 μmol/L. All patients were to receive a high-dose L-AmB (10 mg/kg/week) for two weeks. A follow-up period of 21 days following the second administration of L-AmB was conducted. Treated patients were compared to 69 matched untreated controls admitted in the same ICUs before the study period.

**Results:**

Twenty-one patients were included in the study, of which 20 received at least one infusion of high-dose L-AmB. A total of 24 adverse events were identified in 13(61%) patients. Fourteen adverse events were categorized as serious in 8(38%) patients. In four cases the adverse events were considered as potentially related to study drug administration and resulted in L-AmB discontinuation in one patient. Few patients experienced severe renal toxicity since no patient presented with severe hypokalemia. No patients required renal replacement therapy. Compared to matched controls, no significant increase in serum creatinine levels in patients receiving high-dose L-AmB was reported.

**Conclusions:**

Weekly administration of high-dose L-AmB has a manageable safety profile and is feasible in patients with ICU-acquired sepsis and multiple Candida colonization. Trials of L-AmB versus other antifungal agents used as pre-emptive antifungal therapy are warranted.

**Trial registration:**

ClinicalTrials.gov NCT00697944

## Introduction

*Candida* is a major pathogen in critically ill patients.[1–5] The associated clinical and economic burden with Candida is high, hence explaining the increasing interest toward this pathogen.[3] Its prevalence has been estimated at 6.9 per 1000 intensive care unit stays.[6] Despite the increasing availability of antifungal agents, [7] attributable mortality rates remain high, ranging from 30% to 60% of cases,[3] It is also responsible for an increased hospital stay, directly impacting on medical costs. [7]

Definite diagnosis of invasive candidiasis remains challenging as sensitivity of blood cultures for detection of candidemia is less than 50%. Several tools have been developed to help with the early detection of patients at risk, which in turn can aid prompt initiation of treatment. However, neither the Candida score nor β-D-Glucan dosage present appropriate performances to guide therapy.[8, 9] Yet, delaying antifungal treatment for Candida bloodstream infections until a positive blood culture result is obtained may increase the risk of mortality.[10]

Prophylaxis and preemptive therapeutic strategies have been suggested in order to implement early antifungal agents in patients with either risks factors (length of intensive care unit stay, use of parenteral nutrition, broad-spectrum and long-term antibiotics use, presence of central lines, immunodeficiency, and abdominal surgery)[4, 11] or surrogate markers of infection (Candida score).[12] Strikingly septic shock patients with persistent organ failure despite any identified pathogen have been considered as target patients.[13] However, two randomized control trials in non colonized patients failed to demonstrate the benefits from antifungal therapy.[14, 15] A study of pre-emptive antifungal therapy in patients with severe sepsis, *Candida* colonization not responding to antibiotic therapy is ongoing.[16]

Liposomal amphotericin B (L-AmB) has been demonstrated as effective as conventional amphotericin B for empirical antifungal therapy in patients with fever and neutropenia. Moreover, its use was associated with fewer breakthrough fungal infections, less infusion-related toxicity and less nephrotoxicity.[17] L-AmB has the theoretical advantage of exhibiting an antifungal activity that covers the spectrum of most *Candida* species that are encountered in the ICU setting. Sequential administration of high dose L-AmB would also have several theoretical advantages, where a weekly administration would be easier to deliver and may minimize associated acute reactions. Moreover, given the pharmacokinetic and pharmacodynamics properties of high-dose L-AmB with its associated long half-life and a dose-dependent efficacy against Candida species, these delivery procedures would allow an optimization of the concentrations at the site(s) of infection(s).[18, 19] Such administration regimen was only evaluated in an hematological patient population for the prophylaxis of invasive fungal infections[20, 21], or in the treatment of invasive aspergillosis [19, 22]. It has also been studied for prophylaxis in patients undergoing liver transplantation[23], in treatment of visceral leishmaniasis in the HIV patient population[24], treatment of mucormycosis[25], or even in the neonates setting [26].

The aim of our study was to demonstrate feasibility and safety of weekly high-dose L-AmB for 2 weeks in a preemptive strategy in critically ill patients with ICU acquired sepsis and *Candida* colonization at multiple sites.

## Patients and methods

The appropriate ethics committee (*Comité de Protection des Personnes de Paris VI*) approved this prospective interventional study (N° 79–07). Written informed consent was obtained from each patient or next-of-kin before study inclusion.

### Study design

This study was a pilot, multicentre, open label, prospective study conducted in patients with multiple Candida colonization and ICU acquired sepsis. Study protocol and amendments areavailable as S1–S4 Files. Trend checklist is available as S5 File. The primary objective of this study was to evaluate the safety and tolerance of high-dose liposomal amphotericin B (L-AmB). The secondary objectives were to evaluate morbidity parameters (the length of stay in the ICU and hospital), and to assess the incidence of invasive fungal infections (IFI) according to EORTC/MSG criteria.[27] Patients were enrolled in 7 French intensive care units over a 1-year study period. Inclusion criteria were: aged over 18 years, ICU acquired-sepsis, *Candida* colonization of more than one site, a new line of antibiotic therapy, and mechanical ventilation for longer than 48 hours with at least one additional organ dysfunction. Exclusion criteria were: need for systemic antifungal therapy, patients treated with L-AmB since ICU admission, probable or proven IFI according to the EORTC/MSG criteria, SAPS II score > 65, neutropenia or marrow or solid organ transplant or chemotherapy, renal replacement therapy or serum creatinine > 220 μmol/L, moribund, decision to withdrawal or withhold life sustaining therapies, and pregnancy.

### Sample size

According to the International Conference on Harmonization E9 statistical principles for clinical trials, the predefined number of subjects had to be sufficient to assess the primary objective of the study, i.e. the assessment of the safety and tolerance of the study product. Furthermore, the assessment criterion used to evaluate this objective was only descriptive with the exhaustive presentation of adverse events occurring during the follow-up of patients. No statistical test requiring a minimum number of patients was necessary for assessment of the primary criterion of the study. Given the number of participating centers, frequency of inclusion and planned study duration, the number of subjects for this comparative pilot study was set at 30.

### Study-drug administration

All patients were to receive a high-dose liposomal amphotericin B (AmBisome^®^, Gilead Sciences, Boulogne-Billancourt, France) at a dose of 10 mg/kg/week for two weeks, except in the event of intolerance to treatment or failure thereof as per investigator's assessment. A follow-up period of 21 days was covered after the second L-AmB infusion.

High-dose L-AmB was administered intravenously over a period of approximately 2 hours with a twice slower infusion rate during the first 20 minutes of the first infusion only.

Administration of an antipyretic (paracetamol only) and an antihistamine medication (dexchlorpheniramine only) was permitted at the discretion of the Investigator. All concomitant nephrotoxic treatments were collected. If a blood culture was positive during the study or at the end of study, it was up to the decision and responsibility of the investigator to prescribe the most appropriate antifungal treatment.

### Follow-up and outcomes

All patients were followed-up to monitor any adverse events. Five parameters were specifically sought as follows: hypersensitivity, renal toxicity (hypokalemia, serum creatinine and need for renal replacement therapy), colonization, emergence of invasive fungal infection, and survival.

### Safety and tolerance evaluation

Safety and tolerance of L-AmB in patients with ICU-acquired severe sepsis were assessed by recording the incidence of related adverse events occurring during the 28-days study period. Other safety data (biological parameters, vital signs …) were also described in the analysis. Adverse events were tabulated according to “Preferred Term” and “System Organ Class” using the MedDRA classification version 11.0. Tables presented the number of patients with adverse events as well as the frequency of adverse events.

### Other outcomes

*Candida* colonization (lung, gastrointestinal tract, urine, mouth, pharynx and anus as well as the injury, wound or drain) was assessed at day 1 and then twice a week. Data on the use of antifungal agents were collected and invasive fungal infections according to the EORTC/MSG criteria were reported. Vital signs and SOFA score[28] were collected at day 1, 2, 3, 8, 14, 21 and 28 or at the end of the study. Morbidity criteria defined as length of ICU and hospital stay, and survival were also collected.

### Comparison with historical controls: Post-hoc analysis

Treated patients were compared to matched untreated controls (1 to a maximum of 5 per case) admitted in the same ICUs before the study period. Matching criteria were as follows: center, colonization at inclusion, SAPS II score ± 5 [29], absence of antifungal therapy, and length of ICU stay before receiving L-AmB.

### Statistical analysis

Quantitative parameters were described as median (interquartile range [IQR]) and qualitative parameters as number (percentages). Cases receiving high-dose liposomal amphotericin B were matched with 69 controls in a post-hoc analysis according to ICUs location, colonization at inclusion, SAPS2 score (+/-5), absence of antifungal therapy, and length of ICU stay before receiving L-AmB for cases (15 days at least for controls). Outcomes were assessed among cases and controls using a marginal Cox model: increase serum creatinine level, invasive fungal infection occurrence rates and need for additional antifungal agent. Survival was analysed using a marginal Cox model adjusted on SAPS2 and SOFA. Analyses were performed using the intent-to-treat design. All tests were two sided and *P* values <0.05 were considered significant. Analyses were performed using SAS (version 9.3; SAS Institute, Inc.).

## Results

**Fig 1:**
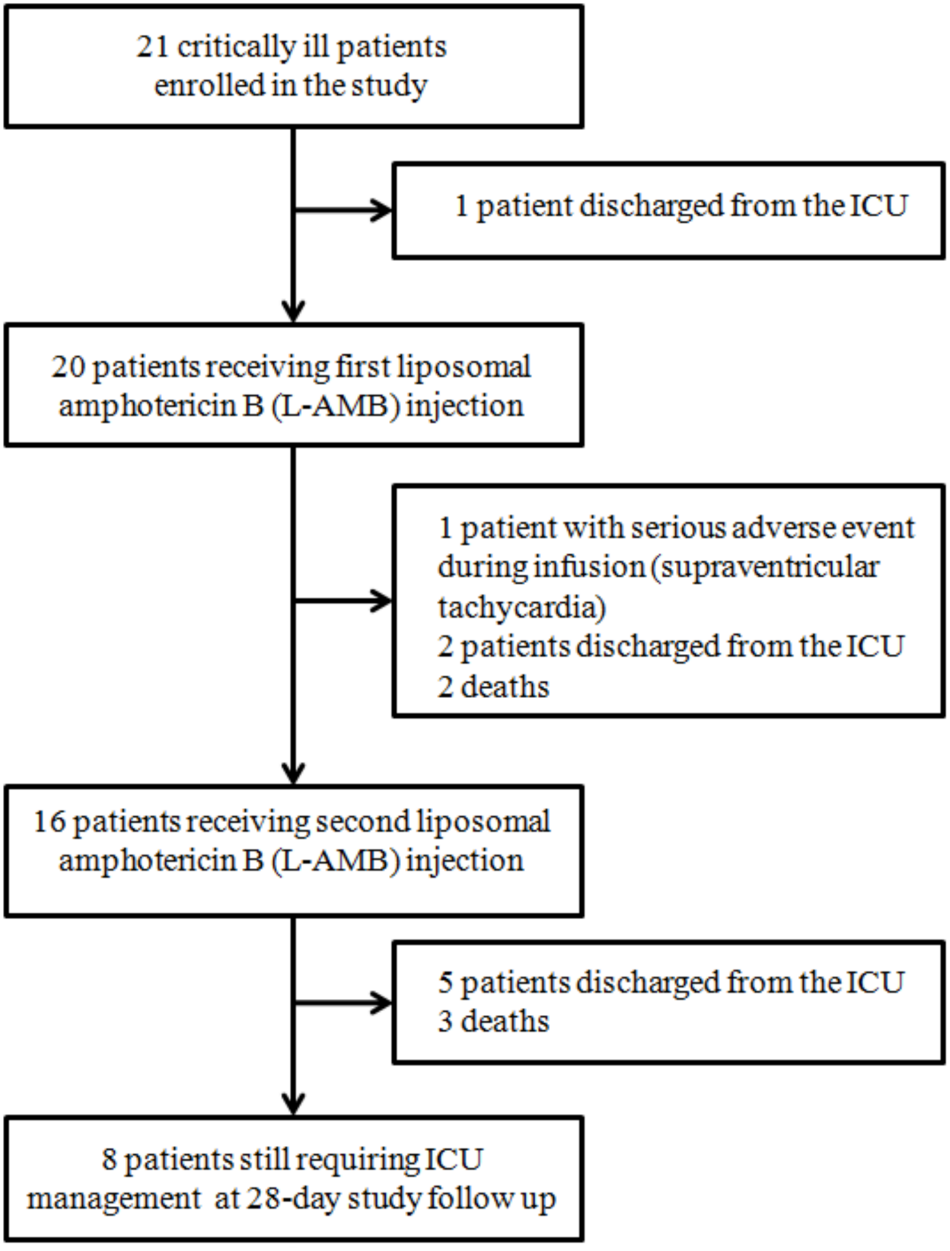
Patient flow chart, management, and 28-day follow-up in 21 patients enrolled in the study.

Fig 1 is the study flow chart. A total of 21 patients were enrolled during the 1-year study period, before discontinuation of the trial by the promoter in April 2009 due to insufficient recruitment.

### Study population at inclusion

Table 1 reports the main patient characteristics at inclusion. There were 14 men and 7 women, who were 66 years of age [IQR, 57–75]. Five patients were surgical and 16 were medical patients. Median length of ICU stay at inclusion was 11 (9–15) days and median SOFA score was 7 (3–6). All patients received mechanical ventilation. Fourteen (66%) patients presented with septic shock. Median number of Candida colonization sites was 3 (2–4). Renal function at inclusion was within normal ranges with median serum creatinine measurements of 54 (41–80) μmol/L. L-AmB administration characteristics are reported in Table 1. Seventeen (80%) patients received concomitant administration of potentially nephrotoxic treatment.

**Table 1:** Patient characteristics (n = 21).

No. (%) or Median (95% CI)	
**Demographics**	
Age (years)	66 (57–75)
Male gender	14 (66.6%)
Body Mass Index (kg/m^2^)	25.4 (20.4–27.5)
Comorbidities	
Chronic alcoholism	7 (33.3%)
Diabetes	3 (14.3%)
Corticosteroids ≥ 3 weeks in the last 2 months	2 (9.5%)
Cirrhosis	1 (4.7%)
Cancer	1 (4.7%)
Reason for ICU admission	
Medical emergency	16 (80.0)
Surgical emergency or scheduled surgery	5 (20.0)
SAPS II score at ICU admission	55 (39–68)
**Patient characteristics at inclusion**	
Length of total hospital stay at inclusion (days) ^Π^	14 (11–18)
Length of ICU stay at inclusion (days)	11 (9–15)
SOFA score at inclusion	7 (3–6)
Mechanical ventilation	21 (100%)
Septic shock Ψ	14 (66.6%)
Number of Candida colonization sites at inclusion ^Φ^	3 (2–4)
Creatinine (μmol/L)	54.0 (41.0–80.5)
Kaliemia (mmol/L)	3.6 (3.3–4.3)
Diuresis (mL/24h)	2015 (1400–2800)
Concomitant administration of nephrotoxic treatment Θ	17 (80.1%)
**L-AmB administration characteristics**	
L-AmB dosage administrated (mg/kg) at Day 1	10 (9.5–10.1)
Total volume reconstituted (mL) at Day 1	463 (340–547)
Total volume infused (mL) at Day 1	416 (310–468)
L-AmB dosage administrated (mg/kg) at Day 8	10 (10.0–10.7)
Total volume reconstituted (mL) at Day 8	463 (337–502)
Total volume infused (mL) at Day 8	463 (337–502)

Abbreviations: ICU, Intensive Care Unit; SAPS, Simplified Acute Physiology Score; SOFA; Sequential Organ Failure Assessment ***score***

^Π^ There was 10 patients previously hospitalized before ICU admission

^Ψ^ All patients received antibiotic medication for suspicion of new ICU acquired sepsis at inclusion: ventilator associated pneumonia (n = 8); surgical site infection (n = 1), bacteraemia (n = 2), undetermined (n = 10)

^Φ^ Candida colonization sites among lung (n = 15), gastrointestinal tract (n = 6), urine (n = 7), mouth/pharynx/anus (n = 15), surgical area (n = 4), other (n = 7).

^Θ^ One patient may have more than one concomitant administration of nephrotoxic treatment: antibiotics (n = 19); antihypertensive (n = 8); iodine-containing contrast media (n = 3)

### Follow-up and outcomes

#### Safety and tolerance

Tables 2 and 3 extensively lists the safety and tolerability characteristics associated with the administration of high-dose L-AmB. A total of 24 adverse events were identified in 13 (61%) patients. Fourteen adverse events were categorized as serious in 8 (38%) patients. In four cases, adverse events were considered by the investigators as potentially related to L-AmB administration. It led to definitive treatment discontinuation in one patient who experienced supraventricular tachycardia during the first infusion. Another patient experienced one hypotensive episode during the first infusion of study drug but blood pressure normalized following administration of noradrenaline and a decrease of the study drug infusion rate. The second infusion was administered according to the study procedure with no new episode. This last case was the only (5%) case of hypersensisitivy related to the administration of high-dose L-AmB. Finally, few patients experienced electrolyte disturbances or severe renal toxicity since no patient presented severe hypokalemia (<2.5 mmol/L) but five patients demonstrated an increase in serum creatinine, with a two-fold and a three-fold increase compare to baseline in 3 and 2 cases, respectively. There was no need for renal replacement therapy.

**Table 2:** Follow up and outcomes (n = 21).

No. (%) or Median (95% CI)	
**Safety and tolerance evaluation**	
At least one Adverse Event Δ	13 (61.9%)
At least one drug-related Adverse Event Λ	4 (19.0%)
At least one Adverse Event leading to study drug discontinuation ¥	2 (9.5%)
At least one Serious Adverse Event Θ	8 (38.1%)
At least one Serious Adverse Event with death	5 (23.8%)
Allergy	1 (4.7%)
Electrolyte disturbances	
Deep hypokalemia (<2.5 mmol/L)	0
Renal toxicity	
Serum creatinine increase (doubled baseline)	3 (14.3%)
Serum creatinine increase (threefold baseline)	2 (9.5%)
Need for dialysis	0
**Other Outcomes**	
Disappearance of Candida colonization	5 (23.8%)
Candidemia	1 (4.7%)
Length of ICU stay (days)	13 (8–26)
Length of hospital stay (days)	21 (10–27)
ICU mortality	5 (23.8%)

Abbreviations: ICU, Intensive Care Unit

^¥^ One patient with transient and one patient with definitive study drug discontinuation

^Δ^ Total number of adverse events (one patient may have more than one adverse event) n = 24 (thoracic disorders n = 5; infections n = 5; blood and lymphatic system disorders n = 3; general disorders n = 3; gastrointestinal disorders n = 3; cardiac disorders n = 2; vascular disorders n = 1; hepatobiliary disorders n = 1; surgical and medical procedures n = 1). Classified as mild (n = 10); moderate (n = 1); severe (n = 13).

^Λ^ Leukopaenia/neutropaenia/Thrombocytopenia n = 2; Supraventricular tachycardia n = 1; Hypotension (allergy) n = 1

^Θ^ Total number of serious adverse events (one patient may have more than one serious adverse event) n = 14 (infections n = 5; blood and lymphatic system disorders n = 2; general disorders n = 2; respiratory disorders n = 2; gastrointestinal disorders n = 2; cardiac disorders n = 1).

**Table 3:** Description of adverse events in 21 enrolled patients.

Patient	Description	SAE	Day of onset (duration)	Severity	Relationship to study drug	Action taken with study drug	28-day Outcome
**1**	Femoral and iliac thrombosis	No	D1 –D3	Severe	Unrelated	No action	Alive
	Acute non-lithiasic cholecystitis	No	D1	Severe	Unrelated	No action	Alive
**2**	No adverse event						Alive
**3**	Bilateral pleural effusion	No	D1–D28	Mild	Unrelated	No action	Alive
	ICU-acquired septic shock	Yes	D8 –D15	Severe	Unrelated	No action	Alive
**4**	ICU-acquired septic shock	Yes	D20 –D23	Severe	Unrelated	No action	Alive
**5**	Supraventricular tachycardia	No	D1	Severe	Potential link	Discontinuation	Alive
**6**	Hypotension	Yes	D1	Severe	Potential link	Decrease Infusion rate	Alive
	Refractory septic shock	Yes	D14	Severe	Unrelated	No action	Death
**7**	Haemorrhoidal bleeding	No	D5	Severe	Unrelated	No action	Alive
	Oedema in the lower limbs	No	D-6 –D19	Moderate	Not determined	No action	Alive
**8**	No adverse event						Alive
**9**	Right pleural effusion	No	D4 –D28	Mild	Unrelated	No action	Alive
**10**	Leukopaenia–neutropaenia–thrombocytopaenia	Yes	D8 –D15	Mild	Potential link	No action	Alive
	Leukopaenia–neutropaenia–thrombocytopaenia	Yes	D22–D28	Mild	Potential link	No action	Alive
**11**	Diarrhea	Yes	D3 –D7	Mild	Unrelated	No action	Alive
	Oedema	Yes	D7–D28	Mild	Unrelated	No action	Alive
**12**	No adverse event						Alive
**13**	No adverse event						Alive
**14**	No adverse event						Alive
**15**	Digestive bleeding	Yes	D7	Mild	Unrelated	No action	Alive
	Respiratory distress	Yes	D12 –D14	Severe	Unrelated	Withholding	Death
**16**	No adverse event						Alive
**17**	Anaemia	No	D1	Mild	Unrelated	No action	Alive
	Tracheotomy	No	D4	Mild	Unrelated	No action	Alive
	Epistaxis	No	D5	Mild	Unrelated	No action	Alive
	Cardiac arrest	Yes	D7	Severe	Unrelated	No action	Death
**18**	No adverse event						Alive
**19**	Respiratory distress	Yes	D25	Severe	Unrelated	No action	Death
**20**	No adverse event						Alive
**21**	Septic shock	Yes	D1 –D5	Severe	Unrelated	No action	Alive
	Death	Yes	D9	Severe	Unrelated	Withholding	Death

Abbreviations: SAE, Serious Adverse Event; D, Day

#### Other outcomes

*Candida* colonization disappeared in 5 (24%) patients during the study follow up period. Clearance of *Candida* colonization was definitive in 2 and transient in 3 cases. One patient experienced *Aspergillus fumigatus* colonization in a nasal sample on Day 3 whereas first detection was negative. Finally, only one patient presented with candidemia associated with refractory septic shock and died on Day 24. The median length of stay in the ICU and hospital were 13 (8–26) and 21 (10–27) days, respectively. Global ICU mortality was 23.8% (5 deaths). Reasons for death were as follows: ventilator acquired pneumonia, multiple organ failure, invasive candidemia with refractory septic shock, and withdrawal and withholding life sustaining treatment in two patients.

### Comparison with historical controls: Post-hoc analysis

**Fig 2:**
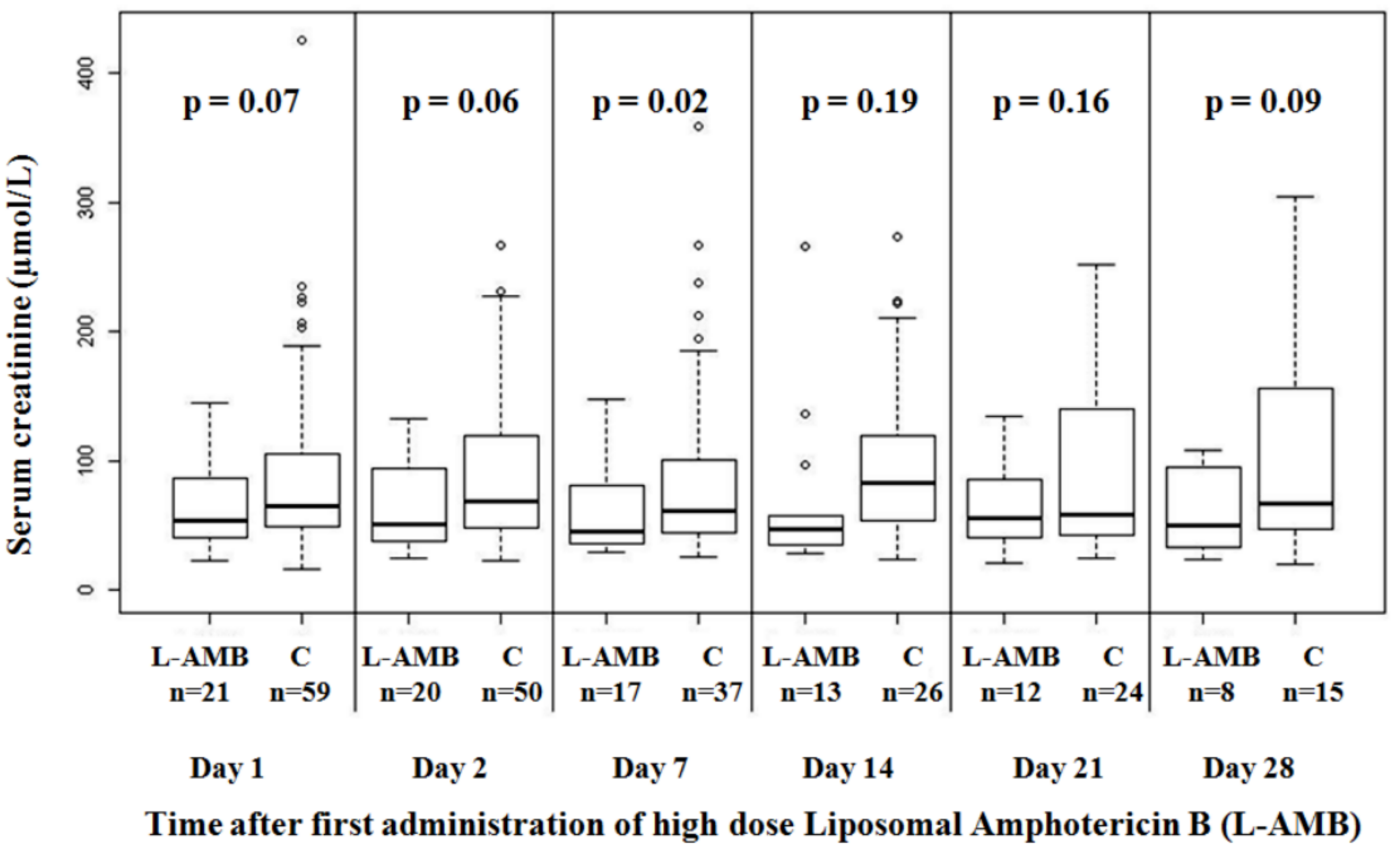
Box plots representing serum creatinine in patients receiving high-dose L-AmB and 59 matched controls. This figure shows six couples of boxplots, one couple for each visit days of follow up. The X axis shows the visit days of follow up (Day 1, Day 2, Day 7, Day 14, Day 21, and Day 28) and the Y axis the measured values of serum creatinine (μmol/L). The shaded box indicates the middle 50% of the data; the lower and upper ends of this box therefore indicate the 25^th^ and 75^th^ percentiles, respectively. The solid black horizontal line through each shaded box indicates the median of the distribution and the black cross the mean. The circles above the vertical solid black lines are individual outliers. *P* values are provided above each pair of combinations.

Table 4 shows characteristics of cases and controls at inclusion, and major outcomes in both groups. As illustrated in Fig 2, there was no significant increase in serum creatinine levels in patients receiving high-dose L-AmB compared to matched controls. Sensitivity analyses demonstrated similar results considering change of serum creatinine over 28 days, and excluding outliers from the analysis. There was no significant difference between cases and controls in the occurrences rates of invasive fungal infections (HR 0.84; 95%CI 0.10–7.30; p = 0.87) or patients in need of an additional antifungal agent (HR 0.95; 95%CI 0.34–2.67; p = 0.92). Survival adjusted on SAPS II and SOFA was not different in cases and controls (HR 0.44; 95%CI 0.1–1.83; p = 0.26). Similarly, survival adjusted on invasive fungal infections occurrence and need for antifungal agent use was not significant (HR 0.42; 95%CI 0.14–1.31; p = 0.13). The comparison with historical controls analysis had a post hoc power of 56% to demonstrate a 44% increase in the risk of death considering the mortality of the control group of 29% and an alpha risk of 5%.

**Table 4:** Characteristics and outcomes of cases treated by weekly high-dose L-AmB and matched controls.

	High dose L-AmB No. (%) or Mean (Sd) n = 21	Controls No. (%) or Mean (Sd) n = 69
**Patients characteristics at inclusion**		
Age (years)	67.5 ± 13.5	66.1 ± 16.5
Male gender	10 (47.6%)	36 (52.2%)
Reason for ICU admission		
Medical emergency	16 (76.2%)	57 (82.6%)
Surgical emergency	2 (9.5%)	8 (11.6%)
Scheduled surgery	3 (14.3%)	4 (5.8%)
SAPS II score at ICU admission	53.8 ± 16.5	54.9 ± 20.7
Length of ICU stay before inclusion (days)	14.2 ± 9.6	13.9 ± 8.7
Dialysis at inclusion	0	7 (10.1%)
Diuresis	2.4 ± 1.2	2.3 ± 1.1
SOFA score at inclusion	7 ± 3.6	5.3 ± 3.1
Severe sepsis at inclusion	14 (66.7%)	58 (84.1%)
**Outcomes**		
Invasive Fungal Infections Φ	1 (4.7%)	6 (8.7%)
ICU-acquired candidemia	0	6 (8.7%)
Use of antifungal agent Ψ	5 (23.8%)	20 (29.0%)
ICU mortality	5 (23.8%)	20 (29.0%)

Abbreviations: L-AmB, Liposomal Amphotericin B; ICU, Intensive Care Unit; SAPS, Simplified Acute Physiology Score; SOFA, Sepsis-related Organ Failure Assessment

^Φ^ Invasive Fungal Infections according to EORTC-MSG criteria

^Ψ^ In addition to L-AmB

## Discussion

In this pilot prospective multicentre study, two weekly infusions of high-dose liposomal amphotericin B demonstrated to be feasible and safe in critically ill patients presenting ICU acquired sepsis, two organ dysfunctions despite adequate antimicrobial agents and multiple Candida colonization. Manageable safety—the main objective of our study—was particularly demonstrated since the comparison with historical controls analysis showed no significant increase in serum creatinine between cases and controls.

Patient baseline characteristics and demographics were consistent with our objective to select patients with a high risk of Candida infection.[2] To this aim, our inclusion criteria strictly associated already identified predictors of invasive candidiasis in non-neutropenic critically ill patients, namely: age over 18 years old, length of ICU stay, a previous antibiotherapy, mechanical ventilation use for more than 48 hours and another associated organ failure, severe sepsis, and finally multiple colonization [2, 12, 30, 31]. As a result, our study population was comparable to other studies focusing on preemptive antifungal therapy in non-neutropenic patients with multiple-site Candida colonization.[14, 15] Length of stay prior to inclusion was 14 (11–18) days, and patients predominantly included a middle-aged male population[32] with a high rate of comorbidities.[33] Critical illness was illustrated by high SAPS II and SOFA scores at inclusion. Interestingly, the reason for ICU admission was predominantly medical in our study, while others focused on surgical critically ill patients.[31–33] All patients faced a new febrile episode for which they received a new antibiotic therapy, in association with multiple organ failure and multiple-site Candida colonization. Indeed, included population was in accordance with our preemptive antifungal therapy strategy.[34, 35]

Preemptive antifungal therapy strategy has been suggested but never yet evaluated in patients with multiple Candida colonization.[36]

The rational of this pre-emptive strategy, relies mainly on the fact that early treatment of candidemia has been proved to decrease mortality of ICU patients with septic shock.[37] However, implementation of this strategy may be associated with side effects such as overuse of antifungals, modifications of fungal ecosystem and increased antifungal resistance.[38–40] Finally, the overall benefits of this approach have never been demonstrated, and have not yet been evaluated in patients with ICU acquired sepsis and multiple Candida colonizations. Fluconazole was the most evaluated drug in retrospective[41] or prospective designs[14, 32, 42] with a focus on surgical ICU patients. Its use was associated with less Candida infections than in the control group and incidence of proven candidiasis was significantly reduced.[32] Caspofungin demonstrated similar results using a different design while patients received first prophylaxis before the preemptive strategy.[15] Nevertheless, given the spectrum of Candida species encountered in the ICU[43, 44] and their sensibility with available antifungal therapies, L-AmB would theoretically be among the best choices. It has been demonstrated that the over usage of new antifungal agents have been responsible for the emergence of Candida species with increased minimum inhibitory concentrations.[45] Thus azole derivatives and echinocandins are now associated with decreased susceptibility and resistance to Candida species[39, 40] and particularly *Candida glabrata*[46–48]. With these recent developments, and the proportion of *Candida albicans* and *non-albicans*, the use of L-AmB may be favoured in this instance. However, L-AmB has been only evaluated in this setting in patients with persistent fever and neutropenia[17] and was demonstrated as effective as conventional amphotericin B, being associated with fewer infusion-related toxicity and fewer nephrotoxicity.

High dose L-AmB has previously been tested in the haematologic patient population.[20, 21] In prophylaxis of invasive fungal infection following chemotherapy, high-dose L-AmB was demonstrating a manageable safety profile, associating transient reactions that were reversible by stopping the infusion. In patients with invasive aspergillosis, compared to a bi-therapy strategy associating L-AmB at standard dosage with caspofungin, high-dose L-AmB was associated with more infusion-related reactions and serum creatinine impairment. [49] In a large double blind randomized trial, pertaining to the treatment of invasive mold infection in immunocompromised patients, no benefit was observed for high-dose L-AmB in comparison with the standard dosage, as this was associated with a higher rate of nephrotoxicity and no significant improvement in efficacy.[22] In another immunocompromised setting of patients undergoing liver transplantation, high-dose L-AmB was delivered for the prophylaxis of invasive fungal infections. Its use was well tolerated. Even if few patients encountered renal injury, it was not directly attributed to L-AmB. Moreover, only 2 (3%) patients experienced invasive candidiasis.[23] In a different setting of Ethiopian patients, a single high-dose L-AmB was administered for the treatment of visceral leishmaniasis with success and without significant adverse effects.[24] Finally, high-dose L-AmB was used as treatment of mucormycosis[25], or even in the neonates setting [26].

In the present study, weekly high-dose L-AmB administration showed a manageable safety profile. All but one patient received a first high-dose infusion of L-AmB and 80% of patients received a second infusion. Adverse events were sought as potentially linked to L-AmB administration in only four cases. Infusion discontinuation was made for only one patient. In another case, a decrease of infusion rate associated with symptomatic measures allowed full administration of first dose. Finally, only five patients experienced severe renal toxicity. They demonstrated an increase in serum creatinine. No patient presented with severe hypokalemia (<2.5 mmol/L). Interestingly, there was no significant increase in serum creatinine levels in patients receiving high-dose liposomal amphotericin B when compared to matched controls. Renal replacement therapy was not required. In addition, while our study was not designed to evaluate efficacy, only 5% (1/20) of patients experienced invasive candidemia. Given the broad heterogeneity in published studies that aimed at evaluating preemptive antifungal therapy in the ICU in non-neutropenic patients, reported rates of candidemia vary from one study to each other. In the surgical intensive care unit setting, fluconazole allowed a significant lower rate of candidemia in comparison with control, encountered in 2% and 18% respectively.[33] In another context of intensive care unit patients receiving mechanical ventilation for at least 3 days in addition to the presence of a central line and other risk factors, the use of caspofungin in a placebo double blind randomized design trial was associated with a lower rate of invasive candidiasis although encountered in 19% of cases.[15]. Comparably with these studies, mortality rate remained of 25% in our study and without influence of the intervention after adjustment on severity (p = 0.25). There was a trend for lower adjusted survival without invasive fungal infections and no need for an additional antifungal agent.

Our study has several limitations. First, the major limitation of our study is the lack of a properly randomized control group. Considering the severe critical illness of enrolled patients, the impact on outcome of adverse events appears difficult to appreciate. The comparison we performed after matching with a historical control group was aimed at solving this problem, but selection bias and other systematic errors inherent to this procedure may have not been sufficient to balance patient’s characteristics at baseline. Second, the extent to which our findings apply to the full spectrum of patients with suspected risk of invasive candidiasis is unclear. Nevertheless, we targeted our inclusion criteria to a subset of ICU patients associating most of identified risk factors of invasive candidiasis otherwise surgical ICU patients. We can assume that our strategy was effective since our population was comparable to those of others studies dealing with that field. Third, one can argue that our recruitment was restricted to only 20 cases. Indeed, the objective of our prospective pilot study was safety evaluation of high-dose L-AmB. Thus, as mentioned above, we focused on a specific population. Furthermore, included cases were matched with 69 controls allowing comparison using a marginal Cox model. Fourth, one of our exclusion criteria was violated since we included 7 patients with a SAPS II score > 65. However, our comparing population was matched according several criteria including SAPS II and survival analyses were performed with adjustment on SAPS2 and SOFA, minimizing the impact patient’s severity at inclusion.

Fifth, all but one patient received high-dose infusion of L-AmB. Given our intention to treat statistical analysis design, all included patients entered the analysis process.

### Conclusion

Two weekly high-dose infusions of liposomal amphotericin B demonstrated has a manageable safety profile and is feasible in critically ill patients associating ICU acquired sepsis, two organ dysfunctions despite adequate antimicrobial agents and multiple *Candida* colonizations. Moreover, other points of interest such as renal injury and overall outcomes were unremarkable. Trials of L-AmB versus other antifungal agents used as pre-emptive antifungal therapy are warranted.

## Supporting information

S1 File Protocol French version. (PDF)

S2 File Protocol first amendment. (PDF)

S3 File Protocol second amendment. (PDF)

S4 File Protocol English version. (PDF)

S5 File Trend checklist. (PDF)

## Abbreviations

EORTC/MSG: Fungal Infections Cooperative Group and the National Institute of Allergy and Infectious Diseases Mycoses Study Group
HR: Hazard Ratio
ICU: Intensive Care Unit
IFI: Invasive Fungal Infections
L-AmB: Liposomal Amphotericin
MedDRA: Medical Dictionary for Regulatory Activities
SAPS II: Simplified Acute Physiology Score II
SOFA: Sepsis-related Organ Failure Assessment
