# Supplementary material for: Weekly high-dose liposomal amphotericin B (L-AmB) in critically ill septic patients with multiple Candida colonization: The AmBiDex study
Source: PLoS One. 2017 May 22;12(5):e0177093. doi: 10.1371/journal.pone.0177093 (PMC5439673; doi:10.1371/journal.pone.0177093)
Supplement: S1 File — (PDF) [file pone.0177093.s001.pdf]

Etude AmBiDex

Traitement préemptif des colonisations multiples à *candida* chez des patients de réanimation présentant un sepsis

- Protocole d'étude clinique -

**Promoteur** :	FOVEA SA<br>3 bis chemin de la Jonchère<br>92500 ~ Rueil-Malmaison, France<br>Tél : 01 47 14 04 95<br>Fax : 01 47 16 11 10
**Investigateur Coordonnateur** :	Professeur Elie AZOULAY<br>Service de Réanimation Médicale<br>Hôpital Saint Louis<br>1 avenue Claude Vellefaux<br>75010 ~ PARIS<br>Tél : 01 42 49 94 21<br>Fax : 01 42 49 94 26<br>Courriel : elie.azoulay@sls.ap-hop-paris.fr
**Conseiller scientifique** :	Professeur Michel WOLFF<br>Clinique de Réanimation des Maladies Infectieuses<br>Hôpital Bichat−Claude Bernard<br>46, rue Henri Huchard<br>75010 ~ PARIS<br>Tél : 01 40 25 77 03<br>Fax : 01 42 26 64 38<br>Courriel : michel.wolff@bch.ap-hop-paris.fr

CALENDRIER DE L'ÉTUDE

Début prévisionnel de l'étude : Septembre 2007

Fin prévisionnelle de l'étude : Mars 2008

CONFIDENTIEL

Propriété exclusive du Groupe FOVEA. Ne doit être communiqué, hors du groupe, qu'aux personnes qui, de par leur fonction et/ou leur rôle dans l'étude, doivent avoir accès au contenu intégral du protocole.

STRUCTURE ADMINISTRATIVE DE L'ÉTUDE

**Titre**	Traitement préemptif des colonisations multiples à *candida* chez des patients de réanimation présentant un sepsis
**Produits étudiés**	AmBisome® (amphotéricine B liposomale)
**Indication**	Traitement préemptif des colonisations multiples à *candida* chez des patients de réanimation présentant un sepsis
**Phase**	Etude pilote de faisabilité
**Investigateur Coordonnateur**	Professeur Elie AZOULAY<br>Hôpital Saint-Louis - Paris
**Investigateur Principal**	Professeur Michel WOLFF<br>Hôpital Bichat - Paris
**Promoteur & Monitoring de l'étude**	FOVEA SA<br>Rueil-Malmaison
**Début de l'étude**<br>**Fin de l'étude**	Septembre 2007<br>Mars 2008
**Centres participants**	Essai multicentrique national : services de réanimation

PAGE DE SIGNATURES

Nom, Fonction	Date	Signature
**Professeur Elie Azoulay**<br>Investigateur coordonnateur	29/8/2007	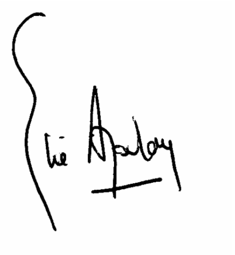
**Professeur Michel Wolff**<br>Conseiller scientifique		
**Docteur Franck Sévenier**<br>Président Directeur Général – Fovea		
**Docteur Jean-François Dreyfus**<br>Chef de Projet clinique<br>Responsable Pharmacovigilance	21/8/2007	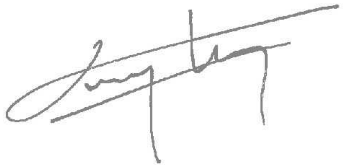

SYNOPSIS

**Titre de l'étude**	AmBiDex – Traitement préemptif des colonisations de multiples sites à *candida* chez des patients en soins intensifs présentant un sepsis
**Justification**	La candidémie est associée à une mortalité élevée or, il n'existe pas de procédure diagnostique qui permette d'anticiper la survenue de cette pathologie, mais il a été démontré, chez les patients chirurgicaux en soins intensifs, qu'il existait un continuum qui va de la colonisation à l'infection par *candida*. Il est donc urgent de développer des stratégies thérapeutiques lorsqu'un patient présente des colonisations de plusieurs sites qui coexistent avec des facteurs de risque comme la défaillance de plusieurs organes (y compris la nécessité d'une ventilation assistée) et un état septique nosocomial sévère.<br>La quasi−totalité des patients qui ont présenté une candidémie présentaient une colonisation par *candida*. et il semble que la colonisation soit associée à une prolongation de la durée du séjour en soins intensifs.<br>L'hypothèse de base de cette étude est que les patients en Unité de Soins Intensifs présentant une colonisation de plusieurs sites ont un risque élevé de candidose invasive et que l'administration précoce d'un agent antifongique empêchera l'apparition d'une candidose invasive.<br>Une administration hebdomadaire d'AmBisome® pourrait représenter une option thérapeutique importante que l'on peut justifier sur la base des résultats pharmacocinétiques et du profil pharmacodynamique, car elle associe un schéma d'administration simple à un large spectre d'activité thérapeutique.
**Nombre de centres prévus**	7 centres en France

**Objectif**	<u>Principal</u> :<br>▪ Tolérance et sécurité d'emploi de l'AmBisome® 10 mg/kg en une administration hebdomadaire (2 perfusions à une semaine d'intervalle)<br><br><u>Secondaires</u> :<br>• Évaluation des paramètres de morbidité suivants :<br>Durée de séjour dans l'Unité de Soins Intensifs<br>Durée de séjour à l'hôpital<br>Nombre de jours sans défaillance d'organe<br>• Incidence des infections fongiques invasives selon les critères de l'EORTC-MSG modifiés<br>• Incidence d'autres infections nosocomiales subséquentes<br>• Taux de mortalité à 28 jours
**Plan expérimental**	Étude pilote, multicentrique, réalisée en ouvert
**Nombre de patients**	30
**Population cible**	Patients en unité de soins intensifs présentant une colonisation de plusieurs sites par *candida* dans le contexte d'une défaillance d'au moins deux organes (y compris la nécessité d'une assistance respiratoire) et d'un état septique nosocomial sévère
**Traitement**	Ambisome 10 mg/kq
**Durée du traitement**	2 perfusions à une semaine d'intervalle
**Diagnostic et principaux critères d'éligibilité**	1. Age ≥ 18 ans<br>2. Ventilation depuis plus de 48 heures<br>3. Patients présentant une défaillance d'organe en plus de la ventilation mécanique<br>4. Colonisation à *candida* d'au moins 2 sites dont 1 non digestif<br>5. Suspicion de sepsis nosocomial<br>6. Mise en place d'une nouvelle antibiothérapie pour la suspicion de sepsis nosocomial<br>7. Consentement de la personne de confiance au moment de l'inclusion

**Critères de non−inclusion**	1. Traitement par antifongiques par voie orale (décontamination digestive sélective) ou par voie systémique dans les 15 jours précédant l'inclusion<br>2. Patients répondant aux critères de mise en œuvre d'un traitement antifongique classique<br>3. Patients moribonds (SAPS à l'inclusion >65)<br>4. Patients neutropéniques ou receveurs d'une greffe de cellules souches hématopoïétiques ou d'organe solide ou bénéficiant d'une chimiothérapie<br>5. Présence d'une infection fongique documentée (prouvée ou probable) selon les critères de l'EORTC/MSG modifiés<br>6. Créatininémie > 220 µmol/L sans dialyse<br>7. Grossesse

**Informations recueillies et procédures**	V1 (J1)<br>Consentement éclairé d'un proche<br>Données démographiques<br>Vérification des critères d'inclusion et de non inclusion (comorbidités, critères de défaillance, score SOFA, état septique)<br>Signes vitaux avant et après perfusion<br>Examens de laboratoire<br>Facteurs de risque (colonisation des sites habituellement surveillés dans le service)<br>Test de grossesse si applicable<br>Traitements concomitants<br>*Administration du produit à l'étude (durée, quantité administrée, procédure)*<br>Evènements indésirables<br>Survie<br><br>Recueil des informations sur la colonisation fongique des sites testés à V1, 2 fois par semaine<br><br>V2 (J2)<br>Signes vitaux, score SOFA, critères d'un sepsis, examens de laboratoire<br>Survenue d'infection fongique invasive, survie<br>Traitements concomitants, évènements indésirables<br><br>V3 (J3)<br>Signes vitaux, score SOFA, critères d'un sepsis, examens de laboratoire<br>Survenue d'infection fongique invasive, survie<br>Traitements concomitants, évènements indésirables<br><br>V4 (J8)<br>Signes vitaux, score SOFA, critères d'un sepsis, examens de laboratoire<br>Survenue d'infection fongique invasive, survie<br>Traitements concomitants, évènements indésirables<br>*Administration du produit à l'étude*<br>Événements indésirables au cours et au décours de la perfusion<br><br>V5 (J14)<br>Signes vitaux, score SOFA, critères d'un sepsis, examens de laboratoire<br>Survenue d'infection fongique invasive, survie<br>Traitements concomitants, évènements indésirables<br><br>V6 (J21)<br>Signes vitaux, score SOFA, critères d'un sepsis, examens de laboratoire<br>Survenue d'infection fongique invasive, survie<br>Traitements concomitants, évènements indésirables<br><br>V7 (J28 ou J SORTIE) VISITE FINALE<br>Signes vitaux, score SOFA, critères d'un sepsis, examens de laboratoire<br>Survenue d'infection fongique invasive, survie<br>Traitements concomitants, évènements indésirables

SYNOPSIS (Version anglaise)

**Title of Study**	AmBisom® Loading-dose regimen in early Treatment of Multiple-Site *candida* Colonization in Critically ill Patients with Severe Sepsis - AmBidex Study
**Rationale**	Candidemia is associated with a high mortality and is a major preoccupation in ICU. Diagnostic procedures are not predictive of this pathology but the continuum from colonization to infection by *candida* in surgical ICU patients has been demonstrated. There is an urgent need for developing early strategies in multiple-site colonized patient, and even more so in patients with other risk factors such as organ failures (including a need for mechanical ventilation), and severe nosocomial sepsis.<br>*Candida* colonization has been found in in most patients with candidemia and may be associated with a longer ICU stay.<br>The main study hypothesis is that ICU patients with multiple site *candida* colonization are at high risk for invasive candidiasis and that early administration of an antifungal agent ensures effective treatment of potential invasive candidiasis although currently available tests for systemic candidiasis are unable to confirm the diagnosis.<br>There is a medical need to provide appropriate early treatment especially to ICU patients with comorbid conditions.<br>Weekly dosing of AmBisome® should provide an important therapeutic option, as a result of the PK/PD profile, providing a more convenient dosing schedule and a broad spectrum of action.
**Study Centers Planned**	7 centers in France
**Objectives**	Primary objective:<br>Safety and tolerability of AmBisome® 10 mg/kg in a weekly administration (2 week-treatment)<br><br>Secondary objectives:<br>• Evaluation of morbidity parameters as listed in the following:<br>- ICU stay length<br>- hospital stay length<br>- number of days without organ failures<br>• Incidence of invasive fungal infections according to modified EORTC-MSG criteria<br>• Incidence of other subsequent nosocomial infections<br>• Mortality rate at Day 28
**Study Design**	Pilote, multicenter, randomized, open trial
**Number of Patients Planned**	30

**Target Population**	ICU patients with Multiple-Site *candida* Colonization and organ failures (including need for mechanical ventilation) and severe nosocomial sepsis
**Treatment**	10 mg/kg infusion
**Duration of Treatment**	2 infusions at 1−week interval
**Diagnosis and Main Eligibility Criteria**	1. Age ≥18 years<br>2. Mechanical ventilation for longer than 48 hours<br>3. At least one organ failure in addition to respiratory failure requiring mechanical ventilation<br>4. Candida colonization at least one or more site in addition to the gastrointestinal tract<br>5. Suspicion of nosocomial sepsis<br>6. Initiation of a new antibiotherapy for this suspected nosocomial sepsis<br>7. Consent from the trustworthy person at inclusion visit
**Non−inclusion criteria**	Oral or systemic antifungal treatment (selective digestive decon-tamination) in the 15 days before inclusion<br>Patients for whom a standard antifungal treatment is indicated<br>Moribund patients moribonds (SAPS at inclusion > 65)<br>Patients with neutropenia or that have received a hematopoietic stem−cell graft or a solid organ graft or chemotherapy<br>Patients with a documented fungal infection (proven or probable) according to modified EORTC/MSG<br>Creatininemia superior to 220 mL off dialysis<br>Pregnancy

**Data recorded and procedures performed**	V1 (D1)<br>Informed consent of relative<br>Demographic data<br>Check on inclusion and non inclusion criteria (comorbidities, organ failures, SOFA score, sepsis)<br>Vital signs (before and after infusion)<br>Laboratory workup<br>Risk factors (colonisation of sites routinely assessed in ICU)<br>Pregnancy test (if applicable)<br>Concomitant treatmenes<br>*Study drug infusion (duration, dose, procedure)*<br>Adverse events<br>Survival<br><br>Recueil des informations sur la colonisation fongique des sites testés à V1, 2 fois par semaine<br><br>V2 (D2)<br>Vital signs, SOFA score, sepsis criteria, laboratory workup<br>Occurrence of an invasive fungal infection, survival<br>Concomitant treatments, adverse events<br><br>V3 (D3)<br>Vital signs, SOFA score, sepsis criteria, laboratory workup<br>Occurrence of an invasive fungal infection, survival<br>Concomitant treatments, adverse events<br><br>V4 (D8)<br>Vital signs, SOFA score, sepsis criteria, laboratory workup<br>Occurrence of an invasive fungal infection, survival<br>Concomitant treatments, adverse events<br>*Study drug administration*<br>Adverse events durng and after infusion<br><br>V5 (D14)<br>Vital signs, SOFA score, sepsis criteria, laboratory workup<br>Occurrence of an invasive fungal infection, survival<br>Concomitant treatments, adverse events<br><br>V6 (D21)<br>Vital signs, SOFA score, sepsis criteria, laboratory workup<br>Occurrence of an invasive fungal infection, survival<br>Concomitant treatments, adverse events<br><br>V7 (D28 or TERMINATION DAY) FINAL VISIT<br>Vital signs, SOFA score, sepsis criteria, laboratory workup<br>Occurrence of an invasive fungal infection, survival<br>Concomitant treatments, adverse events

LISTE DES ABRÉVIATIONS

AFSSAPS	Agence Française de Sécurité Sanitaire des Produits de Santé
AMM	Autorisation de Mise sur le Marché
AUC	Area Under Curve (Aire Sous la Courbe)
BPC	Bonnes Pratiques Cliniques
Bpm	Beat per minute (Fréquence par minute)
CAB	Conventional amphotericin B (Amphotéricine B conventionnelle)
C_{max}	Concentration maximale
CNIL	Commission Nationale de l'Informatique et des Libertés
CRF	Case Report Form (Cahier d'observation)
EIG	Evénement Indésirable Grave
EORTC	European Organisation on Research and Treatment of Cancer (Organisation Européenne de Recherche et de Traitement du Cancer)
HAD	Hospitalisation à domicile
HJ	Hôpital de jour
ICH	International Conference on Harmonisation (Conférence Internationale d'Harmonisation [des processus et documents destinés à l'enregistrement])
ICU	Intensive Care Unit (Unité de Soins Intensifs)
IFI	Infections Fongiques Invasives
ITT	Intention de Traitement
MTD	Maximal Tolerated Dose (Dose Maximale Tolérée)
NEJM	New England Journal of Medicine
NFS	Numération Formule Sanguine
NGAP	Nomenclature Générale des Actes Professionnels
PD	PharmacoDynamic
PK	PharmacoKinetic (Pharmacocinétique)
PNN	PolyNucléaires Neutrophiles
PU-PH	Professeur des Universités – Praticien Hospitalier
RCP	Résumé des Caractéristiques du Produit

SAPS	Simplified Acute Physiology Score (Score Aigu Simplifié de Physiologie)
SOFA	Sequential Organ Failure Assessment (Évaluation Séquentielle des Défaillances d'Organes)

TABLE DES MATIERES

STRUCTURE ADMINISTRATIVE DE L'ÉTUDE **2**

PAGE DE SIGNATURES **3**

SYNOPSIS **4**

SYNOPSIS (Version anglaise) **8**

LISTE DES ABRÉVIATIONS **11**

TABLE DES MATIERES **13**

1. INTRODUCTION **15**

1.1. CONTEXTE MÉDICAL 15
1.2. CANDIDOSES ET RÉANIMATION : FORMES CLINIQUES ET PRONOSTIC 16
1.3. THÉRAPEUTIQUES ANTIFONGIQUES PRÉVENTIVES CHEZ LES PATIENTS NON NEUTROPÉNIQUES DE RÉANIMATION 17
1.4. AMPHOTÉRICINE B LIPOSOMALE : AMBISOME® 19
1.5. PROBLÉMATIQUE DE L'ÉTUDE 22
1.6. OBJECTIFS DE L'ÉTUDE 23

2. MÉTHODOLOGIE DE L'ETUDE **24**

2.1. DESIGN GÉNÉRAL DE L'ÉTUDE 24
2.2. SCHÉMA 25
2.3. DISCUSSION SUR LE PLAN EXPÉRIMENTAL 25
2.4. COMITÉ DE PILOTAGE 26

3. POPULATION ÉTUDIÉE **27**

3.1. CRITÈRES D'INCLUSION 27
3.2. CRITÈRES DE NON INCLUSION 27

4. MODALITÉS DE TRAITEMENT **28**

4.1. PRODUIT ADMINISTRÉ 28
4.2. TRAITEMENT À L'ÉTUDE : AMBISOME® 28
4.3. DOSAGE ET ADMINISTRATION D'AMBISOME® 31
4.4. DISPONIBILITÉ ET DISPENSATION DES UNITÉS THÉRAPEUTIQUES 33
4.5. TRAITEMENTS CONCOMITANTS 34

4.6. COMPLIANCE 34
4.7. PATIENTS EXCLUS EN COURS D'ÉTUDE ET ARRÊT PRÉMATURÉ DE TRAITEMENT 35

5. VISITES ET PROCÉDURES 36

5.1. V1 (J1) : VISITE D'INCLUSION ET DE PREMIERE PERFUSION 36
5.2. V2 (J2) : PREMIÈRE VISITE DE SUIVI DE LA PREMIERE PERFUSION 37
5.3. V3 (J3): DEUXIÈME VISITE DE SUIVI DE LA PREMIERE PERFUSION 37
5.4. V4 (J8) : VISITE DE DEUXIEME PERFUSION 37
5.5. V5 (J14) VISITE POST TRAITEMENT 38
5.6. V6 (J21) VISITE POST TRAITEMENT 38
5.7. V7 (J28 OU J SORTIE) : VISITE FINALE 38
5.8. RECUEIL DES INFORMATIONS TOUT AU LONG DE L'ÉTUDE 39

6. EVALUATION DE LA TOLÉRANCE 40

6.1. DÉFINITION 40
6.2. EVÉNEMENTS INDÉSIRABLES 40
6.3. EVALUATION DE L'EFFICACITÉ 44

7. MÉTHODOLOGIE STATISTIQUE 45

7.1. CALCUL DU NOMBRE DE SUJETS NÉCESSAIRES 45
7.2. EXPLOITATION STATISTIQUE 45

8. RESPONSABITILITÉS DU PROMOTEUR 47

8.1. CONDUITE DE L'ESSAI 47
8.2. CONSIDÉRATIONS ÉTHIQUES 47
8.3. ORDRE DES MÉDECINS 47
8.4. LOI INFORMATIQUE ET LIBERTÉ 47
8.5. FOURNITURE DES MÉDICAMENTS 49
8.6. CONDITIONS DE PUBLICATION 49
8.7. ARCHIVAGE 49

9. REFERENCES BIBLIOGRAPHIQUES 50

1. INTRODUCTION

1.1. CONTEXTE MÉDICAL

En dépit des nombreux facteurs de risque de candidoses invasives que présentent les patients de réanimation (1, 2), la candidémie y est un événement rare. En effet, sur 1.000 patients admis en réanimation seulement 2 présenteront une candidémie (3, 4), ce chiffre pouvant aller jusqu'à 10 chez les patients de réanimation chirurgicale (5, 6). Ces chiffres restent aussi très différents de ceux publiés en Amérique du Nord (7).

Néanmoins, ces dernières années, les réanimateurs ont montré un intérêt tout particulier pour l'infection fongique et les thérapeutiques antifongiques. Les cinq éléments suivants soulignent la pertinence clinique de l'infection fongique en réanimation, et expliquent le paradoxe entre un événement peu fréquent (la candidose invasive) et cet engouement particulier de la part des réanimateurs pour la pathologie liée au *candida* :

1) La mortalité liée aux candidoses invasives est élevée, avec une mortalité attribuable importante, suggérant une probable supériorité du traitement préventif sur le traitement curatif (4, 8) ;
2) L'hémoculture positive à *candida* (la candidémie à proprement parler) est un événement tardif, peu sensible, ce qui souligne la nécessité de stratégies préventives couplées à la recherche d'une stratégie diagnostique précoce inexistante à ce jour (2) ;
3) Si la majorité des candidémies sont provoquées par *Candida albicans*, le programme SENTRY et l'étude NNIS ont souligné l'émergence de souches de sensibilité réduite au fluconazole (*C Glabrata et C Kruseï*) (7, 9, 10, 11, 12). Ces résultats semblent toutefois moins préoccupants en Europe (13) ou le fluconazole reste encore une molécule de première intention (14, 15) ;
4) Pittet et collaborateurs ont démontré en 1994 le continuum colonisation – infection à *candida* chez les patients de réanimation chirurgicale et ont proposé un index de colonisation (nombre de sites colonisés sur nombre de sites prélevés), facteur de risque indépendant de candidémie avec une souche génotypiquement identique à celle qui colonisait le patient les jours précédents (16). Néanmoins, ces résultats n'ont pas été démontrés dans d'autres études depuis la publication princeps et plus particulièrement, pas chez des patients de réanimation médicale.

Cependant, une étude récente souligne la relation entre les colonisations multiples (surtout pulmonaire et digestive) à *candida* et les candidémies chez des patients non neutropéniques de réanimation médicale (17) ;

5) Au cours des dernières années, des études cognitives ont démontré la pathogénicité de *candida* dans la cavité péritonéale des patients de réanimation chirurgicale opérés de perforations digestives, pancréatites nécrosantes, ou de lâchages de sutures (18, 19, 20). Dans le même sens, des facteurs de risque ont été identifiés (21) et des thérapeutiques prophylactiques ont été validées (22). La présence de *candida* dans le péritoine apparaît singulièrement différente de la présence de *candida* dans le poumon (23-25) et les urines (26).

Les points évoqués ci−dessus soulignent l'intérêt potentiel d'un traitement antifongique prophylactique ou précoce (« préemptif ») chez les patients de réanimation non immunodéprimés.

1.2. CANDIDOSES ET RÉANIMATION : FORMES CLINIQUES ET PRONOSTIC

Parmi les affections invasives liées à *candida*, aucune forme clinique n'est spécifique à la réanimation. Ce qui caractérise les candidoses invasives en réanimation c'est leur rareté (4, 27, 28) et leur sévérité, attestant du besoin de suppléance des défaillances d'organes, chez des patients ayant plus fréquemment des comorbidités. De nombreux auteurs ont souligné le caractère tardif de la candidémie et l'absolue nécessité de développer une stratégie thérapeutique préventive, concomitamment à la recherche en cours sur des techniques de diagnostic immunologique rapide, encore décevantes à ce jour (2, 27, 28, 29).

Chez le patient non neutropénique de réanimation, la colonisation multiple préalable à l'infection fongique semble caractériser le malade de réanimation chirurgicale. Aussi, si l'infection de cathéter à *candida* est plus fréquemment retrouvée en réanimation, dire s'il s'agit d'une localisation secondaire de la fongémie ou de l'origine de celle ci reste hasardeux. L'ablation de tout cathéter est en tout cas systématique.

La colonisation à *candida* a fait l'objet de plusieurs études descriptives en réanimation chirurgicale : elle est présente chez plus de la moitié des patients (29), chez la majorité des patients présentant une candidémie (30), et serait associée à une augmentation de la durée de séjour en réanimation (29). C'est essentiellement *candida albicans* qui est responsable de cette colonisation (16, 29, 30), les espèces de sensibilité réduite aux antifongiques (*candida glabrata* et *candida kruseï*) ne représentant qu'à peu près 10% des cas. Concernant cette colonisation trois éléments doivent être rappelés : tout d'abord, cette colonisation est le plus souvent asymptomatique, elle doit donc être dépistée systématiquement ; ensuite, la colonisation à *candida* est un élément dynamique : outre le nombre de sites colonisés (et l'index de colonisation), l'évolution de cette colonisation au cours du temps est un élément majeur. C'est à partir d'une colonisation multiple que se ferait la dissémination vasculaire et tissulaire ; enfin, cet index de colonisation à *candida* doit encore être validé dans une population plus large de malades chirurgicaux, mais aussi chez des malades médicaux.

De façon intéressante, une étude récemment publiée effectuée dans un service de réanimation médicale à Barcelone souligne la relation indépendante entre la colonisation multiple à *candida* et les candidoses invasives (17). Cette étude, avec celle de Jorge Garbino à Genève (31), semble être prometteuse pour la possibilité d'une pertinence clinique de l'index de colonisation en réanimation médicale, mais ceci reste à démontrer dans une étude spécifiquement dessinée à cet effet.

1.3. THÉRAPEUTIQUES ANTIFONGIQUES PRÉVENTIVES CHEZ LES PATIENTS NON NEUTROPÉNIQUES DE RÉANIMATION

Deux stratégies préventives ont été proposées (2). Un schéma descriptif sommaire est rapporté dans la figure 1.

1.3.1. La première stratégie, appelée prophylaxie, concerne des patients de réanimation qui n'ont pas de colonisation à *candida* mais qui présentent des facteurs de risque comme une corticothérapie, une dialyse, des cathéters centraux, une antibiothérapie à large spectre ou encore une ventilation mécanique prolongée. Ce concept de prophylaxie antifongique est emprunté à celui utilisé avec succès chez des malades leucémiques ou greffés de moëlle (32, 33).

1.3.2. La seconde stratégie correspond à l'instauration d'un traitement antifongique chez un patient colonisé en de nombreux sites à *candida*. Il s'agit d'un traitement précoce ou préemptif basé sur la possibilité que cette colonisation multiple soit secondairement le lit d'une candidose invasive.

1.3.3. A ce jour, seules les stratégies prophylactiques ont été évaluées. Globalement, une stratégie prophylactique orale (nystatine ou ketoconazole) ou systémique (fluconazole) permet de décoloniser les patients de réanimation. Cependant, une prophylaxie aura un effet inconstant sur l'incidence de candidose invasive (patient brûlé (34), prématuré (35) ou avec un séjour prolongé en réanimation chirurgicale (36)). Chez l'adulte, une seule étude a permis de démontrer un effet significatif de la prophylaxie sur la mortalité des patients en choc septique lié à une péritonite (37). Par ailleurs, l'ensemble de ces études a aussi démontré la bonne tolérance des molécules antifongiques couramment utilisées en prophylaxie chez les patients de réanimation, élément important pour la mesure du rapport risque / bénéfice.

Figure 1 : stratégies antifongiques préventives proposées

Les stratégies décrites dans ce schéma ne sont pas recommandées à ce jour.

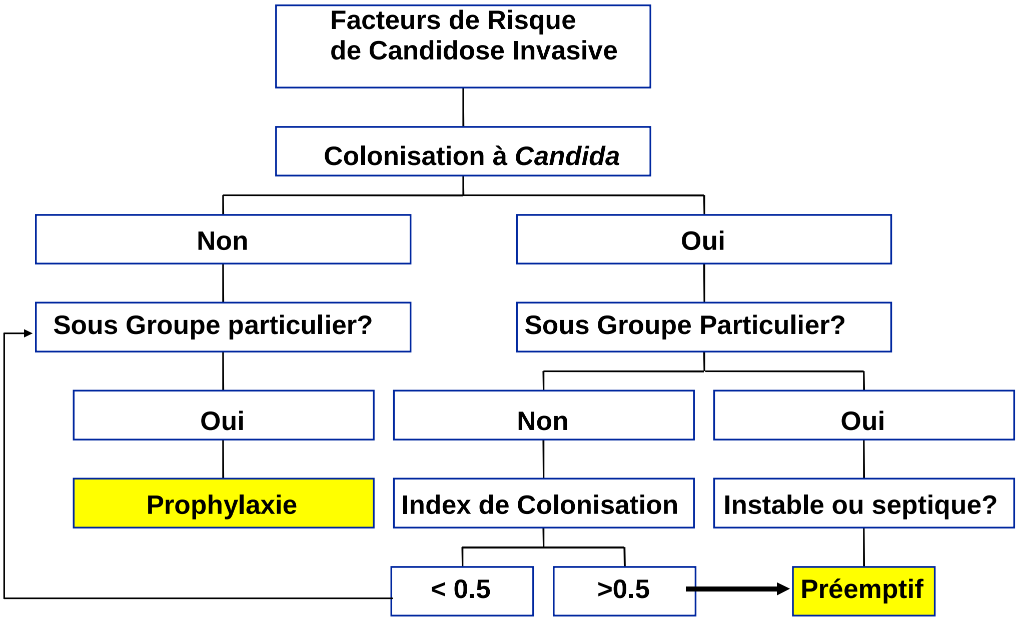

1.4. AMPHOTÉRICINE B LIPOSOMALE : AMBISOME®

L'amphotéricine B est un agent antifongique à large spectre couvrant notamment les infections à *candida spp.* les plus fréquentes (38).

Des nouvelles formulations lipidiques d'amphotéricine B ont été récemment développées et l'une d'elles, l'amphotéricine B liposomale (AmBisome®) a montré au cours de nombreux essais un profil de tolérance supérieur à celui de l'amphotéricine B conventionnelle.

Une large étude comparant AmBisome® (3 mg/kg/j) à l'amphotéricine B (0,6 mg/kg/j) dans le traitement empirique a confirmé la supériorité d'AmBisome® sur le plan de la tolérance notamment au niveau rénal (19% versus 34% de doublement de la créatinine de base, respectivement pour AmBisome® et CAB) (39).

Dans cet essai d'envergure, le taux de succès global était comparable entre les 2 groupes sur un score composite comportant : la survie à 7 jours post-traitement, la résolution de la fièvre pendant la neutropénie, la résolution des signes d'infection fongique, l'absence d'émergence d'IFI et l'arrêt prématuré du traitement. Par contre il a été constaté une incidence des IFI prouvées significativement moindre pour le groupe AmBisome®, différence d'autant plus importante pour les patients allogreffés et pour ceux dont la neutropénie était supérieure ou égale à 7 jours.

Dans le traitement des mycoses systémiques et/ou profondes à *Aspergillus* et *Candida* chez l'adulte et l'enfant, la dose recommandée dans les mentions légales d'AmBisome® est de 3mg/kg/jour. Cependant, les principales infections fongiques (aspergilloses invasives et candidoses invasives) restent associées à une forte mortalité (40, 41) nécessitant des stratégies thérapeutiques innovantes.

Le rationnel de l'administration d'AmBisome® à des doses supérieures à celles de l'AMM s'appuie sur la démonstration de l'effet dose dans des modèles animaux d'infection fongique invasive (J Adler-Moore et Anaissie) et sur l'étude d'escalade de doses conduite par Walsh et al. (42). Des posologies élevées d'AmBisome®, administrées en conditions réelles (patients avec IFI probables ou documentées), pouvaient être utilisées avec un bon profil de tolérance, notamment à la dose de 10 mg/kg/j où la C_{max} et l'AUC ont été maximales. Cette escalade de dose a été poursuivie jusqu'à 15 mg/kg/j sans que la dose maximale tolérée (MTD) soit atteinte chez l'adulte, et jusqu'à 10 mg/kg/j chez l'enfant.

L'étude AmBiload, étude randomisée en double aveugle, sur des critères d'évaluation de l'EORTC modifiés, démontre une efficacité du bras AmBisome® 10mg/kg/j comparable à celle du bras AmBisome® 3mg/kg/j dans le traitement des infections fongiques à filamenteux avec des taux de doublement de créatinine en cours de traitement de l'ordre de 30%, c'est-à-dire identiques à ceux retrouvés dans l'étude d'escalade de dose de Walsh (43). Tous les évènements indésirables liés au produit qui sont survenus étaient connus et documentés ; principalement néphrotoxicité, hypokaliémie, anomalies hépatiques et évènements liés à la perfusion.

L’étude AmBiload a permis de valider l’administration quotidienne d’AmBisome® à dose standard de 3mg/kg/j dans le traitement des infections fongiques invasives à filamenteux et plus spécifiquement dans l’aspergillose invasive avec une efficacité en fin de traitement de 50% et une survie à 12 semaines de 72%.

L’étude Combistrat a confirmé le rationnel de l’administration d’AmBisome® 10mg/kg/j dans le traitement de l’aspergillose invasive avec un taux de survie à 12 semaines de 80%, taux significativement non différent du taux de 100% du bras comparatif associant AmBisome® dose standard à la caspofungine (44). Cette étude constitue un essai pilote, multicentrique, prospectif, randomisé comparant en ouvert AmBisome® 3 mg/kg/j associé à Cancidas® et AmBisome® 10 mg/kg/j, les deux traitements étant maintenus pendant 14 jours, dans le traitement de l’aspergillose invasive probable ou prouvée selon les critères modifiés de l’EORTC-MSG.

Les résultats de cette étude ont mis en évidence une efficacité du bras association en fin de traitement significativement plus élevée que celle du bras haute dose (67% et 27% respectivement, p=0,03) ainsi qu’une survie à 3 mois de 100% dans le bras association versus 80% dans le bras haute dose (non significatif). Sur le plan de la tolérance, ont été rapportés plus d’effets indésirables liés à la perfusion dans le bras monothérapie que dans l’autre bras au 1er jour de traitement.

Ce schéma d’administration d’AmBisome® haute dose pourrait permettre un espacement des administrations jusqu’à une semaine avec des concentrations plasmatiques toujours efficaces. Mehta et al. (45) a publié dans « *Biology of Blood and Marrow Transplantation* » une étude de prophylaxie chez l’enfant [≥ 10 ans] allogreffé avec une administration d’AmBisome® à la dose de 10mg/kg/semaine. Quatorze patients ont été traités, aucun n’avait d’antécédent d’IFI. Seul 1 patient a présenté une infection fongique présumée (sur imagerie avec une biologie négative) qui a été traitée de façon empirique avec succès. Les mesures pharmacocinétiques ont démontré un profil de concentration et de distribution élevé de l’AmBisome® qui reste mesurable au 7ème jour post-perfusion avec une tolérance excellente sans documentation de signes de néphrotoxicité notables, en particulier pas d’élévation significative de la créatinine.

L'étude Prophysome (46), présentée à l'EBMT en 2006 par Catherine Cordonnier, a étudié la tolérance à un schéma thérapeutique prophylactique d'AmBisome® haute dose en administration hebdomadaire chez deux populations à haut risque d'hématologie (allogreffés et leucémies aigues). Cette étude rapporte une très bonne tolérance dans le bras Leucémie Aigue avec aucune intolérance au produit liée à une sortie d'étude prématurée. L'étude a également souligné l'importance d'une bonne concordance entre les dates de perfusions d'AmBisome® et le calendrier hématologique des patients allogreffés en raison d'un conditionnement spécifique.

L'étude AlloAmBi Pro souligne la faisabilité d'un schéma prophylactique par AmBisome® haute dose en perfusion hebdomadaire notamment au décours d'une corticothérapie traitant une maladie du greffon contre l'hôte chez des patients qui bénéficient d'une greffe allogénique non myélo-ablative (47).

1.5. PROBLÉMATIQUE DE L'ÉTUDE

Une étude américaine récente place les infections à *candida spp.* au quatrième rang des infections nosocomiales les plus rencontrées avec un taux de mortalité de 40%. De plus, les infections à *candida spp.* surviennent majoritairement dans les services de réanimation (48, 49). La colonisation à *candida* n'est pas liée à un accroissement de mortalité mais le continuum colonisation – infection à *candida*, démontré en 1994 par Pittet et ses collègues souligne l'importance de la prévention d'une invasion chez les patients à haut risque de candidose invasive (16). De plus, le retour d'une hémoculture positive à *candida* signant une candidémie fréquemment mortelle est un événement tardif et peu sensible.

Ces faits sont en faveur d'un traitement antifongique précoce qui permettrait de traiter efficacement une candidose systémique à une étape où les techniques de diagnostic n'ont pas la sensibilité nécessaire pour affirmer ou infirmer l'infection. Ce traitement serait mené chez des patients hospitalisés en réanimation avec une colonisation par *candida* dans au moins deux sites et à haut risque de candidose invasive.

L'étude AmBidex est une étude prospective ouverte multicentrique dont l'objet est le traitement préemptif des colonisations multiples à *candida* chez des patients de Réanimation présentant un sepsis.

Le caractère dit « préemptif » se définit par l'existence d'une colonisation associée à un contexte de sepsis présumant d'une éventuelle dissémination sans que la candidémie voire la candidose ne puisse être (encore) identifiée. Ce traitement préemptif (et par définition non curatif à ce stade) a pour objectif de prévenir l'émergence d'une candidémie.

A la différence d'un traitement prophylactique, l'initiation d'un traitement préemptif est basée sur la colonisation à candida. Aucune molécule ne fait consensus dans cette indication.

L'administration hebdomadaire d'AmBisome® haute dose est un schéma thérapeutique possible se fondant sur un profil pharmocinétique connu, une plus grande facilité d'administration ainsi que sur un spectre d'activité large.

1.6. OBJECTIFS DE L'ÉTUDE

1.6.1. Objectif principal

L'objectif principal de l'étude est l'évaluation de la sécurité d'emploi et de la tolérance d'AmBisome® administré à raison de 10 mg/kg/semaine chez des patients de réanimation.

1.6.2. Objectifs secondaires

Les objectifs secondaires consistent à évaluer les paramètres suivants :

- ✓ Morbidité selon trois critères :
    - o Durée d'hospitalisation
    - o Durée de séjour en réanimation
    - o Nombre de jours sans défaillance organique
- ✓ Incidence d'infection fongique invasive (critères de l'EORTC-MSG modifiés)
- ✓ Survie à 28 jours

2. MÉTHODOLOGIE DE L'ETUDE

2.1. DESIGN GÉNÉRAL DE L'ÉTUDE

Il s'agit d'une étude pilote, multicentrique, ouverte, prospective, réalisée chez des patients de réanimation colonisés par *candida spp*., à haut risque de candidose invasive.

Les patients seront pré-sélectionnés sur un tableau de défaillance d'organe associé à une ventilation mécanique, à la colonisation d'un site par *candida spp.* en plus du site digestif, ainsi que sur la présence d'un état septique.

L'inclusion fera suite à un changement d'antibiothérapie bactérienne.

Tous les patients seront traités par la formulation liposomale de l'amphotéricine B, AmBisome®, à la dose de 10 mg/kg/semaine. Deux perfusions hebdomadaires du produit à l'étude seront administrées, sauf en cas d'intolérance au traitement ou d'échec de celui−ci, selon l'appréciation de l'investigateur.

Une période de 21 jours fera suite à la seconde perfusion de traitement. Pendant les sept premiers jours de cette période, le patient est considéré, du fait de la longue persistance du traitement dans l'organisme, comme sous traitement ; au huitième jour après la deuxième administration du produit à l'étude, commence la période de suivi de 14 jours. La durée totale d'étude pour un patient est donc de 28 jours.

2.2. SCHÉMA

L'étude suivra le schéma récapitulatif suivant :

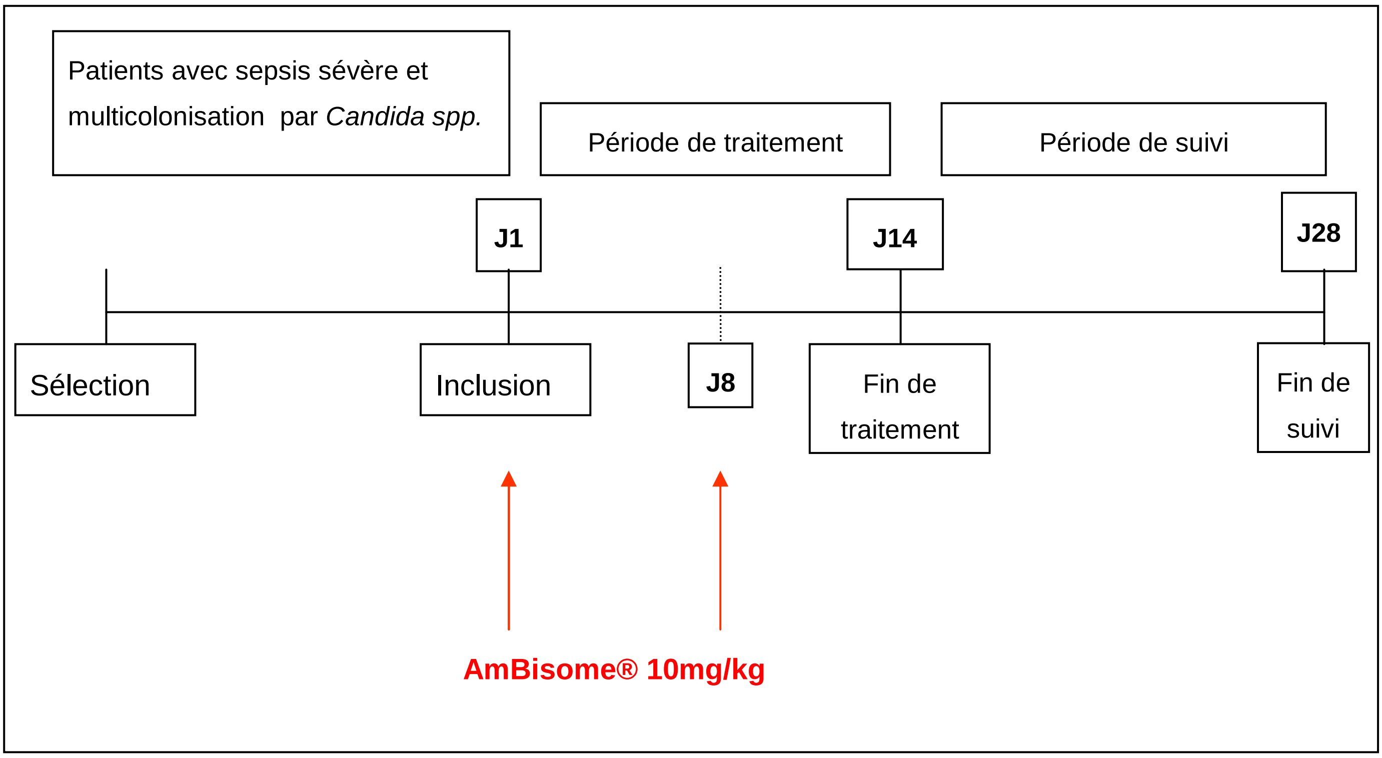

2.3. DISCUSSION SUR LE PLAN EXPÉRIMENTAL

Il s'agit d'une étude de tolérance chez des patients pour lesquels les centres participants n'ont pas de protocole défini de thérapeutique antifongique au stade où le patient est inclus dans l'étude.

Il n'y a donc pas de traitement de comparaison. Un deuxième volet reprenant une population témoin (critères d'inclusions identiques sans administration d'AmBisome® en traitement préemptif) fera l'objet d'une étude rétrospective.

2.4. COMITÉ DE PILOTAGE

Un Comité de Pilotage composé des personnalités suivantes a été mis en place :

- Renée Grillot : mycologue au CHU de Grenoble
- Professeur Elie Azoulay : investigateur coordinateur
- Professeur Michel Wolff : conseiller scientifique, investigateur principal
- Un représentant du Promoteur
- Un représentant de Gilead Sciences qui fournira le produit au Promoteur

Ce Comité est le garant scientifique de la conduite de cette étude. Il veille en particulier à la définition claire des objectifs et à ce que les moyens mis en œuvre permettent de les atteindre. Ce Comité se réunira trois à quatre fois par an pendant l'étude à la demande du Promoteur.

Le plan d'analyse statistique sera soumis au Comité pour approbation finale avant toute analyse. Enfin les résultats seront validés par ce même Comité.

3. POPULATION ÉTUDIÉE

3.1. CRITÈRES D'INCLUSION

- Age ≥ 18 ans
- Ventilation mécanique depuis plus de 48 h
- Patients présentant une défaillance d'organe en plus de celle requérant la ventilation mécanique
- Colonisation à *candida* d'au moins 2 sites dont 1 non digestif
- Suspicion de sepsis nosocomial
- Mise en place d'une nouvelle antibiothérapie pour la suspicion de sepsis nosocomial
- Consentement de la personne de confiance au moment de l'inclusion

3.2. CRITÈRES DE NON INCLUSION

- Traitement par antifongiques par voie orale (décontamination digestive sélective) ou par voie systémique dans les 15 jours précédant l'inclusion
- Patients répondant aux critères de mise en œuvre d'un traitement antifongique classique
- Patients moribonds (SAPS à l'inclusion >65)
- Patients neutropéniques ou receveurs d'une greffe de cellules souches hématopoïétiques ou d'organe solide ou bénéficiant d'une chimiothérapie
- Présence d'une infection fongique documentée (prouvée ou probable) selon les critères de l'EORTC/MSG modifiés
- Créatininémie > 220 µmol/L ou hémodialyse
- Grossesse

4. MODALITÉS DE TRAITEMENT

4.1. PRODUIT ADMINISTRÉ

AmBisome® est administré à la posologie de 10mg/kg/semaine.

4.2. TRAITEMENT À L'ÉTUDE : AMBISOME®

L'ensemble des informations sur le traitement à l'étude est contenu dans les RCP qui figurent en annexe du présent protocole. Les modifications apportées par les Autorités de Santé au RCP ne seront pas considérées comme des modifications substantielles du protocole et ne feront donc pas l'objet d'un amendement mais d'une simple notification administrative.

4.2.1. Formulation

L'amphotéricine B est un antibiotique antifongique de la famille des polyènes macrocycliques, produit par une souche de *Streptomyces nodosus.* Les liposomes sont des vésicules closes, sphériques obtenues en mélangeant des proportions spécifiques de substances amphiphiles, tels que phospholipides et cholestérol, qui vont former des bicouches uniques ou multiples lorsqu'elles se trouvent en solution aqueuse. AmBisome® est constitué de petits liposomes unilamellaires (SUVs : small unilamellar vesicles), d'un diamètre inférieur à 100 nanomètres. L'amphotéricine B est intercalée dans la membrane et stabilisée par un complexe de transfert de charges avec le distéaroylphosphatidylglycérol et par la présence de cholestérol. Le principe actif fait partie intégrante de la structure globale des liposomes d'AmBisome®.

4.2.2. Conditionnement et étiquetage

Poudre en flacon de verre (type I) de 15 mL. La fermeture est constituée par un bouchon en caoutchouc de butyle et sertie par un anneau en aluminium muni d'un opercule en plastique. Les flacons unidoses sont conditionnés dans des boîtes contenant 10 flacons avec 10 filtres stériles de 5 microns pré-conditionnés. Les flacons d'AmBisome® contiennent 50 mg d'amphotéricine B.

Chaque flacon est étiqueté avec les informations requises par la législation :

- Nom et adresse du promoteur
- Référence de l'étude
- Formule du médicament
- Numéro de lot et date d'expiration
- Conditions de stockage
- Instructions de dosage

Et l'indication suivante : « Pour essai clinique uniquement. A n'administrer que sous contrôle médical strict ».

4.2.3. Stockage et manipulation

A conserver à une température comprise entre +2°C et +25°C et à l'abri de la lumière. Ne pas congeler.

4.2.4. Précautions de conservation de la suspension reconstituée avec de l'eau pour préparations injectables

A conserver à une température comprise entre + 2°C et + 8°C pendant 7 jours ou entre + 23°C et + 27°C pendant 24 heures dans le flacon initial ou dans une seringue. Ne pas congeler. NE PAS CONSERVER de flacon partiellement utilisé pour d'autres patients.

4.2.5. Précautions de conservation de la suspension reconstituée avec une solution de glucose à 5%

A conserver à une température comprise entre + 2°C et + 8°C pendant 7 jours ou entre + 23°C et + 27°C pendant 72 heures. Ne pas congeler.

4.2.6. Instructions pour Reconstitution, Filtration et Dilution

AmBisome® doit être reconstitué avec de l'eau pour préparations injectables (ne contenant aucun produit bactériostatique). Les flacons d'AmBisome® contiennent 50 mg d'amphotéricine B.

Reconstitution

1. Ajouter de manière aseptique 12 mL d'eau pour préparations injectables à chaque flacon d'AmBisome® pour obtenir une préparation contenant 4 mg/mL d'amphotéricine B.

   ATTENTION : NE PAS RECONSTITUER LE PRODUIT AVEC DU SÉRUM PHYSIOLOGIQUE, NE PAS AJOUTER DE SÉRUM PHYSIOLOGIQUE AU PRODUIT RECONSTITUÉ, NE PAS MÉLANGER AVEC D'AUTRES PRODUITS.

   L'utilisation d'une solution autre que celles recommandées ou la présence d'un agent bactériostatique (par exemple l'alcool benzylique) dans la solution peut entraîner la précipitation d'AmBisome®.

2. Agiter vigoureusement chaque flacon IMMÉDIATEMENT APRÈS LA DILUTION pendant au moins 15 secondes afin de bien disperser la préparation. La suspension d'AmBisome® est jaune et transparente.

Filtration et dilution

3. Calculer la quantité d'AmBisome® reconstituée (4 mg/mL) devant être ultérieurement diluée pour la perfusion, en fonction de la posologie prescrite. AmBisome® doit être dilué avec une solution injectable de glucose à 5% pour obtenir une concentration finale comprise entre 0,2 mg/mL et 2,0 mg/mL avant administration.

4. Aspirer la quantité d'AmBisome® reconstituée dans une seringue stérile.

5. Attacher le filtre de 5 microns fourni à la seringue. Instiller le contenu de la seringue à travers le filtre dans la quantité appropriée de soluté injectable de glucose à 5%. (Utilisez uniquement un filtre par flacon d'AmBisome®.) Attention : ne pas réutiliser un flacon partiellement utilisé. Comme tous les produits destinés à un usage parentéral, AmBisome® reconstitué devra subir une inspection visuelle pour rechercher les particules de matières ou toute modification de coloration avant d'être administré et ce, chaque fois que la solution et le contenant le permettent. N'utilisez pas le produit s'il y a une faible précipitation ou une quelconque matière étrangère visible.

Il convient de respecter strictement la technique aseptique au cours de la manipulation du produit car aucun conservateur ou agent bactériostatique n'est présent dans AmBisome® ou dans le produit de reconstitution ou celui destiné à diluer le produit. Une membrane filtrante sur le trajet du cathéter peut être utilisée lors de la perfusion intraveineuse d'AmBisome®. Cependant, le diamètre moyen des pores du filtre ne devra pas être inférieur à 1,0 micron.

4.3. DOSAGE ET ADMINISTRATION D'AMBISOME®

AmBisome® doit être administré en perfusion intraveineuse stricte pendant 2 heures sous contrôle médical strict. Pour la première perfusion, les instructions suivantes sont à respecter : pendant les 20 premières minutes d'injection, la vitesse d'administration doit être deux fois plus lente que la vitesse d'administration normale. Le reste de la perfusion sera ensuite administré à une vitesse normale (la première perfusion étant plus longue que les suivantes). Le patient sera particulièrement surveillé pour prévenir tout évènement indésirable.

Une pré-medication prévenant la toxicité liée à la perfusion [antipyrétique (paracétamol uniquement) et antihistaminiques (dexchlorpheniramine uniquement)] peut être administrée à la discrétion de l'investigateur chez les patients ayant déjà présenté des évènements indésirables.

Quand le patient n'a connu aucun épisode de toxicité antérieur, l'utilisation de paracétamol ou dexchlorpheniramine (Polaramine®) est permise en cas de signes ou de symptômes légers.

Si ces signes ou symptômes sont plus sévères, ou s'ils nécessitent un autre traitement, le patient devra sortir de l'étude. En cas d'inconfort persistant après les 30 premières minutes, la perfusion sera administrée sur une durée de 3 heures au lieu de 2.

AmBisome® doit être utilisé exclusivement en perfusion en milieu hospitalier, sous surveillance médicale constante.

Attention : AmBisome® est incompatible avec les solutions de sérum physiologique. Les cathéters initialement placés devront être flushés avec du glucose 5%, ou une autre ligne devra être utilisée.

La perfusion d'Ambisome ne doit pas être passée en Y avec d'autres transfusions, nutrition parentérale, perfusion de macromolécules ou de formulations lipidiques. Il est recommandé

d'espacer la perfusion d'AmBisome d'une autre perfusion (1 à 2 heures) afin de pouvoir évaluer les éventuels effets indésirables.

4.4. DISPONIBILITÉ ET DISPENSATION DES UNITÉS THÉRAPEUTIQUES

Les unités thérapeutiques seront fournies par Gilead Sciences, à titre gratuit, soit, pour 30 patients, un total de 1.000 flacons. Si nécessaire, des quantités supplémentaires pourront être délivrées pour mener l'étude à bonne fin.

Gilead Sciences fournira des flacons à étiquetage commercial. Le Promoteur assurera l'étiquetage essais cliniques ainsi que l'ensemble des responsabilités qui lui incombe dans le cadre législatif.

Toute dispensation d'unités thérapeutiques fera l'objet d'une comptabilité qui sera tenue par la pharmacie hospitalière concernée.

Gilead Sciences remettra au Promoteur toutes les informations relatives aux Unités Thérapeutiques utiles à la réalisation du Protocole et qui seraient en sa possession ou qui viendraient à sa connaissance. Ces informations utiles concernent notamment celles relatives à la durée de stabilité, aux précautions particulières de conservation, aux incompatibilités majeures chimiques ou physiques, aux propriétés pharmacologiques, aux effets indésirables (fréquence et gravité), aux modalités d'administration, aux mises en garde spéciales, aux contre-indications et précautions particulières d'emploi, et aux interactions médicamenteuses.

Gilead Sciences transmettra au fur et à mesure qu'il en aura connaissance, les éléments toxicologiques et cliniques susceptibles de modifier le rapport bénéfice/risque sur l'AmBisome® et en particulier toute modification du procédé de fabrication ou des spécifications survenant au cours de l'étude pour le(s) lot(s) de produit livré(s).

Conformément aux Bonnes Pratiques de Fabrication, Gilead Sciences conservera des échantillons du produit à l'essai qui pourront être mis à la disposition des autorités de santé, pour chacun des lots de produits utilisés dans le cadre de l'étude.

Les produits seront étiquetés et conditionnés pour l'étude, conformément aux dispositions du Code de la Santé Publique (article R.5121-16) et aux dispositions de l'annexe 13 des Bonnes Pratiques de Fabrication Européennes ; ils seront délivrés avec un certificat d'analyses du lot. Les étiquettes comporteront, en particulier, une mention claire de la date de péremption qui doit être postérieure d'au moins 12 mois à la date de livraison.

La distribution des produits aux pharmacies hospitalières (à l'attention du Responsable des Etudes cliniques) sera assurée par le Promoteur au fur et à mesure que des besoins seront exprimés par celles−ci.

Le délai de mise à disposition est de vingt jours.

Le Pharmacien de chacun des centres de l'étude enverra par fax dans les 24 heures au Promoteur l'accusé de réception de la livraison des unités thérapeutiques.

Les unités thérapeutiques utilisées seront comptabilisées au niveau de chaque centre et devront être stockées de manière centralisée à cet effet.

Les unités thérapeutiques non utilisées au cours de l'étude, pour quelque raison que se soit, seront détruites par les soins des pharmacies des différents centres qui enverront au plus tard dans le mois suivant la destruction un certificat au Promoteur comportant les quantités détruites par numéro de lot et date de péremption. Le Promoteur pourra, le cas échéant, réclamer la restitution des unités de traitement afin de procéder à leur destruction. Dans ce cas, le Promoteur prendra en charge tous les coûts relatifs à cette opération.

4.5. TRAITEMENTS CONCOMITANTS

Les traitements concomitants seront reportés dans le cahier d'observation.

Une attention toute particulière sera apportée aux traitements néphrotoxiques concomitants.

Se reporter aux RCP pour les interactions avec d'autres médicaments.

4.6. COMPLIANCE

Le relevé de la compliance est assuré par le personnel soignant qui note les doses administrées et les conditions dans lesquelles ont été réalisées les perfusions.

4.7. PATIENTS EXCLUS EN COURS D'ÉTUDE ET ARRÊT PRÉMATURÉ DE TRAITEMENT

Tous les patients inclus recevant du produit à l'étude, même une seule perfusion incomplète, seront pris en compte dans l'analyse statistique.

En cas d'arrêt prématuré de traitement, le patient sera suivi jusqu'à la fin de l'étude (28 jours après la première perfusion). L'investigateur est seul juge de l'arrêt ou de la modification de dose du produit à l'étude. Toutefois, il devra discuter préalablement avec l'investigateur coordinateur avant de modifier le schéma et la dose d'administration du produit à l'étude.

Si une hémoculture se positive durant l'étude et au terme de celle−ci, l'investigateur est le seul juge du traitement de relais antifongique le plus approprié.

5. VISITES ET PROCÉDURES

Les visites suivantes seront réalisées et permettront de collecter les informations nécessaires :

5.1. V1 (J1) : VISITE D'INCLUSION ET DE PREMIERE PERFUSION

- Recueil du consentement éclairé d'un proche
- Données démographiques
- Vérification des critères d'inclusion et de non inclusion
- Comorbidités et critères de défaillance à l'inclusion
- Signes vitaux avant et après perfusion
- Administration du produit à l'étude (durée, quantité administrée)
- SOFA score
- Critères d'un état septique
- Examens de laboratoire
- Test de grossesse si applicable
- Traitements concomitants
- Evènements indésirables
- Survie

Les facteurs de risque d'infection fongique invasive seront définis à partir de l'index de colonisation corrigé.

Les sites de prélèvements définis pour identifier une éventuelle colonisation sont ceux usuellement prélevés dans les pratiques courantes du service.

Les sites ayant fait l'objet d'un prélèvement à l'inclusion seront évalués durant toute l'étude.

Un recueil des informations sur la colonisation fongique sera fait 2 fois par semaine dans le cahier patient

5.2. V2 (J2) : PREMIÈRE VISITE DE SUIVI DE LA PREMIERE PERFUSION

- Signes vitaux
- SOFA score
- Critères d'un sepsis
- Examen de laboratoire
- Survenue d'infection fongique invasive
- Traitements concomitants
- Evènements indésirables
- Survie

5.3. V3 (J3): DEUXIÈME VISITE DE SUIVI DE LA PREMIERE PERFUSION

- Signes vitaux
- SOFA score
- Critères d'un sepsis
- Examens de laboratoire
- Survenue d'infection fongique invasive
- Traitements concomitants
- Evènements indésirables
- Survie

5.4. V4 (J8) : VISITE DE DEUXIEME PERFUSION

- Administration du produit à l'étude
- Signes vitaux
- SOFA score
- Critères d'un sepsis
- Examens de laboratoire
- Survenue d'infection fongique invasive
- Traitements concomitants
- Evènements indésirables
- Survie

5.5. V5 (J14) VISITE POST TRAITEMENT

- Signes vitaux
- SOFA score
- Critères d'un sepsis
- Examens de laboratoire
- Survenue d'infection fongique invasive
- Traitements concomitants
- Evènements indésirables
- Survie

5.6. V6 (J21) VISITE POST TRAITEMENT

- Signes vitaux
- SOFA score
- Critères d'un sepsis
- Examens de laboratoire
- Survenue d'infection fongique invasive
- Traitements concomitants
- Evènements indésirables
- Survie

5.7. V7 (J28 OU J SORTIE) : VISITE FINALE

- Signes vitaux
- SOFA score
- Critères d'un sepsis
- Examens de laboratoire
- Survenue d'infection fongique invasive
- Traitements concomitants
- Evènements indésirables
- Survie

5.8. Recueil des informations tout au long de l'étude

	V1 (J1)	V2 (J2)	V3 (J3)	V4 (J8)	V5 (J14)	V6 (J21)	V7 (J28 ou J sortie)
Signature du consentement éclairé avant toute procédure liée à l'étude	X						
Démographie	X						
Critères d'inclusion et de non inclusion	X						
Comorbidités à l'inclusion	X						
Signes vitaux	X	X	X	X	X	X	X
SOFA score	X	X	X	X	X	X	X
Critères de sepsis	X	X	X	X	X	X	X
Examens de laboratoire	X	X	X	X	X	X	X
Colonisation à *candida*	X	2 fois par semaine					
Survenue d'infection fongique		X	X	X	X	X	X
Administration du produit à l'étude	X			X			
Traitements concomitants	X	X	X	X	X	X	X
Evénements indésirables	X	X	X	X	X	X	X
Recueil survie	X	X	X	X	X	X	X

6. EVALUATION DE LA TOLÉRANCE

La tolérance au produit à l'étude est évaluée jusqu'à la fin du suivi.

6.1. DÉFINITION

La tolérance au produit à l'étude sera définie au travers des critères suivants :

- Réactions à la perfusion
  - Fièvre
  - Frissons
  - Dyspnée
  - Nausées
  - Vomissements
- Toxicité rénale
  - Doublement de la créatininémie par rapport à la valeur de base
  - Hypokaliémie ≤ 2.5 mmoles
- Autres événements indésirables jugés pertinents par l'investigateur qui feront l'objet d'un recueil exhaustif dans le CRF

6.2. EVÉNEMENTS INDÉSIRABLES

Définitions

Un événement indésirable désigne

Toute manifestation clinique ou biologique, survenue en cours d'étude dès la signature du consentement pour le patient concerné et jusqu'à la fin du suivi du patient, quelle que soit la cause de cette manifestation.

Les évènements indésirables doivent être notifiés par l'investigateur dans le CRF. Une attention particulière sera portée sur les évènements indésirables suivants :

- les évènements indésirables graves
- les hypokaliémies ≤ 2,5 mmoles/l
- les troubles du rythme cardiaque
- les oligoanuries avec diurèse < 400 mL

- les évènements survenant au cours de la perfusion du produit ou dans l'heure qui suit celle−ci
- et tout évènement jugé pertinent par l'investigateur durant les 28 jours de suivi du patient

Après la notification initiale, l'évènement indésirable doit obligatoirement faire l'objet d'un suivi jusqu'à sa résolution. Si l'évènement devient grave, il doit être notifié selon les termes définis si dessous.

Un effet indésirable désigne

Toute manifestation clinique ou biologique, survenue en réaction à l'administration du produit à l'étude, quelle que soit la dose administrée.

Un événement indésirable inattendu désigne

Tout événement indésirable non défini en nature, fréquence et sévérité dans le résumé des caractéristiques du produit (RCP) figurant en annexe du présent protocole.

Un événement indésirable grave (EIG) désigne

Tout événement indésirable qui provoque un décès, une menace vitale immédiate, une incapacité ou une invalidité, temporaire ou permanente, une anomalie congénitale, une hospitalisation ou une prolongation d'hospitalisation.

Conduite à tenir en cas d'évènement indésirable grave

Tout événement indésirable grave ou potentiellement grave doit être déclaré au Promoteur, quelle que soit son imputabilité aux traitements de l'essai à :

Docteur Jean-François DREYFUS

Téléphone mobile : 06 12 82 67 80

Fax : 01 40 86 13 28

Email : jfdreyfus@fovea-group.com

La déclaration se fait par l'un des moyens suivants, au choix de l'investigateur (téléphone suivi d'une confirmation écrite, télécopie, courrier électronique), et comprend au moins les éléments suivants : identification du protocole, du patient, de l'investigateur et de la nature de l'EIG.

L’investigateur doit compléter la rubrique EIG du CRF et doit transmettre dès que possible, mais au plus tard dans les 24 heures, le formulaire de déclaration

Tous les résultats pertinents (examens de laboratoire, examens complémentaires) permettant de mieux caractériser l'effet constaté seront joints au formulaire.

Le rapport initial doit être suivi, le cas échéant, d'un ou plusieurs rapports décrivant l’évolution et l'état résultant ; tout résultat pertinent (examens de laboratoire, examens complémentaires) devra être joint à ces rapports. En cas de décès, s'il y a eu autopsie, le compte−rendu de celle−ci sera transmis au responsable ci−dessus.

Une attention particulière devra être apportée au suivi de l’EIG jusqu’à sa résolution. S'il n'y a pas résolution, le Comité de pilotage de l'étude déterminera le type et la durée du suivi.

Notification des effets indésirables graves par le promoteur

Conformément à la Directive Européenne 2001/20/CE, le responsable de pharmacovigilance du Promoteur transmettra à l’AFSSAPS et au comité de protection des patients concerné les suspicions d’effets indésirables graves inattendus ainsi que tous les décès durant l’étude (au plus tard sous 7 jours calendaires en cas de décès ou sous 15 jours calendaires pour tout autre évènement indésirables grave inattendu). Ces évènements seront également immédiatement introduits dans la base de données Eudravigilance.

L'imputabilité éventuelle à l'AmBisome® et le caractère inattendu ou non de l'évènement seront déterminés en concertation avec l'investigateur.

Le promoteur transmettra une copie de l’EIG au fabricant du produit à l’étude en parallèle de l’envoi aux autorités.

Une fois par an, toutes les suspicions d’effets indésirables graves survenus durant l’étude seront fournies à l’AFSSAPS et au CPP ainsi qu’un rapport concernant la sécurité des participants.

Conduite à tenir en cas d'évènement indésirable lié au produit à l’étude

Si l’investigateur souhaite réduire la posologie du traitement à l’étude ou l’interrompre, il doit en discuter préalablement avec l’investigateur coordinateur.

Tout arrêt de traitement définitif est déclaré immédiatement au promoteur.

Pour tout évènement indésirable lié à la perfusion, l’investigateur doit se conformer au paragraphe « 4.3. Dosage et administration d’AmBisome ». Si malgré ces mesures, l’évènement persiste, en accord avec l’investigateur coordinateur, l’investigateur peut réduire ou interrompre le traitement à l’étude.

Pour tout autre évènement indésirable lié au produit, l’investigateur jugera, après discussion préalable avec l’investigateur coordinateur, de l’administration ou non, complète ou réduite, du produit à l’étude.

Si un patient interrompt l’administration du produit à l’étude, par exemple pour intolérance, l’investigateur devra déployer un effort raisonnable pour qu’il suive les procédures de suivi du protocole. Si ce n’est pas possible, le patient sortira de l’étude. L’investigateur notera les raisons de la sortie d’étude ainsi que la date et remplira un formulaire d’EIG le cas échéant.

6.3. EVALUATION DE L'EFFICACITÉ

6.3.1. Critère principal d'évaluation

La sécurité d'emploi et la tolérance d'AmBisome® chez des patients de réanimation seront évaluées au travers de la fréquence des événements indésirables liés au traitement, survenus durant les 28 jours de l'étude. Les autres données de tolérance (paramètres biologiques, signes vitaux, …) seront prises en compte dans l'analyse lorsque nécessaire.

Les événements indésirables seront présentés sous forme de tables listant ces événements sous forme codée selon le terme préférentiel et la classe-organe (classification MedDRA). Ces tables présenteront le nombre de patients ayant eu un événement indésirable ainsi que la fréquence de survenue de l'événement indésirable. Les événements indésirables seront stratifiés par gravité (grave, non grave), intensité (légère, modérée, sévère), évolution (rétabli, rétabli avec séquelles, en cours, décès), action prise envers le produit à l'étude (aucune, sortie d'étude, augmentation de la durée de perfusion, autre) et le lien avec le produit (non lié, lien potentiel).

6.3.2. Critères secondaires d'évaluation

Les critères secondaires d'évaluation de l'efficacité comprennent :

- L'évaluation des événements indésirables non liés au traitement, survenus durant les 28 jours de l'étude.
- L'évaluation des paramètres de morbidité suivants :
  - Durée de séjour dans l'unité de Soins Intensifs
  - Durée de séjour à l'hôpital
  - Nombre de jours sans défaillance d'organe
- L'incidence des infections fongiques invasives selon les critères de l'EORTC-MSG
- L'incidence d'autres infections nosocomiales subséquentes
- Le taux de mortalité à 28 jours

7. MÉTHODOLOGIE STATISTIQUE

7.1. CALCUL DU NOMBRE DE SUJETS NÉCESSAIRES

Conformément aux ICH E9 se rapportant aux règles statistiques d'un essai clinique, le nombre de sujets prédéfini doit être suffisant pour répondre à l'objectif principal de l'étude, à savoir l'évaluation de la tolérance et de la sécurité d'emploi du produit à l'étude. Par ailleurs, le critère d'évaluation permettant de répondre à cet objectif est purement descriptif avec la présentation exhaustive des évènements indésirables survenant durant le suivi global des patients. Aucun test statistique nécessitant un nombre minimum de patients n'est donc nécessaire à la bonne évaluation du critère principal d'étude.

Compte tenu du nombre de centres participants, de la fréquence d'inclusion et de la durée d'étude envisagée, l'effectif de cette étude pilote non comparative a été fixé à 30 patients.

7.2. EXPLOITATION STATISTIQUE

7.2.1. Populations analysées

Deux populations seront définies :

- Population de tolérance : ensemble des patients qui ont reçu de l'AmBisome® au cours de l'étude, que la dose administrée soit complète ou non.
- Population per protocole : ensemble des patients ayant reçu les deux perfusions prévues d'AmBisome® et ne présentant pas de déviation définie comme majeure par le Comité de Pilotage lors de la Revue des données

7.2.2. Méthodes statistiques

Les données seront analysées par le Promoteur.

L'analyse statistique sera réalisée sur ordinateur PC à l'aide du logiciel SAS (version 8.02 sur Windows PC). Un plan d'analyse statistique sera rédigé avant le gel de la base de données.

* *Expression des résultats*

La distribution des variables quantitatives sera exprimée par la moyenne, l'écart-type, la médiane, le minimum et le maximum.

La distribution des variables qualitatives sera exprimée par la fréquence relative des différentes modalités observées et les effectifs correspondants.

* *Tests statistiques*

La survie à 28 jours sera analysée au moyen de la méthode de Kaplan-Meyer.

S'agissant d'une étude non comparative, aucun autre test statistique ne sera mis en œuvre. Cependant, l'intervalle de confiance à 95% sera calculé pour chacune des fréquences de survenue d'événements indésirables liés au traitement.

7.2.3. Analyse intermédiaire

Il n'est pas prévu d'analyse intermédiaire. Néanmoins, le Comité de Pilotage est fondé à demander une analyse intérimaire s'il juge que la mauvaise tolérance du produit met en péril la santé des patients ou que l'efficacité du traitement est telle qu'il ne serait pas éthique de priver la population générale de ce traitement.

8. RESPONSABITILITÉS DU PROMOTEUR

8.1. CONDUITE DE L'ESSAI

L'étude sera conduite en conformité avec la déclaration d'Helsinki (1964) dans sa dernière version au jour de l'initiation de l'étude (Tokyo, 2004), en accord avec la décision du 24 novembre 2006 relative aux règles de bonnes pratiques cliniques pour les recherches biomédicales portant sur les médicaments à usage humain et de manière plus générale selon la législation et la réglementation en vigueur.

8.2. CONSIDÉRATIONS ÉTHIQUES

Cette étude entre dans le champ d'application du Code de la santé publique Première partie - Livre 1er - Titre II relatif aux Recherches Biomédicales issu de la Loi de santé publique n° 2004-806 du 9 août 2004 et fera donc l'objet d'une déclaration au Comité de Protection des Personnes.

8.3. ORDRE DES MÉDECINS

L'étude donne lieu au versement d'honoraires aux médecins, elle est donc soumise à l'application de l'article L 4113-6 du Code de la santé publique et à ce titre sa mise en œuvre fera l'objet préalablement d'une demande d'avis auprès du Conseil National de l'Ordre des médecins.

8.4. LOI INFORMATIQUE ET LIBERTÉ

En raison du recueil de données médicales indirectement nominatives concernant des patients, cette étude est soumise à l'application de la loi du 1 juillet 1994 relative à leur traitement informatique.

Conformément à sa déclaration à la CNIL, Fovea conduira le traitement des données de la présente étude dans le respect de méthodologie de référence proposée par cette commission en janvier 2006 (MR−001).

Le médecin participant à l'étude informera son patient que des données médicales le concernant feront l'objet d'un traitement informatique à des fins de saisie, d'analyse statistique et d'édition de résultats. A ce titre, son patient dispose d'un droit d'accès ou de rectification sur ces données qui s'exerce à tout moment par l'intermédiaire de son médecin. Une lettre d'information concernant ses droits lui sera remis à l'inclusion par l'investigateur.

8.5. Fourniture des médicaments

Gilead Sciences s'est engagé contractuellement à fournir gratuitement au promoteur de l'étude les produits nécessaires au traitement des patients inclus dans l'étude.

8.6. Conditions de publication

L'étude donnera lieu à la rédaction d'un rapport technique présentant l'ensemble des résultats fournis par l'analyse. La ou les publications seront décidées par le Comité de Pilotage.

8.7. Archivage

Conformément à la législation l'archivage des documents sur site et ceux recueillis par le promoteur portera sur une durée d'au moins 15 ans. Le promoteur sera consulté avant toute destruction de documents relatifs à l'étude et demeurés sur les sites de l'étude.

9. REFERENCES BIBLIOGRAPHIQUES

1. **H. M. Blumberg, W. R. Jarvis, J. M. Soucie, J. E. Edwards, J. E. Patterson, M. A. Pfaller, M. S. Rangel-Frausto, M. G. Rinaldi, L. Saiman, R. T. Wiblin and R. P. Wenzel**
Risk factors for candidal bloodstream infections in surgical intensive care unit patients: the NEMIS prospective multicenter study
The National Epidemiology of Mycosis Survey. Clin Infect Dis. 2001;33(2):177-86

2. **J. H. Rex and J. D. Sobel**
Prophylactic antifungal therapy in the intensive care unit
Clin Infect Dis. 2001;32(8):1191-200

3. **J. Nolla-Salas, A. Sitges-Serra, C. Leon-Gil, J. Martinez-Gonzalez, M. A. Leon-Regidor, P. Ibanez-Lucia and J. M. Torres-Rodriguez**
Candidemia in non-neutropenic critically ill patients: analysis of prognostic factors and assessment of systemic antifungal therapy. Study Group of Fungal Infection in the ICU
Intensive Care Med. 1997;23(1):23-30

4. **G. Leleu, P. Aegerter and B. Guidet**
Systemic candidiasis in intensive care units: A multicenter, matched-cohort study
J Crit Care. 2002;17(3):168-75

5. **M. S. Rangel-Frausto, T. Wiblin, H. M. Blumberg, L. Saiman, J. Patterson, M. Rinaldi, M. Pfaller, J. E. Edwards, Jr., W. Jarvis, J. Dawson and R. P. Wenzel**
National epidemiology of mycoses survey (NEMIS): variations in rates of bloodstream infections due to Candida species in seven surgical intensive care units and six neonatal intensive care units
Clin Infect Dis. 1999;29(2):253-8

6. **W. R. Jarvis**
Epidemiology of nosocomial fungal infections, with emphasis on Candida species
Clin Infect Dis 1995;20(6):1526-30

7. **M. A. Pfaller, R. N. Jones, G. V. Doern, A. C. Fluit, J. Verhoef, H. S. Sader, S. A. Messer, A. Houston, S. Coffman and R. J. Hollis**
International surveillance of blood stream infections due to Candida species in the European SENTRY Program: species distribution and antifungal susceptibility including the investigational triazole and echinocandin agents. SENTRY Participant Group (Europe)
Diagn Microbiol Infect Dis. 1999;35(1):19-25

8. **O. Gudlaugsson, S. Gillespie, K. Lee, J. Vande Berg, J. Hu, S. Messer, L. Herwaldt, M. Pfaller and D. Diekema**
Attributable mortality of nosocomial candidemia, revisited
Clin Infect Dis. 2003;37(9):1172-7. Epub 2003 Oct 8

9. **M. A. Pfaller, D. J. Diekema, R. N. Jones, H. S. Sader, A. C. Fluit, R. J. Hollis and S. A. Messer**
International surveillance of bloodstream infections due to Candida species: frequency of occurrence and in vitro susceptibilities to fluconazole, ravuconazole, and voriconazole of isolates collected from 1997 through 1999 in the SENTRY antimicrobial surveillance program
J Clin Microbiol. 2001;39(9):3254-9

10. **M. A. Pfaller and D. J. Diekema**
Twelve years of fluconazole in clinical practice: global trends in species distribution and fluconazole susceptibility of bloodstream isolates of Candida
Clin Microbiol Infect. 2004;10(Suppl 1):11-23

11. **A. Voss, J. L. le Noble, F. M. Verduyn Lunel, N. A. Foudraine and J. F. Meis**
Candidemia in intensive care unit patients: risk factors for mortality
Infection. 1997;25(1):8-11

12. **A. Voss, J. A. Kluytmans, J. G. Koeleman, L. Spanjaard, C. M. Vandenbroucke-Grauls, H. A. Verbrugh, M. C. Vos, A. Y. Weersink, J. A. Hoogkamp-Korstanje and J. F. Meis**
Occurrence of yeast bloodstream infections between 1987 and 1995 in five Dutch university hospitals
Eur J Clin Microbiol Infect Dis. 1996;15(12):909-12

13. **O. Marchetti, J. Bille, U. Fluckiger, P. Eggimann, C. Ruef, J. Garbino, T. Calandra, M. P. Glauser, M. G. Tauber and D. Pittet**
Epidemiology of candidemia in Swiss tertiary care hospitals: secular trends, 1991-2000
Clin Infect Dis. 2004;38(3):311-20. Epub 2004 Jan 14

14. **J. H. Rex, J. E. Bennett, A. M. Sugar, P. G. Pappas, C. M. van der Horst, J. E. Edwards, R. G. Washburn, W. M. Scheld, A. W. Karchmer, A. P. Dine and al**
A randomized trial comparing fluconazole with amphotericin B for the treatment of candidemia in patients without neutropenia. Candidemia Study Group and the National Institute
N Engl J Med. 1994;331(20):1325-30

15. **M. Leone, J. Albanese, F. Antonini, A. Michel-Nguyen, M. C. Blanc-Bimar and C. Martin**
Long-term epidemiological survey of Candida species: comparison of isolates found in an intensive care unit and in conventional wards
J Hosp Infect. 2003;55(3):169-74

16. **D. Pittet, M. Monod, P. M. Suter, E. Frenk and R. Auckenthaler**
Candida colonization and subsequent infections in critically ill surgical patients
Ann Surg. 1994;220(6):751-8

17. **J. Ibanez-Nolla, M. Nolla-Salas, M. A. Leon, Garci, F. A, J. Marrugat, G. Soria, Di, R. M. Az, R. Torres and J. M. Guez**
Early diagnosis of candidiasis in non-neutropenic critically ill patients
J Infect. 2004;48(2):181-192

18. **T. Calandra, J. Bille, R. Schneider, F. Mosimann and P. Francioli**
Clinical significance of Candida isolated from peritoneum in surgical patients
Lancet. 1989;2(8677):1437-40

19. **S. M. Alden, E. Frank and L. Flancbaum**
Abdominal candidiasis in surgical patients
Am Surg. 1989;55(1):45-9

20. **H. Dupont, C. Paugam-Burtz, C. Muller-Serieys, L. Fierobe, D. Chosidow, J. P. Marmuse, J. Mantz and J. M. Desmonts**
Predictive factors of mortality due to polymicrobial peritonitis with Candida isolation in peritoneal fluid in critically ill patients
Arch Surg. 2002;137(12):1341-6; discussion 1347

21. **H. Dupont, A. Bourichon, C. Paugam-Burtz, J. Mantz and J. M. Desmonts**
Can yeast isolation in peritoneal fluid be predicted in intensive care unit patients with peritonitis?
Crit Care Med. 2003;31(3):752-7

22. **P. Eggimann, P. Francioli, J. Bille, R. Schneider, M. M. Wu, G. Chapuis, R. Chiolero, A. Pannatier, J. Schilling, S. Geroulanos, M. P. Glauser and T. Calandra**
Fluconazole prophylaxis prevents intra-abdominal candidiasis in high-risk surgical patients
Crit Care Med. 1999;27(6):1066-72

23. **M. el-Ebiary, A. Torres, N. Fabregas, J. P. de la Bellacasa, J. Gonzalez, J. Ramirez, D. del Bano, C. Hernandez and M. T. Jimenez de Anta**
Significance of the isolation of Candida species from respiratory samples in critically ill, non-neutropenic patients. An immediate postmortem histologic study
Am J Respir Crit Care Med. 1997;156(2 Pt 1):583-90

24. **J. Rello, M. E. Esandi, E. Diaz, D. Mariscal, M. Gallego and J. Valles**
The role of Candida sp isolated from bronchoscopic samples in nonneutropenic patients
Chest. 1998;114(1):146-9

25. **E. Azoulay and B. Schlemmer**
Candida in Lung Specimens from Non-Neutropenic ICU Patients: Infection or Colonisation?
Yearbook. 2003

26. **T. Lundstrom and J. Sobel**
Nosocomial candiduria: a review
Clin Infect Dis. 2001;32(11):1602-7. Epub 2001 Apr 30

27. **P. Eggimann, J. Garbino and D. Pittet**
Management of Candida species infections in critically ill patients
Lancet Infect Dis. 2003;3(12):772-85

28. **P. Eggimann, J. Garbino and D. Pittet**
Epidemiology of Candida species infections in critically ill non-immunosuppressed patients
Lancet Infect Dis. 2003;3(11):685-702

29. **M. G. Petri, J. Konig, H. P. Moecke, H. J. Gramm, H. Barkow, P. Kujath, R. Dennhart, H. Schafer, N. Meyer, P. Kalmar, P. Thulig, J. Muller and H. Lode**
Epidemiology of invasive mycosis in ICU patients: a prospective multicenter study in 435 non-neutropenic patients. Paul-Ehrlich Society for Chemotherapy, Divisions of Mycology and Pneumonia Research
Intensive Care Med. 1997;23(3):317-25

30. **P. J. Eubanks, C. de Virgilio, S. Klein and F. Bongard**
Candida sepsis in surgical patients
Am J Surg. 1993;166(6):617-9; discussion 619-20

31. **J. Garbino, D. Lew, J.-A. Romand, S. Hugonnet, R. Auckenthaler and D. Pittet**
Prevention of severe Candida infections in non-neutropenic, high-risk, critically ill patients. A randomized, double-blind, placebo-controlled trial in SDD-treated patients
Intensive Care Med. 2002;DOI 10.1007/s00134-002-1540-y

32. **J. L. Goodman, D. J. Winston, R. A. Greenfield, P. H. Chandrasekar, B. Fox, H. Kaizer, R. K. Shadduck, T. C. Shea, P. Stiff, D. J. Friedman and et al**
A controlled trial of fluconazole to prevent fungal infections in patients undergoing bone marrow transplantation
N Engl J Med. 1992;326(13):845-51

33. **D. J. Winston, P. H. Chandrasekar, H. M. Lazarus, J. L. Goodman, J. L. Silber, H. Horowitz, R. K. Shadduck, C. S. Rosenfeld, W. G. Ho, M. Z. Islam and et al**
Fluconazole prophylaxis of fungal infections in patients with acute leukemia. Results of a randomized placebo-controlled, double-blind, multicenter trial
Ann Intern Med. 1993;118(7):495-503

34. **M. H. Desai, R. L. Rutan, J. P. Heggers and D. N. Herndon**
Candida infection with and without nystatin prophylaxis. A 11-year experience with patients with burn injury
Arch Surg. 1992;127(2):159-62

35. **D. Kaufman, R. Boyle, K. C. Hazen, J. T. Patrie, M. Robinson and L. G. Donowitz**
Fluconazole prophylaxis against fungal colonization and infection in preterm infants
N Engl J Med. 2001;345(23):1660-6

36. **R. K. Pelz, C. W. Hendrix, S. M. Swoboda, M. Diener-West, W. G. Merz, J. Hammond and P. A. Lipsett**
Double-blind placebo-controlled trial of fluconazole to prevent candidal infections in critically ill surgical patients
Ann Surg. 2001;233(4):542-8

37. **S. Jacobs, D. A. Price Evans, M. Tariq and N. F. Al Omar**
Fluconazole improves survival in septic shock: a randomized double-blind prospective study
Crit Care Med. 2003;31(7):1938-46

38. **C. Cordonnier**
Fungal infections: current diagnosis and treatment
The Hematology journal 2004; 5:S59-S62

39. **T. Walsh, R. Finberg, C. Arndt and al**
Liposomal amphotericion B for empirical therapy in patients with persistent fever and neutropenia
N Engl J Med 1999; 340: 764-71

40. **J. Mora-Duarte et al**
Comparison of caspofungin and amphotericin b for invasive candidiasis
NEJM 2002; 347 (25) :2020-9

41. **R. Herbrecht, D. W. Denning, T. F. Patterson et al**
Voriconazole versus amphotericin B for primary therapy of invasive aspergillosis
The New England Journal of Medicine 2002 Aug 8;347(6):408-15

42. **T. J. Walsh, J. L. Goodman, P. Pappas et al**
Safety, tolerance, and pharmacokinetics of high-dose liposomal amphotericin B (AmBisome) in patients infected with Aspergillus species and other filamentous fungi: maximum tolerated dose study
Antimicrob Agents Chemother 45:3487–96, 2001

43. **O. A. Cornely, J. Maertens, M. Bresnik et al**
Liposomal amphotericin B as initial therapy for invasive mold infection: a randomized trial comparing a high-loading dose regimen with standard dosing (AmBiLoad trial)
Clin Infect Dis. 2007 May 15;44(10):1289-97

44. **D. Caillot, A. Thiebaut, R. Herbrecht et al**
Liposomal Amphotericin B in Combination with Caspofungin versus Liposomal Amphotericin B High Dose Regimen for the Treatment of Invasive Aspergillosis in Immunocompromised Patients: Randomized Pilot Study (Combistrat Trial)
Focus on Fungal Infections 16 Poster Number 65.00

45. Mehta et al
High-dose weekly AmBisome antifungal prophylaxis in pediatric patients undergoing hematopoietic stem cell transplantation: a pharmacokinetic study
Biol Blood Marrow Transplant. 2006 Feb; 12(2):235-40

46. C. Cordonnier, M. Mohty, C. Faucher et al
Safety of a Weekly Administration of 10 mg/kg of Liposomal Amphotericin B in Antifungal Prophylaxis of Allogeneic Stem-Cell Transplant and Acute Leukemia Patients (Prophysome Trial)
32nd Annual Meeting of the European Group for Blood and Marrow Transplantation Poster Number 703

47. J. El-Cheikh, C. Faucher, S. Furst et al
High-dose weekly liposomal amphotericin B antifungal prophylaxis following reduced-intensity conditioning allogeneic stem cell transplantation
Bone Marrow Transplant. 2007 Mar; 39(5):301-6

48. H. Wisplinghoff, T. Bischoff, S. M. Tallent, H. Seifert, R. P. Wenzel, M. B. Edmond
Nosocomial bloodstream infections in US hospitals: analysis of 24,179 cases from a prospective nationwide surveillance study
Clin Infect Dis. 2004 Aug 1; 39(3):309-17

49. M. B. Edmond, S. E. Wallace, D. K. McClish, M. A. Pfaller, R. N. Jones, R. P. Wenzel
Nosocomial bloodstream infections in United States hospitals: a three-year analysis
Clin Infect Dis. 1999 Aug; 29(2):239-44

1

DENOMINATION DU MEDICAMENT AMBISOME 50 mg, poudre pour suspension de liposomes pour perfusion.

COMPOSITION QUALITATIVE ET QUANTITATIVE

Amphotéricine B 50,00 mg Pour un flacon. Excipients : liposomes (phosphatidylcholine de soja hydrogénée, cholestérol, distéaroylphosphatidylglycérol, sel de sodium, alphatocophérol), saccharose, succinate disodique hexahydraté, hydroxyde de sodium, acide chlorhydrique.

FORME PHARMACEUTIQUE Poudre pour suspension de liposomes pour perfusion.

DONNEES CLINIQUES

Indications thérapeutiques

Traitement des mycoses systémiques et/ou profondes à *Aspergillus* et *Candida* chez l'adulte et l'enfant et des cryptococcoses neuro-méningées chez le sujet infecté par le VIH : ayant développé une insuffisance rénale sous amphotéricine B définie par : · l'élévation de la créatininémie audessus de 220 micromol/l ou l'abaissement de la clairance de la créatinine au-dessous de 25 ml/min. - en cas d'altération pré-existante et persistante de la fonction rénale définie par ·la créatininémie supérieure à 220 micromol/l ou · la clairance de la créatinine inférieure à 25 ml/min.

Traitement empirique des infections fongiques présumées chez des patients neutropéniques fébriles. Le bénéfice maximum a été observé chez les patients greffés de moelle allogénique, les patients adultes avec une neutropénie supérieure ou égale à 7 jours à partir de l'introduction de l'antifongique, recevant en même temps des agents néphrotoxiques.

Traitement des leishmanioses viscérales en cas de résistance prouvée ou probable aux antimoniés.

Posologie et mode d'administration

Posologie Traitement des mycoses systémiques et/ou profondes à *Aspergillus* et *Candida* chez l'adulte et l'enfant. La dose recommandée est de 3mg/kg/jour. Traitement des cryptococcoses neuro-méningées chez le sujet infecté par le VIH. La dose recommandée est de 3 mg/kg/jour. Traitement empirique des infections fongiques présumées chez des patients neutropéniques fébriles. La dose recommandée est de 3 mg/kg/jour. Traitement des leishmanioses viscérales : la dose cumulative recommandée est de 18-24 mg/kg en 6 perfusions : une perfusion par jour pendant 5 jours consécutifs et une 6ème perfusion le 10ème jour chez l'enfant et le sujet

immunocompétent**.** Chez le sujet immunodéprimé, les données sont limitées. Des doses jusqu'à 40mg/kg sur 10 à 20 jours consécutifs ont été administrées. En cas d'aggravation de la fonction rénale sous AMBISOME, la poursuite du traitement doit être discutée en fonction du rapport bénéfice/risque estimé. En l'absence de schéma validé d'adaptation posologique, il est proposé d'abaisser les doses d'AMBISOME à 1,5 mg/kg/jour de manière transitoire, ou d'espacer temporairement les perfusions. *Enfants* : les études cliniques montrent qu'en pédiatrie les doses sont identiques à celles utilisées chez l'adulte, rapportées au kilo de poids corporel. *Sujets âgés* : aucune donnée chez le sujet âgé (≥ 65 ans) ne permet de recommander un schéma posologique différent de celui de l'adulte.

Mode d'administration

AMBISOME doit être administré en perfusion intraveineuse stricte de 30 à 60 minutes. Si le patient ressent une gêne au cours de cette perfusion, la durée de la perfusion peut être prolongée. La concentration recommandée pour la perfusion intraveineuse est comprise

entre 0,2 mg/ml et 2,0 mg/ml d'AMBISOME. **Contre-indications** AMBISOME est contreindiqué chez les patients présentant une hypersensibilité connue à l'amphotéricine B ou à tout autre constituant.

Mises en garde spéciales et précautions particulières d'emploi Ce médicament est soumis à prescription hospitalière. Les premières doses, jusqu'à l'équilibration du traitement, sont administrées sous surveillance médicale afin de vérifier l'absence d'hypersensibilité immédiate et la posologie optimale. De rares réactions anaphylactoïdes ont été rapportées lors de perfusion d'AMBISOME. En cas de survenue d'une réaction anaphylactoïde, la perfusion doit être immédiatement arrêtée et le patient ne devra plus recevoir d'autre perfusion d'AMBISOME. Des réactions sévères liées à la perfusion peuvent survenir au cours d'administration de produits contenant de l'amphotéricine B, y compris AMBISOME. Ces manifestations peuvent être réduites par une vitesse de perfusion plus lente, l'administration d'antihistaminiques, d'antiémétiques, d'antipyrétiques ou de corticoïdes. Des réactions indésirables peuvent apparaître lors de l'utilisation d'AMBISOME, il conviendra d'être prudent en cas de traitement prolongé. Une évaluation biologique régulière de la fonction rénale, hépatique et hématopoïétique doit être réalisée. De plus, l'équilibre électrolytique, en particulier le potassium et le magnésium, doit également être évalué. En cas d'altération cliniquement significative de la fonction rénale ou d'aggravation d'autres paramètres, il conviendra de diminuer la posologie ou d'interrompre le traitement sur la base des résultats des examens biologiques.

Chez le diabétique :

AMBISOME contient environ 900 mg de saccharose par flacon. AMBISOME peut se révéler néphrotoxique, l'aggravation significative de la fonction rénale au cours du traitement par AMBISOME doit conduire à diminuer la posologie ou à interrompre le traitement jusqu'au retour à l'état antérieur ; cette décision doit toutefois tenir compte des éventuels autres traitements pris simultanément par le patient et qui pourraient eux-mêmes être néphrotoxiques.

Hémodialyse chronique : l'administration d'AMBISOME ne devra être commencée qu'après la fin de l'hémodialyse. Il n'est pas nécessaire d'adapter la posologie chez des patients sous hémofiltration. Ce médicament est généralement déconseillé avec le sultopride (cf. rubrique interactions avec d'autres médicaments ou autres formes d'interactions).

Interactions avec d'autres médicaments et autres formes d'interactions

Au cours d'une étude clinique, l'amphotéricine B liposomale s'est révélée significativement moins néphrotoxique que l'amphotéricine B conventionnelle chez le sujet ayant reçu une greffe de moelle en cas d'association à la ciclosporine et/ou certains aminosides (gentamycine, tobramycine, amikacine). HYPOKALIEMIANTS L'hypokaliémie est un facteur favorisant l'apparition de troubles du rythme cardiaque (torsades de pointe, notamment) et augmentant la toxicité de certains médicaments, par exemple la digoxine. De ce fait, les médicaments qui peuvent entraîner une hypokaliémie sont impliqués dans un grand nombre d'interactions. Il s'agit des diurétiques hypokaliémiants, seuls ou associés, des laxatifs stimulants, des glucocorticoïdes, du tétracosactide et de l'amphotéricine B (voie IV).

MÉDICAMENTS NÉPHROTOXIQUES L'utilisation conjointe de médicaments ayant une toxicité rénale propre augmente le risque de néphrotoxicité. Si une telle association est nécessaire, il faut renforcer la surveillance biologique rénale. Les médicaments concernés sont représentés notamment par les produits de contraste iodés, les aminosides, les organoplatines, le méthotrexate à fortes doses, certains antiviraux tels que la pentamidine, le foscarnet, les "ciclovirs", la ciclosporine ou le tacrolimus. Association déconseillée (voir

rubrique Mises en garde spéciales et précautions particulières d'emploi) + Sultopride : risque majoré de troubles du rythme ventriculaire, notamment de torsades de pointe.
Associations faisant l'objet de précautions d'emploi
Autres hypokaliémiants Diurétiques hypokaliémiants (seuls ou associés), laxatifs stimulants, gluco et minéralocorticoïdes (voie générale), tétracosactide : risque majoré d'hypokaliémie. Surveillance de la kaliémie et si besoin correction, notamment en cas de traitement digitalique. + *Digitaliques* : hypokaliémie favorisant les effets toxiques des digitaliques. Corriger auparavant toute hypokaliémie et réaliser une surveillance clinique, électrolytique et électrocardiographique
Médicaments susceptibles de donner des torsades de pointe (sauf sultopride) : antiarythmiques de classe Ia (quinidine, hydroquinidine, disopyramide) et de classe III (amiodarone, sotalol, dofétilide, ibutilide), certains neuroleptiques (thioridazine, chlorpromazine, lévomépromazine, trifluopérazine, cyamémazine, amisulpride, tiapride, sulpiride, halopéridol, dropéridol, pimozide), bépridil, cisapride, diphémanil, érythromycine IV, halofantrine, luméfantrine, méthadone, mizolastine, moxifloxacine , pentamidine, spiramycine IV, véralipride, vincamine IV… : risque majoré de troubles du rythme ventriculaire, notamment de torsades de pointe.
Corriger toute hypokaliémie avant d'administrer le produit et réaliser une surveillance clinique, électrolytique et électrocardiographique.
Zidovudine : augmentation de la toxicité hématologique (addition d'effets de toxicité médullaire). Contrôle plus fréquent de l'hémogramme.
Associations à prendre en compte
Aminosides : risque accru de néphrotoxicité.
Ciclosporine, tacrolimus : augmentation de la créatinémie plus importante que sous l'immunodépresseur seul (synergie des effets néphrotoxiques des deux substances).

Grossesse et allaitement

Grossesse Les études chez l'animal n'ont pas mis en évidence d'effet tératogène. En l'absence d'effet tératogène chez l'animal, un effet malformatif dans l'espèce humaine n'est pas attendu. En effet, à ce jour, les substances responsables de malformations dans l'espèce humaine se sont révélées tératogènes chez l'animal au cours d'études bien conduites sur deux espèces. En clinique, il n'existe pas actuellement de données suffisamment pertinentes pour évaluer un éventuel effet malformatif ou foetotoxique de l'amphotéricine B liposomale lorsqu'elle est administrée pendant la grossesse. L'utilisation de l'amphotéricine B liposomale ne doit être envisagée au cours de la grossesse que si nécessaire. En cas d'administration en fin de grossesse, tenir compte de la toxicité rénale éventuelle de ce médicament pour le nouveau-né.
Allaitement En l'absence de données sur le passage dans le lait de ce médicament, l'allaitement est déconseillé.

Effets sur l'aptitude à conduire des véhicules et à utiliser des machines

Les effets d'AMBISOME sur la capacité à conduire des véhicules et à utiliser des machines n'ont pas été étudiés. Certains des effets indésirables d'AMBISOME cités ci-après risquent d'altérer l'aptitude à conduire des véhicules ou à utiliser des machines.

Effets indésirables

La fièvre et les frissons sont les réactions liées à la perfusion les plus fréquentes susceptibles de survenir lors de l'administration de la première dose d'AMBISOME si aucune prémédication n'est administrée pour prévenir ces réactions. Les autres réactions possibles liées à la perfusion, moins fréquentes, peuvent comporter un ou plusieurs des symptômes suivants :

dorsalgies et/ou oppression ou douleurs thoraciques, dyspnée, bronchospasme, bouffées vasomotrices, tachycardie, hypotension artérielle ; ces réactions disparaissent rapidement à l'arrêt de la perfusion. Elles ne réapparaissent généralement pas lors des administrations ultérieures ou lorsque la vitesse de perfusion est ralentie (sur 2 heures) (voir rubrique « Mise en garde spéciales et précautions particulières d'emploi » en ce qui concerne la prévention et le traitement de ces réactions). Pour les réactions indésirables suivantes l'incidence a été évaluée, elle est basée sur l'analyse des données regroupées d'essais cliniques conduits chez 688 patients traités par AMBISOME.
Très fréquents : fièvre, frissons, hypokaliémie, nausées, vomissements.
Fréquents : troubles rénaux : élévation de la créatininémie, de l'azotémie, hypomagnésémie, hypocalcémie, hyponatrémie, troubles hépatiques : élévation des phosphatases alcalines, bilirubinémie, perturbation du bilan hépatique, diarrhée, céphalées, dorsalgies, douleurs thoraciques, douleurs abdominales, dyspnée, bouffées vasomotrices/vasodilatation, tachycardie, hypotension artérielle, éruption, hyperglycémie.
Peu fréquents : convulsions, bronchospasme, thrombocytopénie, réactions anaphylactoïdes. En plus des rares cas de réactions anaphylactoïdes signalés après la commercialisation du produit, de très rares cas d'oedème de Quincke ont été rapportés chez des patients traités par perfusion d'AMBISOME. Les effets suivants ont été rapportés lors de l'utilisation d'amphotéricine B mais peuvent éventuellement survenir avec AMBISOME : généraux : fièvre, frissons, malaise, perte de poids, flush, rénaux : altération de la fonction rénale : hypokaliémie, azotémie, augmentation de la créatinine sérique, hypomagnésémie, hypocalcémie, hyponatrémie, acidose tubulaire distale, insuffisance rénale si administration de doses totales importantes, gastro-intestinaux : nausées, vomissements, diarrhées, anorexie, dyspepsie, douleurs gastriques, métaboliques : hyperglycémie, hépatiques : élévation des phosphatases alcalines, de la bilirubine, perturbation du bilan hépatique, pulmonaires : dyspnée, bronchospasme, oedème pulmonaire non cardiogénique, neurologiques : céphalées, convulsions, perte d'audition, bourdonnements d'oreilles, troubles de la vision ou diplopie, vertiges, neuropathies périphériques, locomoteurs : dorsalgies avec ou sans oppression de la cage thoracique, douleurs thoraciques, douleurs abdominales, douleurs musculaires et articulaires, cardiovasculaires : bouffées vasomotrices/vasodilatation, tachycardie, hypotension artérielle, arrêt cardiaque, hypertension, choc, allergiques : éruption, réactions anaphylactiques, oedème de Quincke, hématologiques : anémie normochrome normocytaire, agranulocytose, thrombocytopénie, leucopénie, éosinophilie, locaux : douleurs au point d'injection avec ou sans phlébite ou thrombophlébite, dermatologiques : rash en particulier maculo-papuleux, prurit.

Surdosage

La toxicité d'AMBISOME en cas de surdosage n'a pas été établie. Des doses quotidiennes répétées atteignant 10 mg/kg chez des enfants et 15 mg/kg chez des adultes ont été administrées lors des essais cliniques sans qu'il soit rapporté de toxicité dose-dépendante.
Un surdosage en amphotéricine B peut provoquer un arrêt cardiocirculatoire chez l'enfant. En cas de surdosage, il faut arrêter immédiatement l'administration du produit. Il faut également surveiller attentivement l'état clinique du patient, notamment sa fonction rénale et hépatique, l'équilibre électrolytique et le bilan hématologique. L'élimination d'AMBISOME ne semble pas être influencée ni par l'hémodialyse ni par la dialyse péritonéale.

PROPRIETES PHARMACOLOGIQUES

Propriétés pharmacodynamiques ANTIMYCOSIQUE A USAGE SYSTEMIQUE, code ATC : J02AA01 L'amphotéricine B est un antibiotique antifongique de la famille des polyènes macrocycliques, produit par une souche de *Streptomyces nodosus.* Les liposomes sont des vésicules closes, sphériques obtenues en mélangeant des proportions spécifiques de substances amphiphiles, tels que phospholipides et cholestérol, qui vont former des bicouches uniques ou multiples lorsqu'elles se trouvent en solution aqueuse. AMBISOME est constitué de petits liposomes unilamellaires (SUVs : small unilamellar vesicles), d'un diamètre inférieur à 100 nm. L'amphotéricine B est intercalée dans la membrane, et stabilisée par un complexe de transfert de charges avec le distéaroylphosphatidylglycérol et par la présence de cholestérol. Le principe actif fait partie intégrante de la structure globale des liposomes d'AMBISOME. Le mécanisme d'action spécifique (ciblage de l'infection fongique) d'AMBISOME est dû à plusieurs facteurs aboutissant à une concentration fongicide d'amphotéricine B délivrée au niveau de la membrane cellulaire du champignon. L'amphotéricine B est fermement insérée dans la bicouche des liposomes. AMBISOME, de par sa stabilité, reste intact dans la circulation à fortes concentrations pendant des périodes de temps prolongées. Ceci entraîne une distribution tissulaire élevée comparativement à celle de l'amphotéricine B non liposomale. AMBISOME interagit directement avec les champignons en exerçant un effet fongicide renforcé. Les liposomes adhèrent à la paroi cellulaire du champignon, site de l'interaction locale avec AMBISOME. Cette interaction rompt le liposome et l'amphotéricine B est libérée et endommage la membrane cellulaire du champignon, entraînant sa mort. Activité microbiologique : l'amphotéricine B a démontré une activité contre les germes responsables des mycoses systémiques suivantes : *Candida albicans, Rhodotorula, Aspergillus fumigatus, Cryptococcus neoformans, Mucor mucedo, Absidia, Rhizopus, Histoplasma capsulatum, Blastomyces dermatitidis, Sporothrix schenckii* dans la sporotrichose disséminée, *Coccidioïdes immitis.* L'amphotéricine B a démontré une activité faible ou relative (variable) contre les germes responsables des mycoses systémiques suivantes : *Candida non albicans*, en particulier *C. parapsilosis*, *Aspergillus fumigatus* dans l'aspergillome, *Conidiobolus*, *Sporothrix schenckii* dans la sporotrichose lymphocutanée, *Coccidioïdes immitis* dans les atteintes pulmonaires diffuses. L'activité *in vitro* d'AMBISOME sur les souches fongiques pathogènes est comparable à celle de l'amphotéricine B. Toutefois, l'activité *in vitro* d'AMBISOME n'est pas prédictive de son efficacité sur la cellule d'hôte infectée.

Propriétés pharmacocinétiques Le profil pharmacocinétique d'AMBISOME a été évalué chez des patients atteints de neutropénie fébrile ou ayant eu une transplantation de moelle osseuse, qui ont reçu des perfusions d'1 à 2 heures de 1,0 à 7,5 mg/kg/jour d'AMBISOME pendant 3 à 20 jours. Les paramètres pharmacocinétiques d'AMBISOME (moyenne + écart-type) après la première et la dernière administration varient de : Cmax : 7,3 µg/ml (+ 3,8) à 83,7 µg/ml (+ 43,0) - T ½ : 6,3 heures (+ 2,0) à 10,7 heures (+ 6,4) - AUC_{0-24} : 27 µg.h/ml (+ 14) à 555 µg.h/ml (+ 311) - Clairance : 11 ml/h/kg (+ 6) à 51 ml/h/kg (+ 44) - Volume de distribution : 0,10 l/kg (+ 0,07) à 0,44 l/kg (+ 0,27) Les valeurs pharmacocinétiques minimales et maximales ne résultent pas nécessairement des doses respectivement les plus faibles et les plus fortes. Les concentrations sériques et AUC sont plus élevées avec AMBISOME qu'avec l'amphotéricine B classique. L'AUC_{0-24} moyenne à l'équilibre n'est pas dose-dépendante. Elle augmente de façon plus importante que les doses. La clairance moyenne à l'équilibre est indépendante de la dose. Après la dernière perfusion, l'amphotéricine B reste décelable au-delà d'un mois chez quelques patients. La distribution hors du compartiment plasmatique est très importante. Les concentrations moyennes minimales d'amphotéricine B, bien que variables, restent relativement

constantes lors d'administrations réitérées d'une même dose comprise entre 1,0 et 7,5 mg/kg/jour.
L'accumulation d'AMBISOME dans les tissus n'a pas été étudiée. Les données relatives à la diffusion tissulaire montrent que le produit est concentré dans le système réticulo-endothélial.
Considérant les faibles concentrations sériques résiduelles d'AMBISOME après arrêt du traitement, le risque potentiel d'effet indésirable au niveau des tissus n'est pas éliminé.
Les voies métaboliques de l'amphotéricine B non liposomale et d'AMBISOME ne sont pas connues. Les voies d'élimination d'AMBISOME n'ont pas été étudiées. La demi-vie d'élimination est d'environ 7 heures après administration répétée. En raison de la taille des liposomes, il n'y a pas de filtration glomérulaire et d'élimination rénale. L'effet de l'insuffisance rénale ou hépatique sur la pharmacocinétique d'AMBISOME n'a pas été spécifiquement étudié.
Données de sécurité précliniques Lors des études de toxicité sub-chronique effectuées par voie IV chez le chien (1 mois), le lapin (1 mois) et le rat (3 mois), les doses sans effets toxiques se sont révélées inférieures aux doses thérapeutiques. Les organes cibles ont été le foie et le rein qui sont ceux connus pour la toxicité de l'amphotéricine B.
AMBISOME s'est révélé dénué de potentialité mutagène ou clastogène. Il n'a pas été conduit d'étude de carcinogénicité avec AMBISOME. Aucun effet indésirable n'a été observé sur les fonctions de reproduction des rats mâles et des rats femelles. Voir également rubrique « Grossesse et allaitement ».
DONNEES PHARMACEUTIQUES
Incompatibilités Ne pas reconstituer le produit avec du sérum physiologique, ne pas ajouter de sérum physiologique au produit reconstitué, ne pas mélanger avec d'autres produits. L'utilisation de toute autre solution que celles recommandées ou d'agent bactériostatique (comme l'alcool benzylique) dans la solution, peut entraîner une précipitation d'AMBISOME. Tous les dispositifs d'administration intraveineuse doivent être lavés avec une solution injectable de glucose à 5 % préalablement à la perfusion d'AMBISOME. En cas d'impossibilité, AMBISOME devra être administré par une autre voie.
Durée de conservation 3 ans Durée de conservation de la suspension reconstituée avec de l'eau pour préparations injectables : Les données de stabilité physico-chimiques d'AMBISOME reconstitué avec de l'eau pour préparations injectables ont été démontrées dans le flacon initial pendant 24 heures à une température comprise entre + 23℃ et + 27℃ exposé à la lumière et pendant 7 jours exposé à une température comprise entre + 2℃ et + 8℃ dans le flacon initial ou dans une ser ingue en polyéthylène. AMBISOME est fourni en flacon à usage unique. NE PAS CONSERVER de flacon partiellement utilisé pour d'autres patients si la solution n'est pas utilisée immédiatement. Comme AMBISOME ne contient pas d'agent bactériostatique, d'un point de vue microbiologique, la suspension reconstituée et diluée doit être utilisée immédiatement. Si la solution n'est pas utilisée immédiatement, la durée et les conditions de stockage avant administration sont de la responsabilité de l'utilisateur. AMBISOME ne doit normalement pas être conservé plus de 24 heures à une température comprise entre + 2℃ et + 8℃, à moins que la reconstitution ait été réalisée dans des conditions aseptiques contrôlées et validées.
Durée de conservation de la suspension reconstituée avec une solution de glucose à 5% : Les données de stabilité physico-chimiques d'AMBISOME reconstitué avec de l'eau pour préparations injectables puis dilué entre 0,2 mg/ml et 2,0 mg/ml avec une solution de glucose à 5% ont été démontrées pour des températures comprises entre + 23℃ et + 27℃ pendant 72 heures et pour des températures c omprises entre + 2℃ et + 8℃

pendant 7 jours si la solution n'est pas utilisée immédiatement. Comme AMBISOME ne contient pas d'agent bactériostatique, d'un point de vue microbiologique, la suspension reconstituée et diluée doit être utilisée immédiatement. Si la solution n'est pas utilisée immédiatement, la durée et les conditions de stockage avant administration sont de la responsabilité de l'utilisateur. AMBISOME ne doit normalement pas être conservé plus de 24 heures à une température comprise entre + 2℃ et + 8℃, à moins que la reconstitution ait été réalisée dans des conditions aseptiques contrôlées et validées.
Précautions particulières de conservation A conserver à une température comprise entre + 2℃ et + 25℃. Ne pas congeler.
Précautions de conservation de la suspension reconstituée avec de l'eau pour préparations injectables : A conserver à une température comprise entre + 2°C et + 8℃ pendant 7 jours ou entre + 23℃ et + 27℃ pendant 2 4 heures dans le flacon initial ou dans une seringue. Ne pas congeler. NE PAS CONSERVER de flacon partiellement utilisé pour d'autres patients.
Précautions de conservation de la suspension reconstituée avec une solution de glucose à 5% : A conserver à une température comprise entre + 2°C et + 8℃ pendant 7 jours ou entre + 23℃ et + 27℃ pendant 72 heures. Ne pas co ngeler.
Nature et contenu de l'emballage extérieur Poudre en flacon de verre (type I) de 15 et 30 ml. La fermeture est constituée par un bouchon en caoutchouc de butyle et sertie par un anneau en aluminium muni d'un opercule en plastique. Les flacons unidoses sont conditionnés dans des boîtes contenant 10 flacons avec 10 filtres stériles de 5 microns pré-conditionnés.
Instructions pour l'utilisation, la manipulation et l'élimination Avant toute reconstitution, lire attentivement la totalité de ce paragraphe. AMBISOME doit être reconstitué avec de l'eau pour préparations injectables (ne contenant aucun produit bactériostatique). Les flacons d'AMBISOME contiennent 50 mg d'amphotéricine B.
Reconstitution
1. Ajouter de manière aseptique 12 ml d'eau pour préparations injectables à chaque flacon d'AMBISOME pour obtenir une préparation contenant 4 mg/ml d'amphotéricine B. Attention : ne pas reconstituer le produit avec du sérum physiologique, ne pas ajouter de sérum physiologique au produit reconstitué, ne pas mélanger avec d'autres produits. L'utilisation de l'une quelconque des solutions autres que celles recommandées ou la présence d'un agent bactériostatique (par exemple l'alcool benzylique) dans la solution peut entraîner la précipitation d'AMBISOME.
2. Agiter vigoureusement chaque flacon immédiatement après la dilution pendant au moins 15 secondes afin de bien disperser la préparation. La suspension d'AMBISOME est jaune et transparente. Filtration
3. Calculer la quantité d'AMBISOME reconstituée (4 mg/ml) devant être ultérieurement diluée pour la perfusion, en fonction de la posologie prescrite. AMBISOME doit être dilué avec une solution injectable de glucose à 5 % pour obtenir une concentration finale comprise entre 0,2 mg/mL et 2,0 mg/mL avant administration.
4. Aspirer la quantité d'AMBISOME reconstituée dans une seringue stérile.
5. Attacher le filtre de 5 microns fourni à la seringue. Instiller le contenu de la seringue à travers le filtre dans la quantité appropriée de soluté injectable de glucose à 5 %. (Utilisez uniquement un filtre par flacon d'AMBISOME.) Attention : jetez tous les flacons partiellement utilisés. Comme tous les produits destinés à un usage parentéral, AMBISOME reconstitué devra subir une inspection visuelle pour rechercher les particules de matières ou toute modification de coloration avant d'être administré et ce chaque fois que la solution et le contenant le permettent. N'utilisez pas le produit s'il y a une faible

précipitation ou une quelconque matière étrangère visible. Il convient de respecter strictement la technique aseptique au cours de la manipulation du produit car aucun conservateur ou agent bactériostatique n'est présent dans AMBISOME ou dans le produit de reconstitution ou celui destiné à diluer le produit . Une membrane filtrante sur le trajet du cathéter peut être utilisée lors de la perfusion intraveineuse d'AMBISOME. Cependant, le diamètre moyen des pores du filtre ne devra pas être inférieur à 1,0 micron.
TITULAIRE DE L'AUTORISATION DE MISE SUR LE MARCHE GILEAD SCIENCES 100, avenue de Suffren 75015 Paris
PRESENTATION ET NUMERO D'IDENTIFICATION ADMINISTRATIVE 561571-7 Poudre en flacon (verre) de 30 ml avec filtre ; boîte de 10. 562408-2 Poudre en flacon (verre) de 15 ml avec filtre ; boîte de 10.
CONDITIONS DE PRESCRIPTION ET DE DELIVRANCE Liste I.
Médicament soumis à prescription hospitalière. Inscription sur la liste rétrocession (06/09/2005) et sur la liste T2A (31/12/2004)
TITULAIRE DE L'AUTORISATION DE MISE SUR LE MARCHE GILEAD Sciences, 100, avenue de Suffren - 75015 Paris - Tel. : 01.42.73.70.70
DATE D'APPROBATION/REVISION 20 octobre 2006 - Agréé aux collectivités
