# Supplementary material for: Weekly high-dose liposomal amphotericin B (L-AmB) in critically ill septic patients with multiple Candida colonization: The AmBiDex study
Source: PLoS One. 2017 May 22;12(5):e0177093. doi: 10.1371/journal.pone.0177093 (PMC5439673; doi:10.1371/journal.pone.0177093)
Supplement: S2 File — (PDF) [file pone.0177093.s002.pdf]

FOVEA

RECHERCHE CLINIQUE

Comité de Protection des Personnes Ile de France VI
10, Pavillon Jacquart 3ème étage
47 Boulevard de l'Hôpital
75651 PARIS CEDEX 13

Rueil Malmaison, le 5 mai 2008

Objet ***: Courrier de demande de modification substantielle de la liste des investigateurs pour l'essai «*Traitement préemptif des colonisations multiples à candida chez des patients de réanimation présentant »**

Monsieur le Président,

Dans le cadre de la recherche ci-dessus référencée, nous soumettons à votre avis une demande substantielle portant sur un ajout de nouveaux investigateurs.

Vous trouverez joint à ce courrier :

- Formulaire de demande de modifications substantielle FAMS 280806
- CV et liste des nouveaux investigateurs
- Liste complète des investigateurs de l'étude mise à jour au 5 mai 2008.

Je vous prie d'agréer, Monsieur le Président, mes salutations distinguées.

Dr Jean François DREYFUS
Chef de Projet Clinique

NF EN ISO 9001
DNV cofrac CERTIFICATION D'ENTREPRISES & DE PERSONNES
N°0007-2001-FR-COFRAC-QUA

3 bis, Chemin de la Jonchère - 92500 Rueil-Malmaison
Tél. : +33 1 47 14 04 95 - Fax : +33 1 47 16 11 10 - Site internet : www.fovea-group.com
R.C. Nanterre B 347 758 336 - N° Intracommunautaire FR 56 347 758 336 - SA au capital de 150 000 euros - APE 741 E

Liste des nouveaux investigateurs AmBiDex Mai 08

N° centre	Investigateur	Fonction	Adresse 1	Adresse 2	CP - VILLE	Déclaration initial	CV joint
1	Dr WILLENS	Investigateur					X
02	Dr Montravers	Investigateur					X
03	Dr Boussekey	Investigateur					X
	Dr Georges	Investigateur					X
04	Dr Ait Hssain	Investigateur					X
	Dr Gazuy	Investigateur					X
05	Dr Hamidfar Roy	Investigateur					X
	Dr Barnoud	Investigateur					X
	Dr Bonadona	Investigateur					X
	Dr Hammer	Investigateur					X
	Dr Remy	Investigateur					X
	Dr Schwebel	Investigateur					X
	Dr Tabah	Investigateur					X
06	Dr Karoubi	Investigateur					X
07	Dr Isaline Coquet	Investigateur principal					X
08	Pr Jean-Yves Lefrant	Investigateur principal	CHU Nîmes	Place du Pr Robert DEBRE	30029 Nîmes		X
09	Dr Stéphane Legriel	Investigateur principal	CH Versailles<br>Hôpital André Mignot	177, rue de Versailles	78157 LE CHESNAY Cedex		X
	Dr Bedos	Investigateur					X
	Dr Troché	Investigateur					X
	Dr Bruneel	Investigateur					X
	Dr Henry-Lagarrigue	Investigateur					X

Liste des centres AmBiDex - V2_080508

N° centre	Investigateur	Fonction	Adresse 1	Adresse 2	CP - VILLE	Déclaration initial	CV joint
01	Pr Elie Azoulay	Investigateur principal	Hôpital Saint Louis	1 Avenue Claude Vellefaux	75010 PARIS	X	
	Dr WILLENS	Investigateur					X
02	Pr Michel Wolff	Investigateur principal	Hôpital Bichat	46 rue Henri Huchard	75018 PARIS		
	Dr Montravers	Investigateur					X
03	Dr Olivier Leroy	Investigateur principal	Hôpital Guy Chatilez	155, rue du Président Coty B.P. 619	59208 TOURCOING	X	
	Dr Boussekey	Investigateur					X
	Dr Georges	Investigateur					X
04	Pr Bertrand Souweine	Investigateur principal	Hôpital Gabriel Montpied	58 rue Montalembert	63003 CLERMONT-FERRAND Cedex 1	X	
	Dr Ait Hssain	Investigateur					X
	Dr Gazuy	Investigateur					X
05	Pr Jean François Timsit	Investigateur principal	CHU Grenoble Hôp A. MICHALLON	BP 217	38043 GRENOBLE CEDEX 09	X	
	Dr Hamidfar Roy	Investigateur					X
	Dr Barnoud	Investigateur					X
	Dr Bonadona	Investigateur					X
	Dr Hammer	Investigateur					X
	Dr Remy	Investigateur					X
	Dr Schwebel	Investigateur					X
	Dr Tabah	Investigateur					X
06	Pr Yves Cohen	Investigateur principal	Hôpital Avicenne	125 route de Stalingrad	93009 BOBIGNY	X	
	Dr Karoubi	Investigateur					X
07	Dr Jean Carlet	Investigateur	Hôpital Saint-Joseph	185, rue Raymond Losserand	75674 PARIS CEDEX 14	X	
	Dr Isaline Coquet	Investigateur principal					X
08	Pr Jean-Yves Lefrant	Investigateur principal	CHU Nîmes	Place du Pr Robert DEBRE	30029 Nîmes		X
09	Dr Stéphane Legriel	Investigateur principal	CH Versailles Hôpital André Mignot	177, rue de Versailles	78157 LE CHESNAY Cedex		X
	Dr Bedos	Investigateur					X
	Dr Troché	Investigateur					X
	Dr Bruneel	Investigateur					X
	Dr Henry-Lagarrigue	Investigateur					X
