# Supplementary material for: Weekly high-dose liposomal amphotericin B (L-AmB) in critically ill septic patients with multiple Candida colonization: The AmBiDex study
Source: PLoS One. 2017 May 22;12(5):e0177093. doi: 10.1371/journal.pone.0177093 (PMC5439673; doi:10.1371/journal.pone.0177093)
Supplement: S3 File — (PDF) [file pone.0177093.s003.pdf]

CONFIDENTIEL

NOM DE L'ÉTUDE: AMBIDEX

PRODUIT À L'ÉTUDE: AmBisome® (amphotéricine B liposomale)

FORME GALENIQUE/DOSAGE: 10 mg/kg

TITRE DE L'ÉTUDE: AmBiDex – Traitement préemptif des colonisations multiples à *candida* chez des patients de réanimation présentant un sepsis.

INVESTIGATEUR Coordinateur: Pr Elie AZOULAY
Service de Réanimation médicale
Hôpital Saint Louis
1 avenue Claude Vellefaux
75010 PARIS
France
Tél. : 01 42 49 94 21

PROMOTEUR: FOVEA
3 Bis Chemin de la Jonchère
92500 Rueil Malmaison
France
Tel. : 01 47 14 04 95

CHEF DE PROJET: Dr Jean François DREYFUS

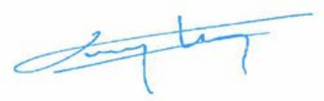

26 JUIN 2008

Signature du Chef de Projet *Date*

PROPRIETE EXCLUSIVE DU GROUPE FOVEA NE DOIT ETRE COMMUNIQUE, HORS DU GROUPE, QU'AUX PERSONNES QUI, DE PAR LEUR FONCTION ET/OU LEUR ROLE DANS L'ETUDE, DOIVENT AVOIR ACCES AU CONTENU INTEGRAL DU PROTOCOLE.

J'ai lu ce protocole et ses annexes et j'accepte de conduire l'étude selon ses dispositions.

Signature de l'Investigateur Principal *Date*

AMENDEMENT AU PROTOCOLE N° 2

JUSTIFICATION DE L'AMENDEMENT

Cet amendement n°2 au protocole a 4 objectifs :

- Prolonger la période de recrutement, en raison du nombre insuffisant de patients inclus à ce jour,
- Modifier le critère d'inclusion concernant la créatinémie, en vue de permettre l'inclusion de patients ayant une créatinémie comprise entre 220 et 300 µmol/L, ceux-ci étant très représentés dans la population cible de l'étude. L'inclusion de ces patients sera laissée à l'appréciation de l'investigateur, eu égard au rapport bénéfice/risque pour le patient.
- Modifier le critère d'exclusion concernant les traitements antifongiques antérieurs reçus par le patient, et n'exclure que les patients ayant reçu de l'Ambisome® depuis leur admission en service de réanimation
- Préciser le délai d'obtention du nombre de sites colonisés
- Ajouter, aux visites 1, 4, 5 et 6 le dosage du béta-D-glucane, marqueur prédictif des infections fongiques

SECTIONS Calendrier de l'étude ET Strucuture administrative de l'étude :

Début de l'étude : Septembre 2007
Fin de l'étude : Mars 2008

Modifié par :
Début de l'étude : Septembre 2007
Fin prévisionnelle de l'étude : Avril 2009 (dernier patient dernière visite)

SECTION 1.5 Problématique de l'étude

Une étude américaine récente place les infections à *candida spp.* au quatrième rang des infections nosocomiales les plus rencontrées avec un taux de mortalité de 40%. De plus, les infections à *candida spp.* surviennent majoritairement dans les services de réanimation (48, 49). La colonisation à *candida* n'est pas liée à un accroissement de mortalité mais le continuum colonisation – infection à *candida*, démontré en 1994 par Pittet et ses collègues souligne l'importance de la prévention d'une invasion chez les patients à haut risque de candidose invasive (16). De plus, le retour d'une hémoculture positive à *candida* signant une candidémie fréquemment mortelle est un événement tardif et peu sensible.
Ces faits sont en faveur d'un traitement antifongique précoce qui permettrait de traiter efficacement une candidose systémique à une étape où les techniques de diagnostic n'ont pas la sensibilité nécessaire pour affirmer ou infirmer l'infection. Ce traitement serait mené chez des patients hospitalisés en réanimation avec une colonisation par *candida* dans au moins deux sites et à haut risque de candidose invasive.

Modifié par :
Une étude américaine récente place les infections à *candida spp.* au quatrième rang des infections nosocomiales les plus rencontrées avec un taux de mortalité de 40%. De plus, les infections à *candida spp.* surviennent majoritairement dans les services de réanimation (48, 49). La colonisation à *candida* n'est pas liée à un accroissement de mortalité mais le continuum colonisation – infection à *candida*, démontré en 1994 par Pittet et ses collègues souligne l'importance de la prévention d'une invasion chez les patients à haut risque de candidose invasive (16). De plus, le retour d'une hémoculture positive à *candida* signant une candidémie fréquemment mortelle est un événement tardif et peu sensible.
Ces faits sont en faveur d'un traitement antifongique précoce qui permettrait de traiter efficacement une candidose systémique à une étape où les techniques de diagnostic n'ont pas la sensibilité nécessaire pour affirmer ou infirmer l'infection. Ce traitement serait mené chez des patients hospitalisés en réanimation avec une colonisation par *candida* dans au moins deux sites **(au minimum un site extra digestif colonisé au moment de l'inclusion du patient, le deuxième pouvant être localisé dans un délai de 96 heures suivant son inclusion)** et à haut risque de candidose invasive.
Par ailleurs, le Béta-D-glucane sera utilisé pour étayer le diagnostic de l'infection fongique invasive (50) chez les patients inclus dans cette étude.

SECTION 1.6 Objectifs de l'étude :

Objectifs secondaires
Les objectifs secondaires consistent à évaluer les paramètres suivants :
- Morbidité selon trois critères :
  - Durée d'hospitalisation
  - Durée de séjour en réanimation
  - Nombre de jours sans défaillance organique
- Incidence d'infection fongique invasive selon les critères de l'EORTC-MSG modifiés
- Survie à 28 jours

Modifié par :
Objectifs secondaires
Les objectifs secondaires consistent à évaluer les paramètres suivants :
- Morbidité selon trois critères :
  - Durée d'hospitalisation
  - Durée de séjour en réanimation
  - Nombre de jours sans défaillance organique
- Incidence des infections fongiques invasives selon les critères de l'EORTC-MSG modifiés, **et prenant en compte l'utilisation du beta-D-glucane comme critère supplémentaire du diagnostic des infections fongiques invasives**
- Survie à 28 jours

SECTION 3.1 Critères d'inclusion :

Age $\geq$ 18 ans
Ventilation mécanique depuis plus de 48 h
Patients présentant une défaillance d'organe en plus de celle requérant la ventilation mécanique
Colonisation à *candida* d'au moins 2 sites dont 1 non digestif
Suspicion de sepsis nosocomial
Mise en place d'une nouvelle antibiothérapie pour la suspicion de sepsis nosocomial
Consentement de la personne de confiance au moment de l'inclusion

Modifié par :
Age $\geq$ 18 ans
Ventilation mécanique depuis plus de 48 h
Patients présentant une défaillance d'organe en plus de celle requérant la ventilation mécanique
Colonisation à *candida* d'au moins 2 sites dont un non digestif **: après documentation d'un premier site extra-digestif (poumon, urines, muqueuses, redons …), la mise en évidence d'un deuxième site devra obligatoirement intervenir dans un délai de 96 heures**
Suspicion de sepsis nosocomial
Mise en place d'une nouvelle antibiothérapie pour la suspicion de sepsis nosocomial
Consentement de la personne de confiance au moment de l'inclusion

SECTION 3.2 Critères de non inclusion :

Traitement par antifongiques par voie orale (décontamination digestive sélective) ou par voie systémique dans les 15 jours précédant l'inclusion
Patients répondant aux critères de mise en œuvre d'un traitement antifongique classique
Patients moribonds (SAPS à l'inclusion >65)
Patients neutropéniques ou receveurs d'une greffe de cellules souches hématopoïétiques ou d'organe solide ou bénéficiant d'une chimiothérapie
Présence d'une infection fongique documentée (prouvée ou probable) selon les critères de l'EORTC/MSG modifiés
Créatininémie > 220 µmol/L ou hémodialyse
Grossesse

Modifié par :
Traitement **par Ambisome® depuis l'admission dans le service de réanimation pour l'épisode de sepsis actuel**
Patients répondant aux critères de mise en œuvre d'un traitement antifongique classique
Patients moribonds (SAPS à l'inclusion >65)
Patients neutropéniques ou receveurs d'une greffe de cellules souches hématopoïétiques ou d'organe solide ou bénéficiant d'une chimiothérapie
Présence d'une infection fongique documentée (prouvée ou probable) selon les critères de l'EORTC/MSG modifiés
Hémodialyse ou Créatininémie > 300 µmol/L, avec pour les valeurs de Créatininémie comprises entre 220 et 300 µmol/L une inclusion laissée au jugement de l'investigateur eu égard au rapport bénéfice/risque
Grossesse

SECTION 5.8 Recueil des informations tout au long de l'étude :

	V1 (J1)	V2 (J2)	V3 (J3)	V4 (J8)	V5 (J14)	V6 (J21)	V7 (J28 ou Fin d'essai)
Signature du consentement éclairé avant toute procédure liée à l'étude	X						
Démographie	X						
Critères d'inclusion et de non inclusion	X						
Comorbidités à l'inclusion	X						
Signes vitaux	X	X	X	X	X	X	X
Score SOFA	X	X	X	X	X	X	X
Critères de sepsis	X	X	X	X	X	X	X
Laboratoire	X	X	X	X	X	X	X
Colonisation à candida	X	2X /semaine					
Survenue d'infection fongique		X	X	X	X	X	X
Administration du produit à l'étude	X			X			
Traitements concomitants	X	X	X	X	X	X	X
Evénements indésirables	X	X	X	X	X	X	X
Recueil survie	X	X	X	X	X	X	X

Modifié par :

Une partie des échantillons de sang prélevés lors des visites V1, V4, V5 et V6 (7 ml à chaque visite) sera conservée jusqu'à la fin de l'étude (dernière visite du dernier patient) dans le centre investigateur, puis envoyé au laboratoire de l'Hôpital St Louis, Avenue Claude-Vellefaux - 75010 Paris, en vue du dosage du Béta-D-Glucane. Ces prélèvements sanguins ne seront pas utilisés à des fins d'analyse génétiques.

	V1 (J1)	V2 (J2)	V3 (J3)	V4 (J8)	V5 (J14)	V6 (J21)	V7 (J28 ou Fin d'essai)
Signature du consentement éclairé avant toute procédure liée à l'étude	X						
Démographie	X						
Critères d'inclusion et de non inclusion	X						
Comorbidités à l'inclusion	X						
Signes vitaux	X	X	X	X	X	X	X
Score SOFA	X	X	X	X	X	X	X
Critères de sepsis	X	X	X	X	X	X	X
Laboratoire	X	X	X	X	X	X	X
Colonisation à candida	X	2X /semaine					
Survenue d'infection fongique		X	X	X	X	X	X
Administration du produit à l'étude	X			X			
Traitements concomitants	X	X	X	X	X	X	X
Evénements indésirables	X	X	X	X	X	X	X
Recueil survie	X	X	X	X	X	X	X
**Dosage du Beta-D-Glucane**	X			X	X	X	

SECTION 6.3.2 Critères secondaires d'évaluation :

Les critères secondaires d'évaluation **de l'efficacité** comprennent :

- L'évaluation des événements indésirables non liés au traitement, survenus durant les 28 jours de l'étude.
- L'évaluation des paramètres de morbidité suivants :
- Durée de séjour dans l'unité de Soins Intensifs
- Durée de séjour à l'hôpital
- Nombre de jours sans défaillance d'organe
- L'incidence des infections fongiques invasives selon les critères de l'EORTC-MSG
- L'incidence d'autres infections nosocomiales subséquentes
- Le taux de mortalité à 28 jours

Modifié par :

Les critères secondaires d'évaluation comprennent :

- L'évaluation des événements indésirables non liés au traitement, survenus durant les 28 jours de l'étude.
- L'évaluation des paramètres de morbidité suivants :
- Durée de séjour dans l'unité de Soins Intensifs
- Durée de séjour à l'hôpital
- Nombre de jours sans défaillance d'organe
- L'incidence des infections fongiques invasives selon les critères de l'EORTC-MSG, **et prenant en compte l'utilisation du beta-D-glucane comme critère supplémentaire du diagnostic des infections fongiques invasives**
- L'incidence d'autres infections nosocomiales subséquentes
- Le taux de mortalité à 28 jours

SECTION 9 Références bibliographiques :

Ajouté :

50. Z. Odabasi, G. Mattiuzzi, E. Estey, H. Kantarjian, F. Saeki, R. J. Ridge, P. A. Ketchum, M.A. Finkelman, J. H. Rex and L. Ostrosky-Zeichner

Beta-d-Glucan as a Diagnostic Adjunct for Invasive Fungal Infections : Validation, Cutoff Development, and Performance in Patients with Acute Myelogenous Leukemia and Myelodysplastic Syndrome

Synopsis de l'étude :

Modifié en consequence par :

**Titre de l'étude**	AmBiDex – Traitement préemptif des colonisations multiples à *candida* chez des patients de réanimation présentant un sepsis
**Justification**	La candidémie est associée à une mortalité élevée or, il n'existe pas de procédure diagnostique qui permette d'anticiper la survenue de cette pathologie, mais il a été démontré, chez les patients chirurgicaux en soins intensifs, qu'il existait un continuum qui va de la colonisation à l'infection par *candida*. Il est donc urgent de développer des stratégies thérapeutiques lorsqu'un patient présente des colonisations de plusieurs sites qui coexistent avec des facteurs de risque comme la défaillance de plusieurs organes (y compris la nécessité d'une ventilation assistée) et un état septique nosocomial sévère.<br>La quasi−totalité des patients qui ont présenté une candidémie présentaient une colonisation par *candida*. et il semble que la colonisation soit associée à une prolongation de la durée du séjour en soins intensifs.<br>L'hypothèse de base de cette étude est que les patients en Unité de Soins Intensifs présentant une colonisation de plusieurs sites ont un risque élevé de candidose invasive et que l'administration précoce d'un agent antifongique empêchera l'apparition d'une candidose invasive.<br>Une administration hebdomadaire d'AmBisome® pourrait représenter une option thérapeutique importante que l'on peut justifier sur la base des résultats pharmacocinétiques et du profil pharmacodynamique, car elle associe un schéma d'administration simple à un large spectre d'activité thérapeutique.
**Nombre de centres prévus**	9 centres en France
**Objectif**	Principal :<br>▪ Tolérance et sécurité d'emploi de l'AmBisome® 10 mg/kg en une administration hebdomadaire (2 perfusions à une semaine d'intervalle)<br><br>Secondaires :<br>• Évaluation des paramètres de morbidité suivants :<br>Durée de séjour dans l'Unité de Soins Intensifs<br>Durée de séjour à l'hôpital<br>Nombre de jours sans défaillance d'organe<br>• Incidence des infections fongiques invasives selon les critères de l'EORTC-MSG modifiés, **et prenant en compte l'utilisation du beta-D-glucane comme critère supplémentaire du diagnostic des infections fongiques invasives**<br>• Incidence d'autres infections nosocomiales subséquentes<br>• Taux de mortalité à 28 jours

**Plan expérimental**	Étude pilote, multicentrique, réalisée en ouvert
**Nombre de patients**	30
**Population cible**	Patients en unité de soins intensifs présentant une colonisation de plusieurs sites par *candida* dans le contexte d'une défaillance d'au moins deux organes (y compris la nécessité d'une assistance respiratoire) et d'un état septique nosocomial sévère
**Traitement**	AmBisome® 10 mg/kg (amphotéricine B liposomale)
**Durée du traitement**	2 perfusions à une semaine d'intervalle
**Diagnostic et principaux critères d'éligibilité**	1. Age ≥ 18 ans<br>2. Ventilation depuis plus de 48 heures<br>3. Patients présentant une défaillance d'organe en plus de la ventilation mécanique<br>4. Colonisation à *candida* d'au moins 2 sites dont un non digestif **: après documentation d'un premier site extra-digestif (poumon, urines, muqueuses, redons …), la mise en évidence d'un deuxième site devra obligatoirement intervenir dans un délai de 96 heures**<br>5. Suspicion de sepsis nosocomial<br>6. Mise en place d'une nouvelle antibiothérapie pour la suspicion de sepsis nosocomial<br>7. Consentement de la personne de confiance au moment de l'inclusion
**Critères de non−inclusion**	1. Traitement **par Ambisome® depuis l'admission dans le service de réanimation pour l'épisode de sepsis actuel**<br>2. Patients répondant aux critères de mise en œuvre d'un traitement antifongique classique<br>3. Patients moribonds (SAPS à l'inclusion >65)<br>4. Patients neutropéniques ou receveurs d'une greffe de cellules souches hématopoïétiques ou d'organe solide ou bénéficiant d'une chimiothérapie<br>5. Présence d'une infection fongique documentée (prouvée ou probable) selon les critères de l'EORTC/MSG modifiés<br>6. **Hémodialyse ou Créatininémie > 300 µmol/L, avec pour les valeurs de Créatininémie comprises entre 220 et 300 µmol/L une inclusion laissée au jugement de l'investigateur eu égard au rapport bénéfice/risque**<br>7. Grossesse

**Informations recueillies et procédures**	V1 (J1)<br>Consentement éclairé d'un proche<br>Données démographiques<br>Vérification des critères d'inclusion et de non inclusion (comorbidités, critères de défaillance, score SOFA, état septique)<br>Signes vitaux avant et après perfusion<br>Examens de laboratoire<br>**Beta-D-Glucane**<br>Facteurs de risque (colonisation des sites habituellement surveillés dans le service)<br>Test de grossesse si applicable<br>Traitements concomitants<br>*Administration du produit à l'étude (durée, quantité administrée, procédure)*<br>Evènements indésirables<br>Survie<br><br>Recueil des informations sur la colonisation fongique des sites testés à V1, 2 fois par semaine<br><br>V2 (J2)<br>Signes vitaux, score SOFA, critères d'un sepsis, examens de laboratoire<br>Survenue d'infection fongique invasive, survie<br>Traitements concomitants, évènements indésirables<br><br>V3 (J3)<br>Signes vitaux, score SOFA, critères d'un sepsis, examens de laboratoire<br>Survenue d'infection fongique invasive, survie<br>Traitements concomitants, évènements indésirables<br><br>V4 (J8)<br>Signes vitaux, score SOFA, critères d'un sepsis, examens de laboratoire **et Beta-D-Glucane**<br>Survenue d'infection fongique invasive, survie<br>Traitements concomitants, évènements indésirables<br>*Administration du produit à l'étude*<br>Événements indésirables au cours et au décours de la perfusion<br><br>V5 (J14)<br>Signes vitaux, score SOFA, critères d'un sepsis, examens de laboratoire **et Beta-D-Glucane**<br>Survenue d'infection fongique invasive, survie<br>Traitements concomitants, évènements indésirables<br><br>V6 (J21)<br>Signes vitaux, score SOFA, critères d'un sepsis, examens de laboratoire **et Beta-D-Glucane**<br>Survenue d'infection fongique invasive, survie<br>Traitements concomitants, évènements indésirables<br><br>V7 (J28 ou J SORTIE) VISITE FINALE<br>Signes vitaux, score SOFA, critères d'un sepsis, examens de laboratoire<br>Survenue d'infection fongique invasive, survie<br>Traitements concomitants, évènements indésirables
