# Supplementary material for: Weekly high-dose liposomal amphotericin B (L-AmB) in critically ill septic patients with multiple Candida colonization: The AmBiDex study
Source: PLoS One. 2017 May 22;12(5):e0177093. doi: 10.1371/journal.pone.0177093 (PMC5439673; doi:10.1371/journal.pone.0177093)
Supplement: S4 File — (PDF) [file pone.0177093.s004.pdf]

2. Synopsis

NAME OF COMPANY<br>FOVEA<br>3 bis, chemin de la Jonchère<br>92500 Rueil-Malmaison<br>NAME OF FINISHED PRODUCT<br>AmBisome®<br>NAME OF ACTIVE INGREDIENT<br>Liposomal Amphotericin B	SUMMARY TABLE<br>Referring to Part ……<br>of the Dossier:<br>Volume:<br>Page:<br>Reference:	FOR NATIONAL AUTHORITY USE ONLY:

TITLE OF STUDY:

Pre-emptive Antifungal Therapy of Multiple-Site *Candida* Colonisation In Patients With ICU-acquired Severe Sepsis – The AmBiDex study.

INVESTIGATORS:

Principal French Coordinator: Elie Azoulay.

STUDY CENTER(S):

Seven (7) ICU in France.

PUBLICATION (REFERENCE):

Not applicable

STUDIED PERIOD:

First Patient First Visit: 2 april 2008

Last Patient Last Visit: 6 april 2009

PHASE OF DEVELOPMENT:

Pilot study.

OBJECTIVES:

The primary objective of this study was to evaluate the safety and tolerance of AmBisome® 10mg/kg in a weekly administration (two weeks treatment).

The secondary objectives of this study were as follows:
- To evaluate morbidity parameters as listed in the following: ICU stay length, hospital stay length and number of days without organ failures
- To assess the incidence of Invasive Fungal Infections (IFI) according to EORTC-MSG criteria (β D-Glucan was also measured as predictive marker of fungal infection)
- To assess other subsequent nosocomial infections
- To evaluate the mortality rate at day 28

METHODOLOGY:

This study was conducted as a pilot, multicenter, prospective open trial in ICU patients with multiple-site *Candida* colonisation and severe underlying disease responsible for two or more organ failures (including need for mechanical ventilation) and severe nosocomial sepsis.

All patients received once weekly intravenous administration of liposomal amphotericin B, AmBisome® over 2 to 4 hours at a dose of 10 mg/kg. Two administrations of AmBisome® were performed (at D1 and D8). A period of 21 days followed the second AmBisome®

NAME OF COMPANY<br>FOVEA<br>3 bis, chemin de la Jonchère<br>92500 Rueil-Malmaison<br>NAME OF FINISHED PRODUCT<br>AmBisome®<br>NAME OF ACTIVE INGREDIENT<br>Liposomal Amphotericin B	SUMMARY TABLE<br>Referring to Part ……<br>of the Dossier:<br>Volume:<br>Page:<br>Reference:	FOR NATIONAL AUTHORITY USE ONLY:

administration. During the first 7 days of this period, the patient was considered under treatment because of the persistence of AmBisome® in the body; the follow-up period (14 days) was considered as beginning 8 days after the second AmBisome® administration. For the patient, the total planned duration of the study was thus of 28 days.

NUMBER OF PATIENTS (PLANNED AND ANALYZED):

Total number of patients planned: 30
Total number of patients included: 21
Total number of patients analyzed: 20

DIAGNOSIS AND MAIN CRITERIA FOR INCLUSION/EXCLUSION:

INCLUSION:

Subjects who met all of the following inclusion criteria were eligible to participate in this study:

- Age ≥ 18 years
- Mechanical ventilation for longer than 48 hours
- At least one organ failure in addition to respiratory failure requiring mechanical ventilation
- *Candida* colonisation of at least one or more site in addition to the gastrointestinal tract (after documentation of a first extra-digestive site, the second site should be revealed in less than 96 hours)
- Suspicion of nosocomial sepsis
- Initiation of a new course of antibiotic therapy for this suspected nosocomial sepsis
- Consent from a trusted person at the Inclusion Visit

TEST PRODUCT, DOSE, AND MODE OF ADMINISTRATION; BATCH NUMBER:

Each flask of AmBisome® contained 50 mg of amphotericin B.
Method of administration: intravenous administration once weekly.
Batch number: n° CLI5378 with a peremption date of 31 december 2009

DURATION OF TREATMENT:

Two weeks

REFERENCE THERAPY, DOSE AND MODE OF ADMINISTRATION; BATCH NUMBER:

Not applicable.

CRITERIA FOR EVALUATION:

EFFICACY:

The secondary criteria of this study included:

- Assessment of unrelated Adverse Event occurring during the 28 days of the study
- Assessment of morbidity parameters as listed in the following: ICU stay length, hospital

NAME OF COMPANY<br>FOVEA<br>3 bis, chemin de la Jonchère<br>92500 Rueil-Malmaison<br>NAME OF FINISHED PRODUCT<br>AmBisome®<br>NAME OF ACTIVE INGREDIENT<br>Liposomal Amphotericin B	SUMMARY TABLE<br>Referring to Part ……<br>of the Dossier:<br>Volume:<br>Page:<br>Reference:	FOR NATIONAL AUTHORITY USE ONLY:

stay length and number of days without organ failures
- Assessment of the incidence of Invasive Fungal Infections (IFI) according to EORTC-MSG criteria (β D-Glucan was also measured as predictive marker of fungal infection)
- Assessment of other subsequent nosocomial infections
- Assessment of the mortality rate at day 28.

SAFETY:

The safety and tolerance of AmBisome® in patients with ICU-acquired severe sepsis were assessed recording the incidence of related AE occurring during the 28 days of the study. Other safety data (biological parameters, vital signs …) were described in the analysis if necessary.
AEs were tabulated according to PT (Preferred Term) and SOC (System Organ Class) using the MedDRA classification version 11.0. These tables presented the number of patients with AE as well as the frequency of AE. AEs were classified by seriousness, intensity (mild, moderate, severe), evolution (recovered, recovered with sequelae, ongoing, death), action taken to the study medication (none, withdrawal from the study, increase of the duration of infusion, other) and relationship to the study treatment (not related, potentially related).

STATISTICAL METHODS:

Descriptive statistics were used to summarize all endpoints of the study except the survival analysis which was performed using the Kaplan-Meyer method.

All AEs recorded during the study were analysed, listed and coded according to MedDRA version 11.0. The proportion of subjects with at least one AE—overall, by SOC and by PT—was presented. In addition, subjects with a potentially related AE, a serious AE, an AE leading to discontinuation of the study medication were also tabulated together with the intensity of the AE and AEs associated with abnormal laboratory results. The following frequency distributions were provided: all AEs by causality, severity, seriousness, outcome (including withdrawal from treatment or from study), by SOC, by decreasing frequency. Concerning laboratory parameters, a difference was calculated between the raw value at baseline and the value at each Visit. All changes were presented at D1, D4 and D28 (except creatinine which is presented at all Visits). Vital signs and weight were also presented at all Visits as the change between the value at baseline and the value at each Visit. Other safety measures such as duration of infusion, security, morbidity criteria, incidence of a systemic fungal infection, incidence of other nosocomial infections, sepsis criteria, SOFA score, *Candida* colonisation and onset of fungal infection were described. Finally, a 28-day survival analysis was performed using the Kaplan-Meyer method.

Classical statistical analyses were performed with SAS® Software version 9.1.3.

To describe quantitative variables, the following statistics were computed, unless otherwise indicated: number, mean, standard deviation (SD), minimum, maximum, median, 95%

5. Ethics

5.1 Independent Ethics Committee (IEC) or Institutional Review Board (IRB)

Prior to initiation at each site, the protocol, its amendments, the information notice and patient informed consent form were reviewed and approved by an Independent Ethics Committee (IEC), the "Patient's Protection Committee (PPC)" of Ile-de-France VI (Groupe Hospitalier Pitié-Salpêtrière) before enrolment of patients. Also, prior to initiating the research, the Sponsor had submitted its research file to the "*Agence Française de Sécurité Sanitaire des Produits de Santé* (AFSSAPS)" and had been granted, by the relevant Health Authorities, authorisation to implement the research in accordance with the "*Code de la Santé Publique*" (CSP) (article L1123-8).

5.2 Ethical Conduct of the study

The study was performed in accordance with the relevant parts of the ICH Guidelines for Good Clinical Practices (GCP), the European directives on clinical trials, specifically the 2001/20/CE directive, the Declaration of Helsinki, and the pertinent individual country laws/regulations.

5.3 Patient Information and Consent

It was up to the Investigator to give each patient's legally authorized representative full and adequate verbal and written information about the objectives and the procedures of the study and the possible risks involved, prior to the patient's inclusion in the study. They were informed about their rights to withdraw from the study at any time and for any reason without sanction, penalty, or loss of benefits to which they were otherwise entitled and that withdrawal from the study would not jeopardize their future medical care. Written Patient Information was given to each patient's legally authorized representative before enrolment. Furthermore, it was up to each Investigator to obtain a signed Informed Consent Form (ICF) from each patient's legally authorized representative prior to performing any study-related procedures. The Patient Information and ICF were updated or amended whenever new important information became available that could be relevant to the patient.

A sample consent form can be found in Appendix 16.1.3.

6. Investigators and study Administration Structure

Curriculum vitae for study personnel are included in Appendix 16.1.4.

Coordinator	Pr Elie Azoulay<br>Hôpital Saint-Louis – Service de Réanimation Médicale<br>1 avenue Claude Vellefaux<br>7501-0 Paris - France<br>01 42 49 94 21<br>elie.azoulay@sls.ap-hop-paris.fr
Sponsor (FOVEA)	Dr Franck Sévenier<br>3 bis, chemin de la Jonchère<br>92500 Rueil-Malmaison – France<br>01 47 14 40 71<br>f.sevenier@fovea-group.com
Pharmacovigilance Manager	Dr Franck Sévenier<br>3 bis, chemin de la Jonchère<br>92500 Rueil-Malmaison – France<br>01 47 14 40 71<br>f.sevenier@fovea-group.com
Monitoring (FOVEA)	Naïma Dahmouni<br>3 bis, chemin de la Jonchère<br>92500 Rueil-Malmaison – France<br>01 47 14 40 91<br>n.dahmouni@fovea-group.com
Statistical analysis (FOVEA)	Roger-Louis Calvez<br>3 bis, chemin de la Jonchère<br>92500 Rueil-Malmaison – France<br>01 47 14 40 91<br>r.calvez@fovea-group.com
Statistical analysis (FOVEA)	Stéphanie Mohsen<br>3 bis, chemin de la Jonchère<br>92500 Rueil-Malmaison – France<br>01 47 14 40 91<br>s.mohsen@fovea-group.com

7. Introduction and rationale of the study

Medical background

Although intensive care patients present many risk factors for invasive candidiasis [1,2], candidaemia is a rare event. In fact only 2 out of every 1,000 patients admitted to intensive care present candidaemia [3,4], though this figure may reach 10 in surgical Intensive Care Units (ICU) [5,6]. These figures are very different from those from North America [7].

Nevertheless, during recent years, intensive care specialists have become particularly concerned about fungal infections and antifungal therapies. The following five items point up the clinical relevance of fungal infections in critical care, and explain the paradox between an infrequent event (invasive candidiasis) and the particular attention paid by intensive care specialists to *Candida*–related disease:

1) Mortality due to invasive candidiasis is high, with a high ascribable mortality, suggesting that preventive treatment is probably superior to curative treatment [4,8];

2) Blood cultures become positive for *Candida* (candidaemia) at a relatively late stage and has little diagnostic value, pointing up the need for preventive strategies as well as for research to identify an earlier diagnostic strategy [2];

3) Although most cases of candidaemia are caused by *Candida albicans*, the SENTRY program and NNIS study emphasise the importance of the emergence of strains with reduced susceptibility to fluconazole (*C Glabrata and C Kruseï*) [7,9,10,11,12], although the situation is less worrying in Europe [13] where fluconazole is still used for first-line treatment [14,15];

4) In 1994, Pittet *et al*. demonstrated that there is a continuum from *Candida* colonisation to infection in surgical intensive care patients, and proposed a colonisation index (ratio of the number of colonized sites to the number of sampled sites) as an independent risk factor for candidaemia with a genotypically identical strain to that colonizing the patient beforehand [16]. However, these results have not been confirmed in other studies since their first publication and more particularly, not in patients in the medical ICU setting.

However, a recent study demonstrated a relationship between multiple *Candida* colonisation sites (especially in the lung and digestive tract) and candidaemia in non-neutropenic medical ICU patients [17];

5) In recent years, studies have shown the pathogenicity of *Candida* in the peritoneal cavity of surgical ICU patients operated for gastrointestinal perforation, necrotizing pancreatitis, or suture line breakdown [18,19,20]. Likewise, risk factors have been identified [21] and prophylactic therapies have been validated [22]. The presence of *Candida* in the peritoneum has a different significance to its presence in the lungs [23-25] and urine [26].

The above discussion underlines the potential value of early “pre-emptive” or prophylactic antifungal treatment in non-immunocompromised ICU patients.

Candidiasis and critical care: clinical forms and prognosis.

None of the various clinical forms of invasive *Candida* infection is specific to critical care. The main characteristics of invasive candidiasis in critical care are its rarity [4,27,28] and severity with the need for life support after organ failure in patients who often have significant comorbidity. Many authors have underlined the lateness of onset of candidaemia and the major need to develop a preventive therapeutic strategy, coupled with a need for further research on immunological diagnostic methods, the results of which have been disappointing to date [2,27,28,29].

In non-neutropenic critical care patients, multiple colonisation prior to fungal infection seems to characterize the surgical ICU patient. Hence, if *Candida* catheter infection is more frequently seen in critical care, it is not clear whether this is a secondary site or if it is the original cause of the fungaemia. All contaminated catheters must nevertheless be systematically removed.

Several descriptive studies on *Candida* colonisation have been performed in the surgical intensive care setting: such colonisation is present in more than half the patients [29], in most patients with candidaemia [30], and seems to be associated with longer stays in the intensive care unit [29]. *Candida albicans* is the main species responsible for this colonisation [16,29,30], with *Candida glabrata* and *Candida kruseï* of reduced susceptibility to antifungal agents only accounting for about 10% of cases. Three features of such colonisation should be pointed out: firstly, colonisation is generally asymptomatic and must therefore be systematically screened for; secondly, *Candida* colonisation is a dynamic process—in addition to the number of colonized sites (and the colonisation index), how it changes over time is a parameter of major importance and vascular and tissue dissemination ensue after multiple colonisation; finally, the *Candida* colonisation index not only still needs to be validated in a broader population of surgical patients, but also in medical patients.

Interestingly, a recently published study carried out in a medical critical care department in Barcelona underlined the independent relationship between multiple *Candida* colonisation and invasive candidiasis [17]. This study, together with that of Jorge Garbino in Geneva [31], seems to suggest that the colonisation index is a useful parameter in medical resuscitation but this remains to be shown in a study specifically performed for this purpose.

Preventive antifungal therapies in non-neutropenic ICU patients.

Two preventive strategies have been suggested [2]. A summary descriptive diagram is given in Figure 1.

The first strategy, called prophylaxis, concerns ICU patients who have no *Candida* colonisation but who present risk factors such as corticosteroid therapy, dialysis, central catheters, broad spectrum antibiotic therapy or prolonged mechanical ventilation. This concept of antifungal prophylaxis is borrowed from that successfully used in leukaemia or bone marrow transplant recipients [32,33].

The second strategy corresponds to the institution of antifungal treatment in patients colonized at many sites by *Candida*. This is an early or pre-emptive measure based on the possibility that such multiple colonisation can lead to invasive candidiasis.

To date, only prophylactic strategies have been evaluated. Overall, an oral (nystatin or ketoconazole) or systemic (fluconazole) prophylactic agent can be used to decolonize critical care patients. However, prophylaxis has been shown to have inconsistent effects on the incidence of invasive candidiasis (burn patients [34], preterm infants [35] or patients with prolonged stay in surgical critical care [36]). In adults, only a single study—conducted on patients with septic shock caused by peritonitis—has shown that prophylaxis has a significant effect on mortality [37]. However, these studies have shown that the antifungal agents routinely used for prophylaxis are safe in intensive care patients, a major element when it comes to risk/benefit ratio.

FIGURE 1 - PROPOSED PREVENTIVE ANTIFUNGAL STRATEGIES

The strategies described in this diagram are not recommended at present.

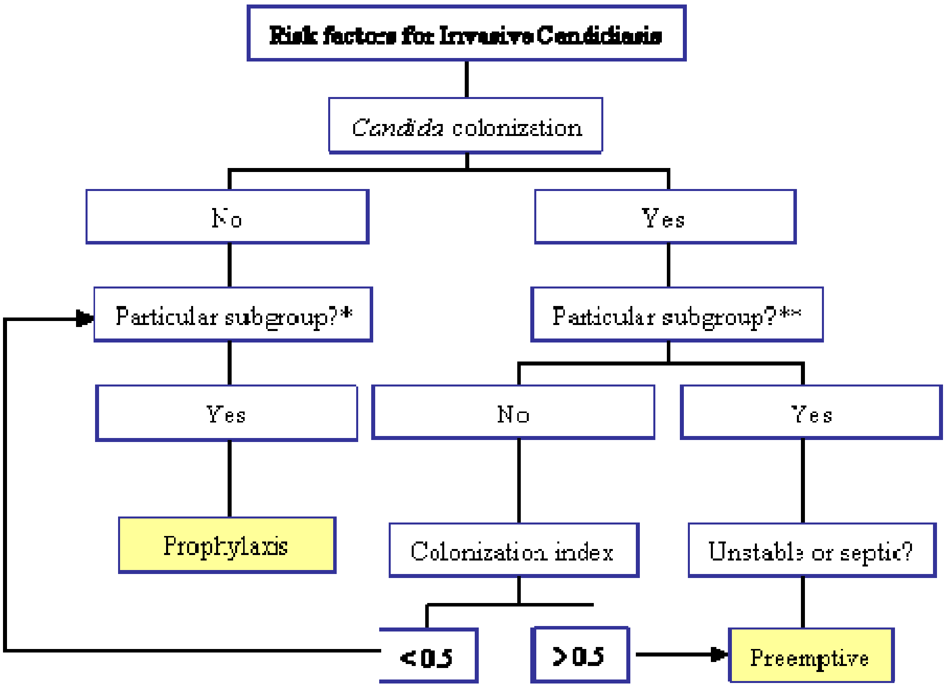

*

**

Liposomal Amphotericin B: AmBisome®

Amphotericin B is a broad spectrum antifungal agent covering, in particular, infections with most common *Candida spp.* [38].

New lipid amphotericin B formulations have recently been developed and one of them, liposomal amphotericin B (AmBisome®) has been shown in many trials to have a better safety profile than Conventional Amphotericin B (CAB).

A broad study comparing AmBisome® (3 mg/kg/day) with amphotericin B (0.6 mg/kg/day) in empirical treatment confirmed the superiority of AmBisome®, especially with respect to renal function (serum creatinine doubling of 19% for AmBisome® compared with 34% for CAB) [39].

In this large-scale trial, the total success rate was similar in the 2 groups according to a composite endpoint comprising survival 7 days after treatment, resolution of fever during neutropaenia, resolution of signs of fungal infection, absence of onset of IFI and premature treatment discontinuation. On the other hand the incidence of confirmed IFI was significantly lower in the AmBisome® group and this difference was even greater for allograft recipients and patients with neutropaenia lasting for 7 days or more.

In the treatment of systemic and/or deep *Aspergillus* and *Candida* mycoses in adults and children, the recommended dose of AmBisome® in the Summary of Product Characteristics (SPC) is 3 mg/kg/day. However, the most significant fungal infections (invasive aspergillosis and invasive candidiasis) are still associated with high mortality rates [40,41] requiring innovating therapeutic strategies.

The rationale for administering AmBisome® at doses higher than those in the Marketing Authorisation is based on the demonstration of a dose-response relationship in animal models of invasive fungal infection (J Adler-Moore and Anaissie) and on the dose-escalation study by Walsh *et al.* [42]. High dosages of AmBisome®, administered under real conditions (patients with probable or documented IFI) may be used with a good safety profile, specifically a dosage of 10 mg/kg/day with a maximum Cmax and AUC. This dose escalation was continued up to 15 mg/kg/day without the Maximum Tolerated Dose (MTD) being reached in adults, and up to 10 mg/kg/day in children.

The AmBiload study, a randomised double-blind study on modified EORTC criteria, showed that the efficacy of the AmBisome® 10mg/kg/day arm was similar to that of the AmBisome® 3 mg/kg/day arm in the treatment of filamentous fungal infections with serum creatinine doubling rates during treatment of about 30%, i.e. identical to those found in the Walsh dose escalation study [43]. All the product-related adverse events which occurred were known and documented, mainly nephrotoxicity, hypokalaemia, hepatic abnormalities and infusion-related events.

The AmBiload study made it possible to validate the daily dosing of AmBisome® at standard doses of 3 mg/kg/day in the treatment of invasive fungal infections by filamentous fungi and more specifically invasive aspergillosis with an efficacy at the end of the treatment of 50% and 12-week survival rates of 72%.

The Combistrat study justified the administration of AmBisome® 10 mg/kg/day in the treatment of invasive aspergillosis with a 12-week survival rate of 80%. This rate was not significantly different from the rate of 100% in the comparative arm combining the standard dose of AmBisome® with caspofungin [(44)]. This study was a pilot, multicentre, prospective, randomised trial comparing open-label AmBisome® 3 mg/kg/day combined with Cancidas® and AmBisome® 10 mg/kg/day, the two treatments being maintained for 14 days in the treatment of suspected invasive aspergillosis or aspergillosis confirmed by modified EORTC-MSG criteria.

At the end of treatment, the results of this study demonstrated that efficacy was significantly higher in the combination arm than in the high-dose arm (67% and 27% respectively, p=0.03). In addition, the 3-month survival rate was 100% in the combination arm versus 80% in the high-dose arm (non-significant). Regarding tolerance, more infusion-related adverse effects were observed in the monotherapy arm than in the combination arm on the 1st day of treatment.

This AmBisome® high-dose dosing regimen may allow doses to be given at intervals of up to one week while maintaining an effective plasma concentrations. In "*Biology of Blood and Marrow Transplantation*", Mehta *et al.* [(45)] published a study of prophylaxis in paediatric [≥ 10 years] allograft recipients using AmBisome® at the dose of 10 mg/kg/week. Fourteen patients were treated, none with a history of IFI. Only one patient presented a suspected fungal infection (on images with negative laboratory tests) which was successfully treated in an empirical manner. Pharmacokinetic measurements showed a distribution profile with high concentrations of AmBisome® which remained measurable on the 7th day after infusion. The safety profile was excellent without any documented signs of nephrotoxicity and, in particular, no significant rise in creatinine.

The Prophysome study [(46)], presented at EBMT in 2006 by Catherine Cordonnier, studied the safety of a prophylactic treatment regimen of high-dose AmBisome® administered weekly to two populations at high haematological risk (allograft recipients and acute leukaemia patients). This study showed excellent tolerance in the Acute Leukaemia arm with no product-related adverse event leading to premature study discontinuation. The study also pointed up the importance of good coordination between AmBisome® infusion dates and the hematological schedule of allograft recipients because of their specific conditioning.

The AlloAmBi Pro study underlined the feasibility of a prophylactic high-dose AmBisome® regimen with weekly infusion, in particular after corticosteroid therapy to treat graft-versus-host disease in non-myeloablative allogeneic graft recipients [(47)].

Specific problems of the study

A recent American study placed infections by *Candida spp.* fourth in the list of the most frequent nosocomial infections with a mortality rate of 40%. Moreover, most infections by *Candida spp.* occur in intensive care departments [(48,49)]. Colonisation with *Candida* is not linked to an increase in mortality but the existence of the *Candida* colonisation – infection continuum proposed in 1994 by Pittet *et al.* points up the importance of preventing invasion in patients at high risk of invasive candidiasis [(16)]. Moreover, *Candida*-positive blood cultures detecting candidaemia that often proves fatal are usually not obtained until a late stage and therefore have little clinical relevance.

These facts provide evidence in favour of early antifungal treatment which would make it possible to treat systemic candidiasis effectively at a stage when diagnostic techniques are insufficiently sensitive to confirm or rule out infection. This treatment will be given to intensive care patients with *Candida* colonisation at two sites at least and a high-risk of invasive candidiasis.

The AmBidex study is a multicentre, open-label prospective study of the pre-emptive treatment of multiple *Candida* colonisations in intensive care patients with sepsis.

The "pre-emptive" nature is defined by the existence of colonisation associated with a background of sepsis supposing possible dissemination although neither candidaemia nor even candidiasis may yet be detected. The purpose of this pre-emptive treatment (which by definition is non-curative at this stage) is to prevent the onset of candidaemia.

Unlike prophylactic treatment, the initiation of pre-emptive treatment is based on *Candida* colonisation. There is no consensus about any specific medicinal product in this indication.

The weekly administration of high-dose AmBisome®—which is possible because of the product's known pharmacokinetic profile—couples easy administration with broad-spectrum activity.

8. Study objectives

The ***primary objective*** of this study was to evaluate the safety and tolerance of AmBisome® 10 mg/kg in a weekly administration (two weeks of treatment).

The ***secondary objectives*** of this study were as follows:

- To evaluate the following morbidity parameters: ICU stay length, hospital stay length and number of days without organ failures
- To assess the incidence of Invasive Fungal Infections (IFI) according to EORTC-MSG criteria (β D-Glucan was also measured as predictive marker of fungal infection)
- To assess other subsequent nosocomial infections
- To evaluate the mortality rate at day 28.

9. Investigational Plan

9.1 Overall study Design and Plan

This study was conducted as a pilot, multicenter, prospective open trial in ICU patients with multiple-site *Candida* colonisation and severe underlying disease responsible for the failure of two or more organs (including need for mechanical ventilation) and severe nosocomial sepsis.

All patients received once-weekly intravenous liposomal amphotericin B, AmBisome® over 2 to 4 hours at a dose of 10 mg/kg. Two administrations of AmBisome® were performed (at D1 and D8). A period of 21 days followed the second AmBisome® administration. During the first 7 days of this period, the patient was considered as under treatment because of the persistence of AmBisome® in the body. The follow-up period (14 days) began 8 days after the second AmBisome® administration. For each patient, the planned total duration of the study was thus 28 days (Figure 1).

FIGURE 2 – AMBIDEX STUDY DIAGRAM

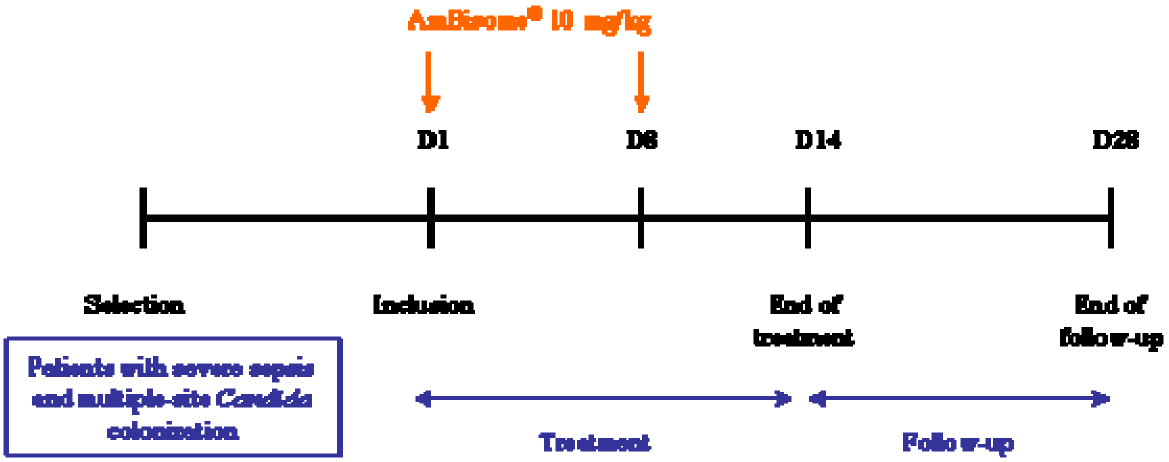

9.2 Discussion of study Design, Choice of Control Groups

This study assessed the safety and tolerance in patients for whom the participating ICU had no defined protocol for antifungal therapy when the patient was included in the study.

There was no comparison treatment. A second analysis with a placebo population (same inclusion criteria as above without administration of AmBisome® in pre-emptive treatment) was conducted in a retrospective way.

9.2.1 Selection of study Population

9.2.1.1 Inclusion Criteria

Patients fulfilling the following criteria were eligible for inclusion in the study:

- Age ≥ 18 years
- Mechanical ventilation for longer than 48 hours
- At least one organ failure in addition to respiratory failure requiring mechanical ventilation
- *Candida* colonisation of at least one site in addition to the gastrointestinal tract (after documentation of a first extra-digestive site, the second site should be revealed in less than 96 hours)
- Suspicion of nosocomial sepsis
- Initiation of a new course of antibiotic treatment for this suspected nosocomial sepsis
- Consent from the trustworthy person at inclusion Visit.

9.2.1.2 Non-inclusion Criteria

Patients fulfilling any of the following criteria were excluded from study participation:

- Patients treated by AmBisome® since the admission in the ICU for treatment of sepsis
- Patients corresponding to the criteria for the implementation of classic antifungal treatment
- Dying patients (SAPS at inclusion > 65)
- Patients with neutropaenia or receiving a marrow or solid organ transplant or on chemotherapy
- Proved or likely IFI according to the criteria of EORTC
- Patients under haemodialysis or with blood creatinine > 300 µmol/L (for patients with blood creatinine between 220 and 300 mol/L, inclusion was left to the Investigator's discretion)
- Pregnant patients

9.2.2 Removal of Patients from Therapy or Assessment

All patients having received AmBisome® (even one incomplete dosage) were included in the statistical analysis.

When a patient was prematurely withdrawn from the study the Investigator had to follow its patient until the end of the study (28 days after the first infusion).

If a blood culture was positive during the study or at the end of study, it was up to the Investigator to prescribe the most appropriate antifungal treatment.

9.3 Treatments

9.3.1 Treatments Administered

Amphotericin B is a macrocyclic polyene antifungal antibiotic produced by *Streptomyces nodosus*. Liposomes are closed, spherical vesicles created by mixing specific proportions of amphophilic substances such as phospholipids and cholesterol so that they arrange themselves into multiple concentric bilayer membranes when hydrated in aqueous solutions. AmBisome® consists of these unilamellar bilayer liposomes with amphotericin B intercalated within the membrane. Due to the nature and quantity of amphophilic substances used, and the lipophilic moiety in the amphotericin B molecule, the drug is an integral part of the overall structure of the amphotericin B liposomal liposomes. Amphotericin B (liposomal) contains true liposomes that are <100 nm in diameter.

AmBisome® was administered at 10 mg/kg/week by intravenous infusion once weekly using a controlled infusion device, over a period of approximately 2 hours. For the first infusion, the following instructions were respected: during the first 20 minutes of injection, the administration was twice as slow as the normal administration. The remaining infusion was then administered to a normal speed (the first infusion being longer than the following ones). The patient was particularly followed to prevent any AE.

One previous medication preventing the toxicity related to the infusion [antipyretic (paracetamol only) and antihistaminics (dexchlorpheniramine only)] was eventually administered to the discretion of the Investigator at patients having already presented AE. When the patient knew no previous episode of toxicity, the use of paracetamol or dexchlorpheniramine was allowed in case of mild signs or symptoms.

If these signs or symptoms were more severe or if they required another treatment, the patient was withdrawn from the study. In case of discomfort persisting after the first 30 minutes, the infusion was administered on a duration of 3 hours instead of 2. AmBisome® was exclusively used in infusion in a hospital environment, under constant medical supervision.

9.3.2 Identity of Investigational Product

Information about the treatment was described in the SPC.

AmBisome® had to be reconstituted using sterile water for injection, USP (without a bacteriostatic agent). It was not reconstituted with saline or added saline to the reconstituted concentration or mixed with other drugs. The use of any solution other than those recommended, or the presence of a bacteriostatic agent in the solution, could cause precipitation.

AmBisome® had then to be diluted with 5% glucose injection to a final concentration of 0.2 - 2.0 mg/mL prior to administration.

The flasks of AmBisome® contained 50 mg of amphotericin B. The single-dose flasks were packaged in boxes containing 10 flasks with 10 sterile filters. Indeed, an in-line membrane filter was used for the intravenous infusion; the mean pore diameter of the filter was equal to 5 microns.

Unopened vials were stored at temperature comprised between 2°C and 25°C.

After reconstitution with sterile water, vials were stored at temperature comprised between 2°C and 8°C during 7 days or between 23°C and 27°C during 24 hours in the same flask.

After reconstitution with 5% glucose, vials were stored at temperature comprised between 2°C and 8°C during 7 days or between 23°C and 27°C during 72 hours in the same flask.

9.3.3 Method of Assigning Patients to Treatment Groups

Not applicable.

9.3.4 Selection of Doses in the study

A once-weekly dose of 10 mg/kg AmBisome® was chosen on the basis of the product's known pharmacokinetic profile and because this dosage has been shown to constitute an effective prophylactic regimen in clinical trials conducted in high-risk populations (allograft recipients and patients with acute leukaemia) (45,46,47).

9.3.5 Selection and Timing of Dose for Each Patient

Not applicable.

9.3.6 Blinding

Not applicable.

9.3.7 Prior and Concomitant Therapy

Concomitant treatments were reported in the Case Report Form (CRF).

Special attention was paid to concomitant nephrotoxic treatments.

For any interactions with other drugs, SPC had to be consulted.

9.3.8 Treatment Compliance

Compliance was assessed by the nursing staff reporting the administered dosage and the conditions of infusion.

9.4 Efficacy and Safety Variables

9.4.1 Efficacy and Safety Measurements Assessed and Flow Chart

The following data were recorded at each Visit (Figure 3).

FIGURE 3 – STUDY FLOW CHART

	V1 (D1)	V2 (D2)	V3 (D3)	V4 (D8)	V5 (D14)	V6 (D21)	V7 (D28 or end of study)
Signature of ICF(1)	X						
Demographics	X						
Inclusion and exclusion criteria	X						
Comorbidities at inclusion	X						
Pregnancy test (if applicable)	X						
Vital signs (2)	X	X	X	X	X	X	X
SOFA score	X	X	X	X	X	X	X
Sepsis criteria	X	X	X	X	X	X	X
Laboratory assessments	X	X	X	X	X	X	X
Candida colonisation (3)	X	Twice a week					
Onset of fungal infection		X	X	X	X	X	X
Administration of study product	X			X			
Concomitant treatments	X	X	X	X	X	X	X
Adverse events	X	X	X	X	X	X	X
Survival	X	X	X	X	X	X	X
β-D-Glucan dosage	X			X	X	X	

(1) By a close relative *(2) Before and after infusion*
(3) Sites where tests were made at the inclusion Visit were assessed during all the study

<u>Safety measurements</u>

Tolerance of the study medication was defined as follows: reactions related to the infusion (fever, shivers, dyspnoea, nausea, vomiting), renal toxicity (twofold increase in blood creatinine compared with the initial value and hypokalaemia ≤ 2.5 mmol) and other relevant AEs as defined by the Investigator.

The definitions used in the study were presented hereafter:

Adverse Event (AE): any untoward medical occurrence in a patient or clinical investigation subject administered a pharmaceutical product and which did not necessarily have to have a causal relationship. All AEs which were considered by the Investigators as direct consequences of the disorders requiring admission into the ICU were not reported in the CRF except: serious AEs, hypokalaemia ≤ 2.5 mmol/L, disorders of heart rhythm, oligoanuria < 400 ml, AEs occurring during the infusion of AmBisome® or within one hour after the end of infusion, and any relevant AE during the 28-day follow-up of the patient (as defined by the Investigator).

Serious Adverse Event (SAE)): a SAE was any untoward medical occurrence that, at any dose:

- Resulted in death,
- Was life threatening (patient at risk of death at the time of the event),
- Required inpatient hospitalisation or prolongation of existing hospitalisation,
- Resulted in persistence or significant disability/incapacity, or
- Was a congenital anomaly/birth defect
- Was medically significant.

In this study, the following were considered as SAEs: events leading to death, documented candidaemia, serious renal failure requiring dialysis, and any event considered as serious and unexpected by the Investigator during the 28-day follow-up of the patient.

Severe and Serious Adverse Event

The terms “severe” and “serious” were not synonymous: "severe" was used to describe the intensity (severity) of a specific event, which could be rated mild, moderate or severe. The event itself however could be of relatively minor medical significance (such as severe headache). Seriousness (and not severity) served as a guide for defining regulatory reporting obligations.

The **severity** of the event was evaluated by the following scale:

Mild: did not interfere with the usual patient activities

Moderate: partially prevented the patient to perform his/her usual activities

Severe: prevented totally the patient to perform his/her usual activities

The **relationship** to the experimental drugs was evaluated by using the following rules:

Probable/Likely: a clinical event, including laboratory test abnormality, with a reasonable time sequence to administration of the drug, unlikely to be attributed to concurrent disease or other drugs or chemicals, and which followed a clinically reasonable response on withdrawal.

None

If the AE met the SAE definition, the Investigator reported it immediately (24h) to the Sponsor's defined representative by any means.

Responsible of pharmacovigilance : Dr Franck SEVENIER
Mobile : 01 47 14 40 71
Fax : 01 47 16 11 210
Mail: f.sevenier@fovea-group.com

The declaration was made by phone followed by a written confirmation, by fax, or by sending an e-mail, and included at least the following elements: identification of the protocol, the patient, the Investigator and the nature of the SAE.

A specific form was available in the CRF for documenting and reporting the SAE. After the initial report, the SAE were promptly followed by a detailed writing report including useful medical results and if required additional follow-up reports in order to provide the relevant medical information on the case.

The responsible of pharmacovigilance of the Sponsor had to send to the AFSSAPS (at the latest under 7 calendar days for death or under 15 calendar days for any other SAE and\or unexpected) the descriptions received. The possible relationship to AmBisome® and the unexpected AE were determined with the Investigator.

For reported death, the Investigators supplied the Sponsor and the IRB/IEC with any additional requested information such as autopsy reports if available and terminal medical reports.

If the event was an SAE, the Investigators had to comply with the relevant national regulatory requirements related to the reporting of unexpected serious adverse events.

The Sponsor (FOVEA) expedited the reporting to all concerned Investigators/institutions, to the IRBs/IECs, where required, and to the regulatory authorities of all AE that were serious (SAE) or unexpected.

A particular attention was brought to the follow-up of the SAE until its resolution. If there was no resolution, the steering committee of the study determined the type and the duration of the follow-up.

The planning for submission of the periodic (PSR) and Annual Safety Reports (ASR) was:
- PSR: every 6 months October and April
- ASR: in April.

9.4.2 Appropriateness of Measurements

The parameters analysed correspond to standard measurements performed routinely in an ICU. Routine sites were selected for sampling to evaluate *Candida* colonisation, namely the lung, gastrointestinal tract, urine, mouth, pharynx and anus as well as the injury, wound or drain.

The safety and tolerance of AmBisome® in patients with ICU-acquired severe sepsis were assessed recording the incidence of related AE occurring during the 28 days of the study. The other safety data (biological parameters, vital signs …) were described in the analysis if necessary.

The AE were tabulated according to the PT (Preferred Term) and the SOC (System Organ Class) terms using the MedDRA classification version 11.0. These tables presented the number of patients with AE as well as the frequency of AE. The AE were classified by seriousness, intensity (mild, moderate, severe), evolution (recovered, recovered with sequelae, ongoing, death), action taken to the study medication (none, withdrawal from the study, increase of the duration of infusion, other) and the relation to the study medication (not related, potentially related).

9.4.3 Drug Concentration Measurements

Not applicable.

9.5 Data Quality Assurance

Monitoring

The monitors of FOVEA conducted initiation Visits at each study center after approval of the study was obtained from the IEC and Competent Authorities to discuss the clinical protocol and review data collection procedures, safety monitoring and reporting procedures, and regulatory requirements.

Monitoring Visits to the study centers were conducted periodically during the study performance, in order to:

- Review the status of the study with respect to patient enrolment, occurrence of AE, etc.
- Ensure that the clinical Investigators continued to meet their contractual, clinical and regulatory obligations with regard to protocol compliance (and compliance to protocol amendments, if applicable), adherence to regulatory and ethical requirements and the protection of the patients' rights and safety
- Ensure the scientific integrity of the study by reviewing the integrity and completeness of the data collected on the CRF on the basis of the raw data
- Review the completeness and accuracy of the study Site Records

Source documents were reviewed for verification of agreement with data on the CRF. The study center guaranteed direct access to source documents by designated FOVEA personnel or their designees and appropriate regulatory authorities. Monitoring was conducted according to the internal Standard Operating Procedures. The study was also subject to a quality assurance audit by FOVEA or its designees, as well as inspection by appropriate regulatory authorities. It was important that the Investigator and the relevant study personnel were available during the monitoring Visits and possible audits and that sufficient time was devoted to the process.

Data management

Data management methods employed double data entry with automatic controls on the database (SAS®) and data acquisition was audited on 10% of the CRF with a validation threshold lower than 1‰. Upon completion of data entry, the data were reviewed by FOVEA to ensure acceptable accuracy and completeness. If necessary, the study site was contacted for corrections and/or clarifications.

Any data management queries and items not adequately explained were returned to the Investigator by the monitor for clarification/correction. The Investigator ensured that data queries were dealt with promptly. Copies of all data changes and clarifications were retained by the Investigator and filed with the CRF.

The coding of AE and concomitant medications was performed by the CRO FOVEA. Concomitant medications were coded using the World Health Organisation Drug Dictionary (WHO-DRUG) 2005 classification and AE were coded using MedDRA version 11.0. Following coding, data underwent medical review to ensure that verbatim AE and concomitant medication terms were coded appropriately, the data in the database accurately reflected the CRF and that final data were retained and archived appropriately.

Steering committees

A Steering Committee was set up to ensure the scientific soundness of the study and that the study objectives were met.

This Steering Committee was composed by:

- Renée Grillot: mycologist at CHU Grenoble
- Professor Elie Azoulay: principal coordinator
- Professor Michel Wolff: principal Investigator
- A Sponsor responsible
- A Gilead Sciences responsible (providing the study product to the Sponsor)

The Steering Committee was responsible for:

- Validation of protocol and study documentation, and protocol amendments if deemed necessary
- Final statistical analysis and clinical report validation
- Publication policy and manuscript writing and/or validation. All requests for data presentation of publication should receive the previous approval from the committee.

The Steering Committee met regularly (3 to 4 times a year) or under request from the Sponsor.

Record retention

According to the legislation the on-site documents and those received by the promoter had to be kept during at least 15 years. The promoter had to be consulted before any destruction of documents relative to the study or remained on sites.

9.6 Statistical Methods Planned in the Protocol and Determination of Sample Size

9.6.1 Statistical and Analytical Plan

The ***safety population*** was defined as all patients who received at least one dose of AmBisome® (complete or not).

The ***Per-Protocol (PP) population*** was defined as all patients having received the 2 planned AmBisome® injections and having no major deviation (as defined during the Data Review).

Descriptive statistics were used to summarize all endpoints of the study except the survival analysis performed using using the Kaplan-Meyer method.

Disposition of subjects, demographics (Age, gender, height, weight), baseline characteristics (Medical history, physical examination, *Candida* colonisation, hospitalisation, failure, laboratory assessments, concomitant treatments and vital signs) were presented using descriptive statistics.

All AE recorded during the study were analysed, listed and coded according to MedDRA version 11.0. The proportion of subjects with AE, overall, by SOC and by PT was presented. In addition, subjects with potentially related AE, serious AE, AE leading to discontinuation of the study medication, intensity of AE and laboratory abnormalities AE were also tabulated. The following frequency distributions were provided: all AE by causality, severity, seriousness, outcome (including withdrawal from treatment or from study) by SOC by decreasing frequency. Concerning laboratory parameters, a difference was calculated between the raw value at baseline and the value at each Visit. All changes were presented at D1, D4 and D28 (except creatinine presented at all Visits). Vital signs and weight were also presented at all Visits as the change between value at baseline and value at each Visit. The other safety measures as duration of infusion, security, morbidity criteria, incidence of a systemic fungal infection, incidence of other nosocomial infection, sepsis criteria, SOFA score, *Candida* colonisation and onset of fungal infection were described. Finally, the 28-days survival analysis was performed using the Kaplan-Meyer method.

Classical statistical analyses were performed with SAS® Software version 9.1.3.

To describe quantitative variables, the following statistics were computed, unless otherwise indicated: number, mean, standard deviation (SD), minimum, maximum, median, 95% confidence interval and missing values.

To describe qualitative variables by treatment group, the following statistics were computed, unless otherwise indicated: number, percentage and missing values.

All tests were two-sided at 0.05 α level, if necessary.

9.6.2 Determination of Sample Size

According to ICH E9 statistical principles for clinical trials, the predefined number of subjects had to be sufficient to assess the primary objective of the study, i.e. the assessment of the safety and tolerance of the study product. Furthermore, the assessment criterion used to evaluate this objective was only descriptive with the exhaustive presentation of AE occurring during the follow-up of patients. No statistical test requiring a minimum number of patients was necessary for assessment of the primary criterion of the study.

Given the number of participating centres, frequency of inclusion and planned study duration, the number of subjects for this non comparative pilot study was set at 30.

9.7 Changes in the Conduct of the study or Planned Analyses

The protocol was accepted on 29th August 2007 by the PPC of Ile-de-France – Groupe Hospitalier Pitié-Salpêtrière.

Amendment n°1 (24th June, 2008): This amendment, including the recruitment of more Investigators, did not affect the protocol.

Amendment n°2 (26th June, 2008):

- Increase of the period of recruitment (End-of-study in April 2009 instead of March 2008 - few patients were recruited in March 2008)
- Modification of the inclusion criteria related to blood creatinine: patients with blood creatinine comprised between 220 and 300 µmol/L could be included (Inclusion was kept at the Investigator's discretion)
- Modification of the exclusion criteria concerning the previous antifungal treatments received by the patients: only patients having received AmBisome® since their admission in ICU were excluded from the study
- Addition of the duration related to the number of colonised sites
- At Visits D1, D4, D5 and D6, addition of the dosage of β D-Glucan, predictive marker of fungal infections.

Change in the conduct of planned analyses:

Two secondary objectives planned in the protocol could not be analysed :

- Assessment of the number of days without organ failure
- Calculation of the incidence of other nosocomial infections

In fact, the case record form did not plan to collect the data needed to assess these 2 criteria, even a posteriori.
